# Design, synthesis, *in vitro* potent antiproliferative activity, and kinase inhibitory effects of new triarylpyrazole derivatives possessing different heterocycle terminal moieties

**DOI:** 10.1080/14756366.2019.1653292

**Published:** 2019-08-27

**Authors:** Mahmoud M. Gamal El-Din, Mohammed I. El-Gamal, Mohammed S. Abdel-Maksoud, Kyung Ho Yoo, Chang-Hyun Oh

**Affiliations:** a Pharmaceutical and Drug Industries Research Division, National Research Centre, Dokki-Giza, Egypt;; b Department of Medicinal Chemistry, College of Pharmacy, University of Sharjah, Sharjah, UAE;; c Sharjah Institute for Medical Research, University of Sharjah, Sharjah, UAE;; d Department of Medicinal Chemistry, Faculty of Pharmacy, University of Mansoura, Mansoura, Egypt;; e Chemical Kinomics Research Center, Korea Institute of Science and Technology, Seoul, Republic of Korea;; f Center for Biomaterials, Korea Institute of Science and Technology, Seoul, Republic of Korea;; g Department of Biomolecular Science, University of Science and Technology, Daejeon, Republic of Korea

**Keywords:** Antiproliferative activity, morpholine, substituted piperazine, triarylpyrazole, V600E-B-RAF

## Abstract

A new series of triarylpyrazole derivatives having different heterocycle terminal groups have been designed and synthesised. Compounds **1h**–**j** and **1l** exhibited the highest mean percentage inhibition against the 58 cancer cell lines at a concentration of 10 μM, and thus were next examined in 5-dose testing mode to detect their IC_50_ value. The four compounds showed stronger antiproliferative activities upon comparing their results with sorafenib as a reference compound. Among them, compounds **1j** and **1l** possessing *N-*ethylpiperazinyl and *N-*benzylpiperazinyl terminal moiety through ethylene linker showed the greatest values of mean percentage inhibition (97.72 and 107.18%, respectively) over the 58-cell line panel at 10 μM concentration. The IC_50_ values of compound **1j** over several cancer cell lines were in submicromolar scale (0.26 ∼ 0.38 μM). Moreover, the compounds **1j** and **1l** showed highly inhibitory activities (99.17 and 97.92%) against V600E-B-RAF kinase.

## Introduction

Cancer is considered as one of the dangerous world health problems. Statistics according to World Health Organization (WHO) report shows that the global cancer burden is about 18.1 million new cases and 9.6 million deaths in 2018, and the number of deaths can increase to more than 13 million deaths in 2030^1^. Despite the remarkable achievements in the diagnostic and therapeutic techniques nowadays, cancer could be assessed as the second leading cause of death globally, about 1 in 6 deaths is due to cancer after cardiovascular diseases^2^^,^^3^. Considering many reports and publications on the synthesis of anti-cancer agents, there is no medicine achieving 100% efficacy and potency. Therefore, there is still a continuous need for more drug inventions leading to more potent and efficient anti-cancer compounds with new structures or novel mechanism of action to overcome the adverse effects associated with present chemotherapeutics in cancer treatment, such as toxicity and drug resistance.

**Figure 1.**
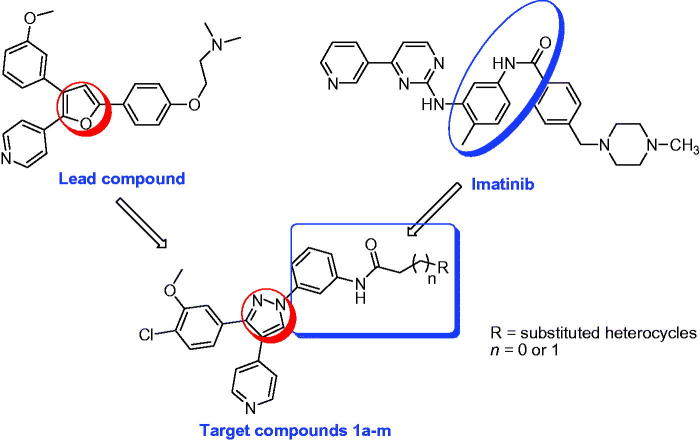
Structures of the lead compound, imatinib, and the target compounds **1a–m**.

Metastatic melanoma is among the serious types of cancer that could lead to the limited survival time of less than one year^4^. Melanoma is associated with important pathway RAS–RAF-MEK-ERK^5^. One mutation that is most frequent in melanoma is mutated BRAF kinase (V600E-B-RAF)^6^. It is reported also that sorafenib (Nexavar^®^) targets ERK signal transduction pathway that was also involved in many cancer types including melanoma. Many research articles have nowadays outlined the potential antiproliferative activity against melanoma would be through inhibition of V600E-B-RAF which is so frequent in melanoma^7^. Imatinib (Gleevec^®^, Figure 1) as an example of anticancer agents having arylamides^8–11^ terminal moiety that is used for chronic myeloid leukaemia (CML) treatment with minimised adverse effects^12^. It has been reported in clinical trials for the management of different diseases like GIST, meningioma, ovarian cancer, non-small cell lung cancer (NSCLC), thyroid cancer, and breast cancer along with other drugs^13^.

Furthermore, much consideration has been directed to the chemistry and biological activities of 1,3,4-triarylpyrazole derivatives. Diverse chemical entities having 1,3,4-triarylpyrazole nucleus have been lately turned up as potent antiproliferative agents^14–18^.

In the current article, we outline the synthesis of a new series of 1,3,4-triarylpyrazole nucleus bearing *meta*-amino group possessing heterocyclic terminal moieties linked by methylene or ethylene carbonyl spacer. In this study, we have replaced the furan ring of the lead compounds by its bioisosteric nucleus pyrazole, and we have tried the *meta* substitution for comparison of the *para* ones of the lead compounds. Our target compounds were examined against NCI-58 cancer cell line panel of nine different cancer type and their *in vitro* antiproliferative activities were reported. The most active compounds were examined against V600E-B-RAF and JNK kinases for detecting their possible molecular mechanism of action (Figure 1).

## Experimental

### General

The synthesised compounds were purified using silica gel chromatography of size (0.040–0.063 mm, 230–400 mesh) and solvents of technical grade. Bruker Avance 400 spectrometer utilising tetramethylsilane as an internal standard was used to record ^1^H NMR and ^13^C NMR spectra. Hypodermic syringes were used to transfer Solvents and liquid reagents. All the made compounds were examined by HPLC and found to have purity of >95%. All solvents and reagents were commercially available and used without further purification.

Compounds **3** till **8** were synthesised as reported in the literature^15^^,^^16^.

### 3-(3-(4-Chloro-3-methoxyphenyl)-4-(pyridin-4-yl)-1H-pyrazol-1-yl)benzenamine (8)

^1^H NMR (400 MHz, CDCl_3_) *δ* 8.52 (s, 2H), 8.06 (s, 2H), 8.32 (d, 1H, *J=* 8.0 Hz), 7.23–7.14 (m, 5H), 7.53–7.45 (m, 3H), 7.04 (brs, 2H), 6.98–6.95 (m, 1H), 6.44 (d, 1H, *J=* 4.8 Hz), 6.31 (d, 1H, *J=* 7.6 Hz), 4.03 (brs, 2H), 3.78 (s, 3H); ^13^C NMR (100 MHz, CDCl_3_) *δ* 154.9, 149.9, 147.9, 140.3, 132.3, 130.3, 127.5, 122.8, 122.6, 121.5, 119.8, 113.8, 112.3, 108.7, 105.9, 56.8; MS *m/z*: 377.2 (M^+^).

### 2-Chloro-N-(3-(3-(4-chloro-3-methoxyphenyl)-4-(pyridin-4-yl)-1H-pyrazol-1-yl)phenyl)-acetamide (9a) *or* 3-chloro-N-(3–(3-(4-chloro-3-methoxyphenyl)-4-(pyridin-4-yl)-1H-pyrazol-1-yl)phenyl)propanamide (9b)

To a cooled solution of compound **8** (0.5 g, 1.325 mmol) in anhydrous methylene chloride (15 ml) at −10 °C, triethylamine (148 mg, 1.4575 mmol) was added dropwise within 5 min then either chloroacetyl chloride or chloropropionyl chloride were added dropwise keeping the reaction mixture temperature at −10 °C for another 5 min .To the reaction mixture, water (15 ml) was added, then reaction mixture was swirled then the organic layer was separated. The water layer was further extracted with methylene chloride once again. The combined organic layers were combined, washed with brine and dried using anhydrous Na_2_SO_4_. Then evaporated under reduced pressure to give the target compounds that were used in the next step without further purification with yield 65%. Compound **9a**: ^1^H NMR (400 MHz, DMSO-d_6_) *δ* 8.85 (d, 1H, *J=* 8.0 Hz), 8.08 (brs, 2H), 7.76–7.53 (m, 6H), 7.29–7.16 (m, 3H), 4.13 (s. 2H), 3.86 (s, 3H); ^13^C NMR (100 MHz, DMSO-d_6_) *δ* 166.8, 159.5, 148.2, 141.2, 140.4, 139.8, 139.7, 135.1, 131.7, 129.2, 128.8, 127.7, 125.9, 123.1, 120.5, 116.4, 115.1, 111.2, 105.7, 55.6; MS *m/z*: 453.2 (M^+^ + 1). Compound **9b**: ^1^H NMR (400 MHz, DMSO-d_6_) *δ* 8.85 (d, 1H, *J=* 8.0 Hz), 8.08 (brs, 2H), 7.76–7.53 (m, 6H), 7.29–7.16 (m, 3H), 4.13 (s. 2H), 3.86 (s, 3H); MS *m/z*: 466.5 (M^+^ + 1).

### N-(3-(3-(4-Chloro-3-methoxyphenyl)-4-(pyridin-4-yl)-1H-pyrazol-1-yl)phenyl)-2-(piperidin-1-yl)acetamide (1a)

To a solution of **9a** (100 mg, 0.175 mmol) in anhydrous methylene chloride (5 ml), triethylamine (20 mg, 0.193 mmol) was added at 0 °C, stirred for 15 min, then morpholine (18.3 mg, 0.21 mmol) was added dropwise. The reaction mixture was stirred for 24 h at r.t. Upon completion of the reaction, the mixture was dried and then was partitioned between ethyl acetate and aqueous phase. Followed by separation of the organic layer and extraction of the aqueous layer with organic solvent (3 × 10 ml) was repeated. The collected organic layers were washed with NaCl solution (5%) and the organic solvent was evaporated under vacuum. The residue was purified using column chromatography (silica gel, hexane:ethyl acetate 4:1 v/v) to give the required product. ^1^H NMR (400 MHz, CDCl_3_) *δ* 8.48 (d, 2H, *J=* 4.8 Hz), 8.14(s, 1H), 7.45 (d, 1H, *J=* 4.8 Hz), 7.39 (d, 1H, *J=* 8 Hz), 7.38 (t, 1H, *J=* 8 Hz), 7.28 (d, 1H, *J=* 8 Hz), 7.19–7.18 (m, 2H), 7.07 (br s, 1H), 7.01 (dd, 1H, *J=* 2.0, 8.0 Hz), 3.74 (s, 3H), 3.22 (s, 2H), 2.65 (brs, 4H), 1.67 (t, 4H, *J=* 5.2 Hz),1.47 (brs, 2H); ^13^C NMR (100 MHz, CDCl_3_) *δ* 170.6, 154.9, 150.0, 139.6, 139.3, 138.9, 132.1, 130.1, 130.1, 127.6, 122.9, 122.8, 121.5, 120.3, 117.9, 114.9, 112.3, 110.2, 62.2, 56.3, 55.2, 25.7, 23.2; MS *m/z*: 502.43 (M^+^ + 1).

Compounds **1b–g** were synthesised from **9a** and appropriate amine derivatives as reported in the synthesis of **1a**.

### N-(3-(3-(4-Chloro-3-methoxyphenyl)-4-(pyridin-4-yl)-1H-pyrazol-1-yl)phenyl)-2-morpholino-acetamide (1b)

^1^H NMR (400 MHz, CDCl_3_) *δ* 9.33 (s, 1H), 8.56 (d, 2H, *J=* 4.8 Hz), 8.03(s, 1H), 7.57 (d, 1H, *J=* 7.6 Hz), 7.51–7.45 (m, 2H), 7.36 (d, 1H, *J =* 8.4 Hz), 7.29–7.27 (m, 2H), 7.14 (s, 1H), 7.09 (d, 1H, *J =* 8.0 Hz), 3.80 (m, 7H), 3.23 (s, 2H), 2.69–2.67 (m, 4H); ^13^C NMR (100 MHz, CDCl_3_) *δ* 168.1, 154.6, 150.1, 149.7, 140.8, 139.9, 138.7, 132.2, 130.5, 130.2, 127.6, 122.9, 122.8, 122.2, 117.9, 114.9, 112.3, 110.2, 66.9, 62.4, 56.1, 50.8; MS *m/z*: 505.1 (M^+^ + 2).

### N-(3-(3-(4-Chloro-3-methoxyphenyl)-4-(pyridin-4-yl)-1H-pyrazol-1-yl)phenyl)-2–(4-methyl-piperazin-1-yl)acetamide (1c)

^1^H NMR (400 MHz, CDCl_3_) *δ* 9.28 (s, 1H), 8.58 (d, 2H, *J =* 8.4 Hz), 8.22 (d, 2H, *J=* 3.6 Hz), 7.59–7.56 (m, 2H), 7.46 (t, 1H, *J=* 8.0 Hz), 7.38 (d, 1H, *J=* 8.0 Hz), 7.29–7.27 (m, 2H), 7.15–7.08 (m, 2H), 3.83 (m, 3H), 3.31 (s, 2H), 2.94–2.90 (m, 8H), 2.63 (s, 3H); MS *m/z*: 517.42 (M^+^ + 1).

### N-(3-(3-(4-Chloro-3-methoxyphenyl)-4-(pyridin-4-yl)-1H-pyrazol-1-yl)phenyl)-2–(4-ethyl-piperazin-1-yl)acetamide (1d)

^1^H NMR (400 MHz, CDCl_3_) *δ* 9.29 (s, 1H), 8.58 (brs, 2H), 8.15 (d, 2H, *J=* 5.2 Hz), 7.59–7.56 (m, 2H), 7.47 (t, 1H, *J =* 8.0 Hz), 7.38 (d, 1H, *J=* 8.0 Hz), 7.16–7.08 (m, 4H), 3.83 (m, 3H), 3.27 (s, 2H), 2.83–2.50 (m, 10H), 1.25 (t, 3H, *J*= 7.6 Hz); ^13^C NMR (100 MHz, CDCl_3_) *δ* 169.0, 155.0, 150.1, 149.9, 139.9, 138.9, 132.1, 130.3, 130.0, 127.5, 122.3, 121.5, 120.3, 117.9, 114.8, 112.3, 110.2, 61.7, 56.1, 52.4, 52.2, 29.7; MS *m/z*: 531.0 (M^+^ + 1).

### N-(3-(3-(4-Chloro-3-methoxyphenyl)-4-(pyridin-4-yl)-1H-pyrazol-1-yl)phenyl)-2–(4-phenyl-piperazin-1-yl)acetamide (1e)

^1^H NMR (400 MHz, CDCl_3_) *δ* 8.48 (d, 2H, *J =* 4.8 Hz), 8.13 (brs, 1H), 7.63 (brs, 1H), 7.51–7.28 (m, 3H), 7.28–7.18 (m, 6H), 7.06 (s, 1H), 6.98 (d,1H, *J =* 8.0 Hz), 6.88–6.83 (m, 3H), 3.71 (s, 3H), 3.25–3.24 (m, 4H), 2.81 (s, 2H), 2.11–1.99 (m, 4H); ^13^C NMR (100 MHz, CDCl_3_) *δ* 170.7, 155.0, 149.6, 139.9, 138.1, 132.1, 130.9, 130.3, 130.0, 129.3, 128.9, 127.7, 123.0, 121.5, 120.3, 117.9, 116.5, 114.9, 112.3, 110.2, 61.7, 56.1, 52.4; MS *m/z*: 579.9 (M^+^ + 2).

### 2-(4-Benzylpiperazin-1-yl)-N-(3–(3-(4-chloro-3-methoxyphenyl)-4-(pyridin-4-yl)-1H-pyrazol-1-yl)phenyl)acetamide (1f)

^1^H NMR (400 MHz, CDCl_3_) *δ* 8.57 (d, 2H, *J=* 5.6 Hz), 7.78–7.75 (m, 4H), 7.59–7.53 (m, 4H), 7.45 (t, 2H, *J*= 4.0 Hz), 7.40 (d, 1H*, J*= 8.4 Hz), 7.14–7.07 (m, 2H), 3.84 (s, 3H), 3.31 (s, 2H), 3.10 (brs, 4H), 2.36 (brs, 4 H), 2.32 (brs, 2H); MS *m/z*: 593.9 (M^+^ + 2).

Compounds **1 g–m** were prepared from **9 b** and appropriate acid chloride as reported in the synthesis of **1a.**

### N-(3-(3-(4-Chloro-3-methoxyphenyl)-4-(pyridin-4-yl)-1H-pyrazol-1-yl)phenyl)-3-(piperidin-1-yl)propanamide (1 g)

^1^H NMR (400 MHz, CDCl_3_) *δ* 8.59 (brs, 1H), 8.36 (s, 1H), 8.19 (s, 1H), 7.57 (d, 1H, *J=* 8.0 Hz), 7.44 (t, 1H, *J=* 8.0 Hz), 7.36 (d, 2H, *J=* 8.0 Hz), 7.32–7.29 (m, 3H), 7.17 (s, 1H), 7.10 (d, 1H, *J=* 7.6 Hz), 3.83 (s, 3H), 3.22 (s, 2H), 2.84–2.67 (m, 6H), 1.79–1.28 (m, 8H); ^13^C NMR (100 MHz, CDCl_3_) *δ* 170.7, 154.9, 150.1, 149.9, 149.8, 140.1, 139.9, 132.6, 130.5, 130.2, 129.6, 127.6, 122.7, 120.5, 120.2, 117.7, 114.9, 112.2, 110.2, 56.1, 54.1, 53.6, 32.6, 25.7; MS *m/z*: 517.21 (M^+^ + 2).

### N-(3-(3-(4-Chloro-3-methoxyphenyl)-4-(pyridin-4-yl)-1H-pyrazol-1-yl)phenyl)-3-morpholino-propanamide (1 h)

^1^H NMR (400 MHz, CDCl_3_) *δ* 9.33 (s, 1H), 8.56 (d, 2H, *J=* 4.8 Hz), 8.03(s, 1H), 7.57 (d, 1H, *J=* 7.6 Hz), 7.51–7.45 (m, 2H), 7.46 (d, 1H, *J=* 8.4 Hz), 7.29–7.27 (m, 2H), 7.16 (s, 1H), 7.07 (d, 1H, *J=* 8.0 Hz), 3.75 (m, 9H), 3.23 (s, 2H), 2.69–2.67 (m, 4H); ^13^C NMR (100 MHz, CDCl_3_) *δ* 168.1, 154.6, 150.1, 149.7, 140.8, 139.9, 138.7, 132.2, 130.5, 130.2, 127.6, 122.9, 122.8, 122.2, 117.9, 114.9, 112.3, 110.2, 66.9, 62.4, 56.1, 50.8, 25.2; MS *m/z*: 518.43 (M^+^ + 1).

### N-(3-(3-(4-Chloro-3-methoxyphenyl)-4-(pyridin-4-yl)-1H-pyrazol-1-yl)phenyl)-3-(4-methyl-piperazin-1-yl)propanamide (1i)

^1^H NMR (400 MHz, CDCl_3_) *δ* 8.49 (d, 2H, *J=* 6.4 Hz), 8.29 (s, 1H), 8.15 (s, 1H), 7.49 (dd, 8H, *J=* 1.2, 8.0 Hz), 7.36 (t, 1H, *J=* 8.0 Hz), 7.28 (d, 1H, *J=* 4.8 Hz), 7.21 (d, 2H, *J=* 6.4 Hz), 7.07 (s, 1H), 7.03 (dd, 1H, *J=* 1.6, 8.0 Hz), 3.73 (s, 3H), 2.73–2.51 (m, 12H), 2.31 (s, 3H); ^13^C NMR (100 MHz, CDCl_3_) *δ* 170.7, 154.6, 149.9, 149.6, 140.6, 139.9, 139.8, 132.3, 130.1, 129.6, 127.6, 122.9, 122.6, 121.4, 120.1, 117.6, 114.1, 112.3, 110.2, 56.0, 55.1, 53.4, 53.1, 45.8, 32.5; MS *m/z*: 529.9 (M^+^ + 1).

### N-(3-(3-(4-Chloro-3-methoxyphenyl)-4-(pyridin-4-yl)-1H-pyrazol-1-yl)phenyl)-3-(4-ethyl-piperazin-1-yl)propanamide (1j)

^1^H NMR (400 MHz, CDCl_3_) *δ* 8.56 (d, 2H, *J=* 6.4 Hz), 8.29 (s, 1H), 8.15 (s, 1H), 7.49(dd, 8H, *J=* 1.2, 8.0 Hz), 7.36 (t, 1H, *J=* 8.0 Hz), 7.28 (d, 1H, *J=* 4.8 Hz), 7.21 (d, 2H, *J=* 6.4 Hz), 7.07 (s, 1H), 7.03 (dd, 1H, *J=* 1.6, 8.0 Hz), 3.73 (s, 3H), 2.73–2.51 (m, 12H), 2.31 (s, 3H); ^13^C NMR (100 MHz, CDCl_3_) *δ* 170.7, 154.6, 149.9, 149.6, 140.6, 139.9, 139.8, 132.3, 130.1, 129.6, 127.6, 122.9, 122.6, 121.4, 120.1, 117.6, 114.1, 112.3, 110.2, 56.0, 55; MS *m/z*: 545.9 (M^+^ + 2).

### N-(3-(3-(4-Chloro-3-methoxyphenyl)-4-(pyridin-4-yl)-1H-pyrazol-1-yl)phenyl)-3-(4-phenyl-piperazin-1-yl)propanamide (1k)

^1^H NMR (400 MHz, CDCl_3_) *δ* 8.58 (d, 2H, *J=* 6.0 Hz), 8.34 (s, 1H), 8.21 (s, 1H), 7.58 (d, 1H, *J=* 7.2 Hz), 7.43 (t, 1H, *J=* 8.0 Hz), 7.35–7.28 (m, 2H), 7.14 (s, 1H), 7.08 (dd, 1H, *J=* 2.0, 8.0 Hz), 6.96–6.93 (m, 6H), 3.79 (s, 3H), 2.96–2.65 (m, 12H); ^13^C NMR (100 MHz, CDCl_3_) *δ* 169.7, 155.1, 151.9, 150.9, 150.8, 146.9, 142.9, 140.0, 132.0, 130.3, 129.3, 127.5, 123.1, 122.4, 121.5, 120.5, 120.3, 117.6, 117.2, 116.4, 114.5, 112.2, 110.2, 56.1, 53.1, 52.4, 32.5; MS *m/z*: 593.43 (M^+^ + 1).

### 3-(4-Benzylpiperazin-1-yl)-N-(3–(3-(4-chloro-3-methoxyphenyl)-4-(pyridin-4-yl)-1H-pyrazol-1-yl)phenyl)propanamide (1 l)

^1^H NMR (400 MHz, CDCl_3_) *δ* 8.57 (d, 2H, *J=* 6.0 Hz), 8.36 (s, 1H), 8.21 (s, 1H), 7.58 (dd, 1H, *J=* 1.2, 9.2 Hz), 7.43 (t, 1H, *J=* 8.0 Hz), 7.33–7.29 (m, 5H), 7.16–7.11 (m, 2H), 3.79 (s, 3H), 3.58 (s, 2H), 2.81–2.58 (m, 12H); ^13^C NMR (100 MHz, CDCl_3_) *δ* 170.9, 156.9, 150.9, 150.8, 146.9, 142.9, 140.0, 137.6, 132.5, 130.3, 129.1, 127.6, 123.0, 121.5, 120.2, 117.6, 114.3, 112.3, 110.2, 62.9, 56.1, 53.1, 52.3, 32.6; MS *m/z*: 608.1 (M^+^ + 1).

### 3-(4-(4-Fluorobenzyl)piperazin-1-yl)-N-(3–(3-(4-chloro-3-methoxyphenyl)-4-(pyridin-4-yl)-1H-pyrazol-1-yl)phenyl) propanamide (1 m)

^1^H NMR (400 MHz, CDCl_3_) *δ* 8.49 (d, 2H, *J=* 6.0 Hz), 8.26 (s, 1H), 8.09 (s, 1H), 7.48 (d, 1H, *J=* 8.0 Hz), 7.36 (t, 1H, *J=* 8.0 Hz), 7.26 (d, 1H, *J=* 8.0 Hz), 7.19–7.17 (m, 5H), 7.05–7.01 (m, 2H), 6.92 (t, 3H, *J=* 8.8 Hz), 3.71 (s, 3H), 3.44 (s, 2H), 2.74–2.51 (m, 12H); ^13^C NMR (100 MHz, CDCl_3_) *δ* 170.7, 154.9, 149.9, 149.8, 140.6, 140.0, 133.2, 132.2, 130.6, 130.3, 127.1, 123.0, 122.7, 121.5, 120.3, 117.5, 115.3, 114.3, 112.3, 110.2, 61.9, 56.1, 53.4, 53.2, 32.5; MS *m/z*: 626.0 (M^+^ + 2).

### Cancer cell line screening at the NCI

Screening against the cancer cell lines was carried out at the National Cancer Institute (NCI), Bethesda, MD, applying the standard protocol of the NCI^19^.

### Antiproliferative assay against A375 human melanoma cell line

It was done as reported in our previous reports^17^^,^^20–22^.

### Kinase profiling

Reaction Biology Corp. Kinase HotSpotSM service was used for screening of compounds **1i**, **1j**, and **1l** according to the reported assay protocol^23^.

## Results and discussion

### Chemistry

**Scheme 1.**
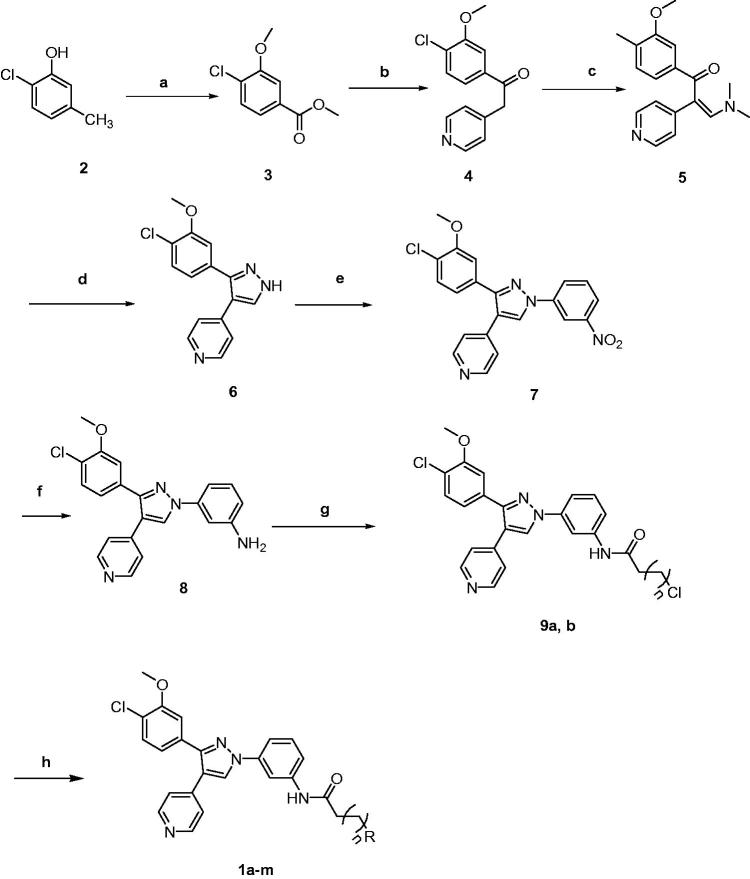
Reagents and conditions: (a) i) (CH_3_)_2_SO_4_, K_2_CO_3_, acetone, reflux, 1 h; ii) KMnO4, C_5_H_5_N, H_2_O, 50 °C, 24 h, then rt, 13 h; iii) acetyl chloride, CH_3_OH, rt, 15 h; (b) 4-picoline, LiHMDS, THF, rt, overnight; (c) DMF-DMA, rt, 18 h; (d) hydrazine monohydrate, C_2_H_5_OH, rt, overnight; (e) 1-iodo-4-nitrobenzene, K_2_CO_3_, CuI, L-proline, DMSO, 90 °C, 8 h; (f) H_2_, Pd/C, THF, rt, 2 h; (g) chloroacetyl chloride, or chloropropionyl chloride, TEA, CH_2_Cl_2_, −10 °C, 15 min; (h) appropriate amine derivative, TEA, CH_2_Cl_2_, rt, 1 h.

Synthesis of the designed compounds **1a–m** was carried out using the reactions adopted in Scheme 1. The phenolic starting material **2** was methylated using dimethyl sulfate in the presence of potassium carbonate to yield the methoxy analogue, which was further oxidised using potassium carbonate to 3-methoxy-4-chlorobenzoic acid. Esterification of the 3-methoxy-4-chlorobenzoic acid with methanol in the presence of acetyl chloride gave the corresponding methyl ester **3**. Activation of the ester with strong base as lithium bis(trimethylsilyl)amide (LiHMDS) then dropwise addition of 4-picoline produced the pyridyl intermediate **4**. Cyclisation to the pyrazole compound **6** was carried out by treatment of **4** with dimethylformamide dimethyl acetal (DMF-DMA) to produce compound **5**, and subsequent addition of hydrazine monohydrate. Heating of 3,4-driarylpyazole compound **6** with 3-iodonitrobenzene at 90 °C in DMSO to get the 3-nitrophenyl derivative **7**. The nitro derivative **7** underwent reduction with Pd/C giving the amine compound **8**. Compound **8** was reacted with either chloroacetyl chloride or chloropropionyl chloride giving chloroacetamide or chloropropionamide derivatives **9a,b**, respectively. The reaction of the terminal alkyl halide moiety of compounds **9a,b** with substituted or unsubstituted aliphatic cyclic amines yielded the target compounds **1a–m**. Structures of the target compounds and their yield percentages are illustrated in Table 1.

**Table 1.**
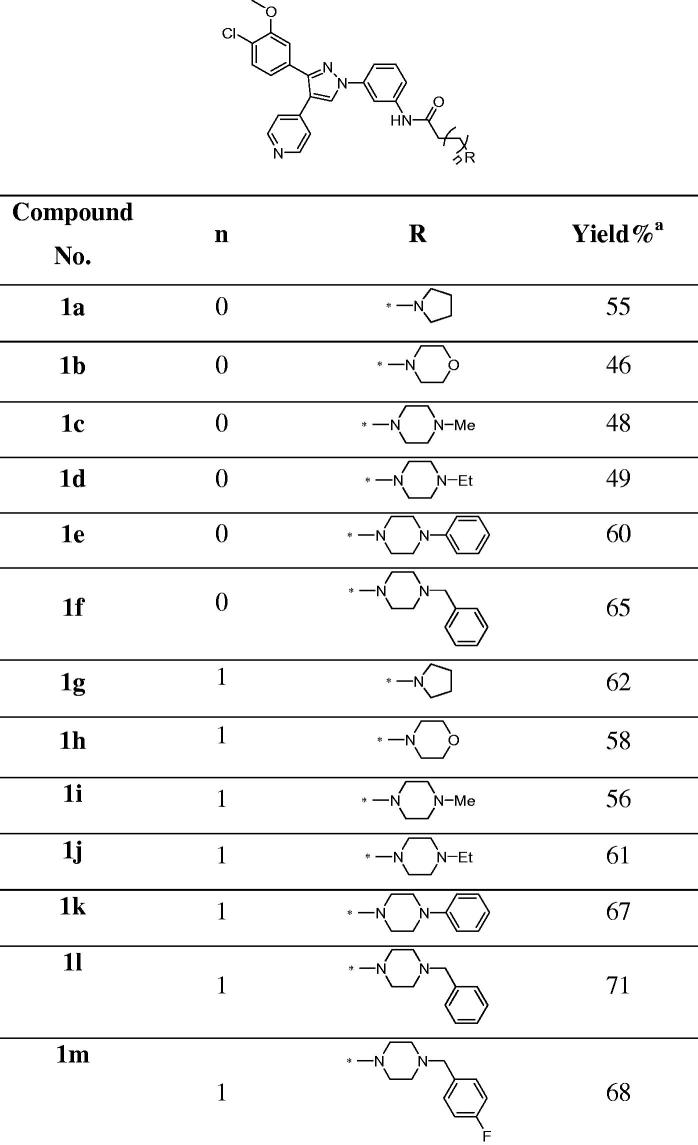
Structures of the target amide compounds **1a–m** and their yield percent.

a Yield of the last step.

### *In vitro* antiproliferative activity

#### Single-dose testing

**Figure 2.**
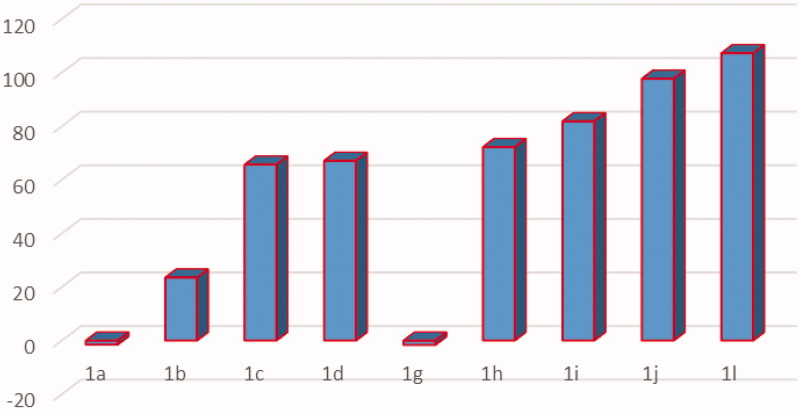
Mean percentage inhibition of target compounds against NCI-58 cancer cell line panel.

The designed compounds’ structures were submitted to the National Cancer Institute (NCI), Bethesda, MD, and nine of them were chosen for testing their antineoplastic activity. The selected compounds shown in Figure 2 were *in vitro* tested over 58 cancer cell lines of nine different tissues (blood, lung, colon, CNS, skin, ovary, kidney, prostate, and breast). They were examined at a single-dose concentration of 10 μM, for detecting growth inhibition percentages over the 58 tested cell lines. Figure 2 illustrates the mean percentage inhibition of each tested compound against the NCI-58 cancer cell line panel. The NCI results as well as representative ^1^H NMR, ^13^C NMR, and MS spectra are depicted in the Supplementary File.

The outcome data expressed that the 1,3,4-triarylpyrazole derivatives possessing ethylene spacer (compounds **1g–j** and **1l**) showed better activity than those derivatives with methylene spacer in compounds **1a–d**. They also exhibited higher mean % inhibition values than those of methylene. This could be attributed to better compound fitting and stronger affinity at the receptor site, or by even increasing its lipophilicity. These effects would enable optimal drug interaction in the enzyme active site, and therefore better antiproliferative activity.

**Figure 3.**
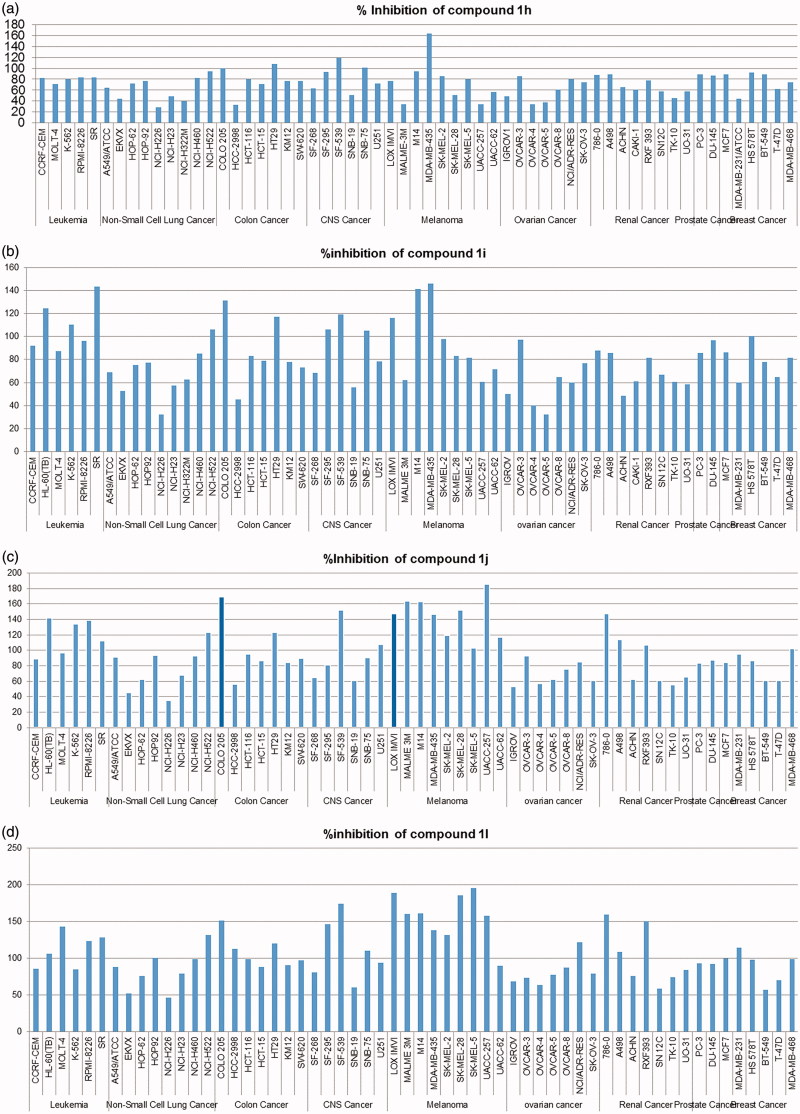
Inhibitory effects of compounds **1h–j** and **1l** against the NCI-58 cancer cell line panel. (a) **1h**, (b) **1i**, (c) **1j**, and (d) **1l**.

Regarding the effect of terminal moiety on the activity, it was found that substitution with morpholine (**1b**,**h**) achieved better mean % inhibition than that of piperidinyl derivatives (**1a**,**g**). This may be due to the presence of the oxygen atom making more hydrogen bond or enhancing the hydrophilic/aqueous solubility properties hence more compound fitting inside the receptor. It was also found that replacing morpholine with *N-*substituted piperazinyl derivatives as methyl, ethyl, phenyl, and benzyl with both shorter and longer spacer had made great enhancement of activity which may be caused by nitrogen atom instead of oxygen and protrusion of the substituent group; methyl, ethyl, phenyl, or benzyl exhibited higher mean percentage inhibition than those analogues without protrusion with lipophilic moiety leading to either more fitting of the compound within the receptor site or through enhancing its lipophilicity resulting in extra cell perforation and so, better cytotoxic activity (for methylene linker compounds **1c,d** gave higher mean percentage inhibition than compounds **1a**,**b**) and (for ethylene linker compounds **1i–m** gave higher mean percentage inhibition than **1g** and **1h**. So it could be concluded that ethylene spacer and substituted piperazine derivatives at the terminal position of the aminophenyl ring of the pyrazole are optimum for activity. As expressed in Figure 2, we can see compound **1l** exhibited 107.21% mean inhibition percentage at 10 μM concentration and compounds as **1h–j** showed 72, 81, and 97%, respectively, as mean percentage inhibition. Moreover, they exerted greater than 100% inhibition over many cancer cell lines. For example, compound **1j** exhibited 185.46, 169.25, and 163.6% against UACC-257, COLO 205, and MALME-3M cancer cell lines, respectively. Other compounds have also achieved broad-spectrum activity and better inhibition values of more than 100% inhibition over various cancer cell lines as shown in Figure 3.

#### Five dose testing

Compounds **1h–j** and **1l** that have shown encouraging results in single-dose testing were further selected for a five-dose testing mode, to detect their IC_50_ values over 58 different cancer cells. Table 2 illustrates the mean IC_50_ values of these 4 compounds over the nine cancer types.

**Table 2.** Mean IC_50_ values (μM) of the tested compounds over *in vitro* subpanel cancer cell lines^a^.

Subpanel cancer line^b^									
	I	II	III	IV	V	VI	VII	VIII	IX
No. of cell lines in each subpanel	5	8	7	6	9	7	8	2	6
**1h**	2.25	3.12	2.35	2.48	1.44	2.81	2.97	2.11	2.25
**1i**	**1.15**	**1.67**	**1.00**	**1.29**	**0.94**	**2.41**	**1.67**	**0.99**	2.32
**1j**	**0.41**	**0.85**	**0.54**	**0.55**	**0.71**	**1.16**	**0.80**	**0.44**	**0.74**
**1l**	**0.72**	**1.48**	**0.68**	**1.58**	**1.03**	**2.02**	**1.51**	**1.13**	**2.06**
**Sorafenib**	2.43	2.25	2.19	2.33	1.87	2.88	2.94	2.58	2.17

a Mean IC_50_ values were calculated by dividing the summation of IC_50_ values of the compound over cell lines of the same cancer type by the number of cell lines in the subpanel. ^b^I: Leukemia; II: non-small cell lung cancer; III: colon cancer; IV: CNS Cancer; V: melanoma VII: ovarian cancer; VII: renal cancer; VIII: prostate cancer; IX: breast cancer. Bold figures indicate superior potency than the reference drug, Sorafenib.

The tested compounds exerted high activity with sub-micromolar to one-digit micromolar IC_50_ scale against all the nine cancer subpanels. Compounds **1i**,**j**, and **1l** exerted superior potencies compared with sorafenib (Table 2). Table 3 illustrates the 4 tested compounds’ IC_50_ data in five-dose testing mode over the most perceptive cell line of each subpanel. Compounds **1h–j** and **1l** exerted superior potencies against the most perceptive cell line of each cancer subpanel as contrasted with sorafenib. Most of their IC_50_ values were in the sub-micromolar range. Among them, compound **1j** possessing ethylene spacer and *N*-ethylpiperazinyl terminal moiety was the most potent.

**Table 3.** IC_50_ values (μM) of the tested compounds over the most sensitive cell line of each subpanel.

Cancer cell line											
Compound no.	SR^a^	HOP-92^b^	NCI-H522^b^	HCT-29^c^	SW-620^c^	SNB-75^d^	SK-MEL-5^e^	OVCAR-3^f^	UO-31^g^	PC-3^h^	MDA-MB-468^i^
**1h**	**0.68**	**2.06**	**0.44**	**1.90**	**1.56**	**0.82**	**0.62**	**0.64**	**1.69**	**1.74**	**0.63**
**1i**	**0.47**	**0.67**	**0.28**	**0.37**	**0.44**	**0.59**	**0.53**	**0.36**	**1.38**	**1.00**	**0.35**
**1j**	**0.43**	**0.78**	**0.27**	**0.38**	**0.34**	**0.39**	**0.51**	**0.35**	**0.92**	**0.45**	**0.27**
**1l**	**0.39**	**0.79**	**0.36**	**0.32**	**0.49**	**1.55**	**1.10**	**0.39**	**0.89**	**0.71**	**0.39**
**Sorafenib**	3.16	1.58	1.99	1.99	2.51	3.16	1.58	2.51	2.51	1.99	1.99

a Leukemia cell line; ^b^non-small cell lung cancer cell line; ^c^colon cancer cell line; ^d^CNS cancer cell line; ^e^melanoma cell line; ^f^ovarian cancer cell line; ^g^renal cancer cell line; ^h^prostate cancer cell line; ^i^breast cancer cell line. Bold figures indicate superior potency than the reference compound, Sorafenib.

### A375 melanoma cell line screening

In addition to the 58 cancer cell lines, 10 of our target compounds that were promising were tested also against A375 human melanoma cell line. The IC_50_ values are summarised in Table 4. The most potent compound of the previous series with *para* substitution was also reported for comparison^20^. Sorafenib was used as a reference standard. From Table 4, we can see that compounds exerted better activity against sorafenib. They all exhibited lower IC_50_ values than that of sorafenib. Compounds with ethylene spacer (compounds **1h–m**) were more active than those with methylene linker (compounds **1b–d** and **1f**). It was also found that substituted piperazinyl derivatives with both methylene and ethylene spacer compound **1c–g** and **1i–m** were active than morpholino derivatives **1b**,**h**, respectively, which may be attributed to presence of aliphatic or aromatic substitution on piperazinyl moiety that may make more fitting in the receptor through lipophilic interaction within it or even add to the compound’s lipophilicity so it may penetrate better through the cell reaching its molecular target(s). We have also found that compounds with *N-*methyl, ethyl, or benzyl piperazinyl moieties showed better IC_50_ on A375 melanoma cell line than that of morpholino, phenyl, and fluorobenzyl piperazinyl moieties. Compound **1c**,**d** with methylene spacer are more potent than **1f**,**b** and compounds **1i,j,** and **1l** with ethylene spacer are more potent than compounds **1k**,**m**. It was also found that the most potent compound of *meta* series **1j** exerted higher activity with IC_50_ value of 0.82 μM relative to that the most potent one of *para* series with IC_50_ 1.82 μM against A375 human melanoma cancer cell line.

**Table 4.** IC_50_ values of the tested compounds against A375 human melanoma cell line.

Compound no.	IC_50_ (µM)^a^
**1b**	4.46 ± 0.35
**1c**	4.24 ± 0.25
**1d**	4.02 ± 0.26
**1f**	4.37 ± 0.36
**1h**	2.62 ± 1.21
**1i**	0.96 ± 0.13
**1j**	0.82 ± 0.15
**1k**	1.64 ± 0.23
**1l**	1.24 ± 0.38
**1m**	1.66 ± 0.31
^b^	1.82 ± 0.13
Sorafenib	5.60 ± 0.52

a The IC_50_ values are expressed as means of triplicate assays ± SEM. ^b^The most potent compound of *para* series.

### *In vitro* kinase screening

**Figure 4.**
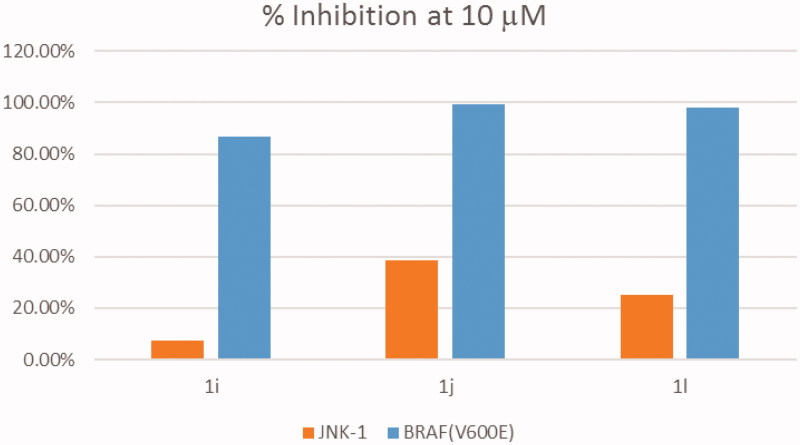
Inhibitory effect of compounds **1i**, **1j**, and **1l** on JNK-1 and V600E-B-RAF kinases.

In order to investigate the mechanism of action of these target compounds at molecular level that showed promising results against the cancer cell lines, the most potent compounds **1i**, **1j**, and **1l** were chosen to be examined at a single-dose concentration of 10 µM over two types of kinases V600E-B-RAF which is over-expressed in A375^20^ and some other cell lines from NCI panel such as SK-MEL-5, and JNK kinases which is not overexpressed in such cell lines trying to deduce their possible mechanism of action. As shown in Figure 4, the three compounds showed stronger inhibitory effect over V600E-B-RAF kinase than JNK-1 kinase at 10 µM. Compound **1j** exhibited almost 100% inhibition against V600E-B-RAF kinase. The two other compounds gave 86.57 and 97.92% against V600E-B-RAF, but 7.57 and 25.04% against JNK-1 kinase. This indicates that there is about 3 folds more selectivity against V600E-B-RAF kinase than that corresponding to JNK-1 kinase. Compound **1j** showed high activity over several cancer cell lines in which V600E-B-RAF is over-expressed such as COLO 205, HT 29 colon cancer cell lines, and SK-MEL-5 melanoma cell line^20^. As explained previously, V600E-B-RAF is over-expressed in diverse cancer types. So we can deduce from the inhibitory effect of compound **1j** on V600E-B-RAF kinases is, at least in part, considered as a possible mechanism of its antiproliferative effect.

## Conclusions

In this investigation, a series of new 1,3,4-triarylpyrazole analogues having morpholino and substituted piperazinyl terminal moieties were synthesised. Out of the thirteen submitted compounds, nine were examined at single dose *in vitro* antiproliferative test over NCI-58 cancer cell line panel of nine different cancer tissues, and four promising compounds were selected for five dose testing mode. The compounds were also tested against A375 human melanoma cell line. These four compounds **1h–j** and **1l** with ethylene linker and *N-*substituted piperazinyl showed higher mean percentage inhibition of 72.18, 81.8, 97.68, and 107.21%, respectively, and compound **1j** possessing *N*-ethylpiperazinyl moiety attached to 1,3,4-triarylpyrazole ring through ethylene linker showed the most promising results at five-dose testing. It was also found that many compounds with *meta* substitution of this nucleus has exerted higher activity against A375 than that of *para* series. It showed elevated potency and broad-spectrum antiproliferative activities over several different cell lines of different cancer types higher than sorafenib. It has also exerted tremendous percentage inhibition against V600E mutant B-RAF kinase of 99.17%. So *N*-substituted piperazinyl moiety, ethylene spacer, and *meta*-disubstitued phenyl at *N1* of the core pyrazole ring are optimal for activity of these compounds. They could express antiproliferative activity through inhibition of V600E-B-RAF. Further modifications of this series in order to optimise the scaffold and to improve their anticancer activities are in progress.

## Supplementary Material

Supplemental Material

## References

[1] World Health Organization, Media Centre, Cancer. Available from: http://www.who.int/mediacentre/factsheets/fs297/en/ [last accessed 16 March 2019].

[2] Belpomme D, Irigaray P, Sasco AJ, et al. The growing incidence of cancer: role of lifestyle and screening detection. Int J Oncol 2007;30:1037–49. 17390005 10.3892/ijo.30.5.1037

[3] Frankish H 15 million new cancer cases per year by 2020, says WHO. Lancet 2003;361:1278. 12699963 10.1016/S0140-6736(03)13038-3

[4] Anderson CM, Buzaid AC, Legha SS Systemic treatments for advanced cutaneous melanoma. Oncology (Williston Park) 1995;9:1149–58. 8703684

[5] Smith RA, Dumas J, Adnane L, et al. Recent advances in the research and development of RAF kinase inhibitors. Curr Top Med Chem 2006;6:1071–89. 16842147 10.2174/156802606777812077

[6] Brose MS, Volpe P, Feldman M, et al. BRAF and RAS mutations in human lung cancer and melanoma. Cancer Res 2002;62:6997–7000. 12460918

[7] Wilhelm SM, Carter C, Tang L, et al. BAY 43-9006 exhibits broad spectrum oral antitumor activity and targets the RAF/MEK/ERK pathway and receptor tyrosine kinases involved in tumor progression and angiogenesis. Cancer Res 2004;64:7099–109. 15466206 10.1158/0008-5472.CAN-04-1443

[8] El-Gamal MI, Abdel-Maksoud MS, Gamal El-Din MM, et al. Cell-based biological evaluation of a new bisamide FMS kinase inhibitor possessing pyrrolo[3,2-*c*]pyridine scaffold. Arch Pharm Chem Life Sci 2014;347:635–41. 10.1002/ardp.201400051 24942978

[9] Khan MA, El-Gamal MI, Abdel-Maksoud MS, et al. Broad-spectrum antiproliferative activity of diarylureas and diarylamides possessing pyrrolo[3,2-*c*]pyridine scaffold. J Pharm Pharmacol 2014;2:157–69.

[10] El-Gamal MI, Khan MA, Abdel-Maksoud MS, et al. A new series of diarylamides possessing quinoline nucleus: synthesis, in vitro anticancer activities, and kinase inhibitory effect. Eur J Med Chem 2014;87:484–92. 25282271 10.1016/j.ejmech.2014.09.068

[11] Gamal El-Din MM, El-Gamal MI, Abdel-Maksoud MS, et al. Synthesis and broad-spectrum antiproliferative activity of diarylamides and diarylureas possessing 1,3,4-oxadiazole derivatives. Bioorg Med Chem Lett 2015;25:1692–9. 25801936 10.1016/j.bmcl.2015.03.001

[12] Capdeville R, Buchdunger E, Zimmermann J, et al. Glivec (STI571, imatinib), a rationally developed, targeted anticancer drug. Nat Rev Drug Discov 2002;1:493–502. 12120256 10.1038/nrd839

[13] Shawver LK, Slamon D, Ullrich A Smart drugs: tyrosine kinase inhibitors in cancer therapy. Cancer Cell 2002;1:117–23. 12086869 10.1016/s1535-6108(02)00039-9

[14] Choi WK, Oh CH Synthesis and antiproliferative activities of 1-substituted-3-(3-chloro-5-methoxyphenyl)-4-pyridinylpyrazole derivatives against melanoma cell line. Bull Korean Chem Soc 2009;30:2027–31.

[15] Choi WK, El-Gamal MI, Choi WK, et al. New diarylureas and diarylamides containing 1,3,4-triarylpyrazole scaffold: synthesis, antiproliferative evaluation against melanoma cell lines, ERK kinase inhibition, and molecular docking studies. Eur J Med Chem 2011;46:5754–62. 22014559 10.1016/j.ejmech.2011.08.013

[16] El-Gamal MI, Park YS, Chi DY, et al. New triarylpyrazoles as broad-spectrum anticancer agents: design, synthesis, and biological evaluation. Eur J Med Chem 2013;65:315–22. 23732996 10.1016/j.ejmech.2013.04.067

[17] El-Gamal MI, Choi HS, Cho HG, et al. Design, synthesis, and antiproliferative activity of 3,4-diarylpyrazole-1-carboxamide derivatives against, melanoma cell line. Arch Pharm Chem Life Sci 2011;344:745–54. 10.1002/ardp.201000375 21954060

[18] El-Gamal MI, Choi HS, Yoo KH, et al. Antiproliferative diarylpyrazole derivatives as dual inhibitors of the ERK pathway and COX-2. Chem Biol Drug Des 2013;82:336–47. 23834707 10.1111/cbdd.12186

[19] DTP Human Tumor Cell Line Screen Process: Available from: http://www.dtp.nci.nih.gov/branches/btb/ivclsp.html [last accessed 16 March 2019].

[20] Khan MA, El-Gamal MI, Tarazi H, et al. Design and synthesis of a new series of highly potent RAF kinase-inhibiting triarylpyrazole derivatives possessing antiproliferative activity against melanoma. Future Med Chem 2016;8:2197–211. 27845592 10.4155/fmc-2016-0057

[21] El-Gamal MI, Sim TB, Hong JH, et al. Synthesis of 1*H*-pyrazole-1-carboxamide derivatives and their antiproliferative activity against melanoma cell line. Arch Pharm Chem Life Sci 2011;344:197–204. 10.1002/ardp.201000057 21384419

[22] El-Gamal MI, Oh CH Design and synthesis of 3-(3-chloro-4-substituted phenyl)-4-(pyridin-4-yl)-1Hpyrazole-1-carboxamide derivatives and their antiproliferative activity against melanoma cell line. Bull Korean Chem Soc 2011;32:821–8.

[23] Reaction Biology + ProQinase = Your Partner for Drug Discovery Available from http://www.reactionbiology.com [last accessed 2 August 2019].

