# Supplementary material for: Design, synthesis, in vitro potent antiproliferative activity, and kinase inhibitory effects of new triarylpyrazole derivatives possessing different heterocycle terminal moieties
Source: J Enzyme Inhib Med Chem. 2019 Aug 27;34(1):1534–43. doi: 10.1080/14756366.2019.1653292 (PMC6720312; doi:10.1080/14756366.2019.1653292)

Supplementary Materials

NCI data (Single dose test mode) 2-9

NCI data(Five dose test mode) ……………………...……….……………......... 10-13

Representative ^{1}H NMR, ^{13}C NMR, and MS spectra ………………………..… 14-37

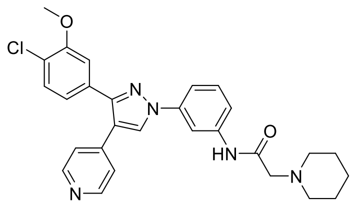

Compound 1a

Panel/Cell Line	Growth Percent
Leukemia	
CCRF-CEM	107.80
HL-60(TB)	107.27
K-562	119.74
MOLT-4	106.39
RPMI-8226	100.33
SR	104.59
Non-Small Cell Lung Cancer	
A549/ATCC	100.47
EKVX	97.06
HOP-62	95.15
HOP-92	109.79
NCI-H226	108.14
NCI-H23	97.89
NCI-H322M	106.43
NCI-H460	101.95
NCI-H522	85.68
Colon Cancer	
COLO 205	107.07
HCC-2998	99.38
HCT-116	95.32
HCT-15	103.01
HT29	88.69
KM12	91.19
SW-620	95.54
CNS Cancer	
SF-268	96.15
SF-295	102.73
SF-539	104.70
SNB-19	106.11
SNB-75	88.45
U251	112.27
Melanoma	
LOX IMVI	97.43
MALME-3M	100.04
M14	103.20
MDA-MB-435	102.62
SK-MEL-2	105.89
SK-MEL-28	109.38
SK-MEL-5	104.87
UACC-257	106.99
UACC-62	109.77
Ovarian Cancer	
IGROV1	105.21
OVCAR-3	98.06
OVCAR-4	103.48
OVCAR-5	92.82
OVCAR-8	112.73
NCI/ADR-RES	95.64
SK-OV-3	97.88
Renal Cancer	
786-0	94.53
A498	106.12
ACHN	95.70
CAKI-1	97.41
RXF 393	100.38
SN12C	100.88
TK-10	107.78
UO-31	79.06
Prostate Cancer	
PC-3	99.13
DU-145	105.06
Breast Cancer	
MCF7	99.08
MDA-MB-231/ATCC	112.07
HS 578T	106.53
BT-549	97.49
T-47D	114.56
MDA-MB-468	90.91
Mean	101.53
Delta	22.47
Range	40.68

Mean Growth Percent - Growth Percent

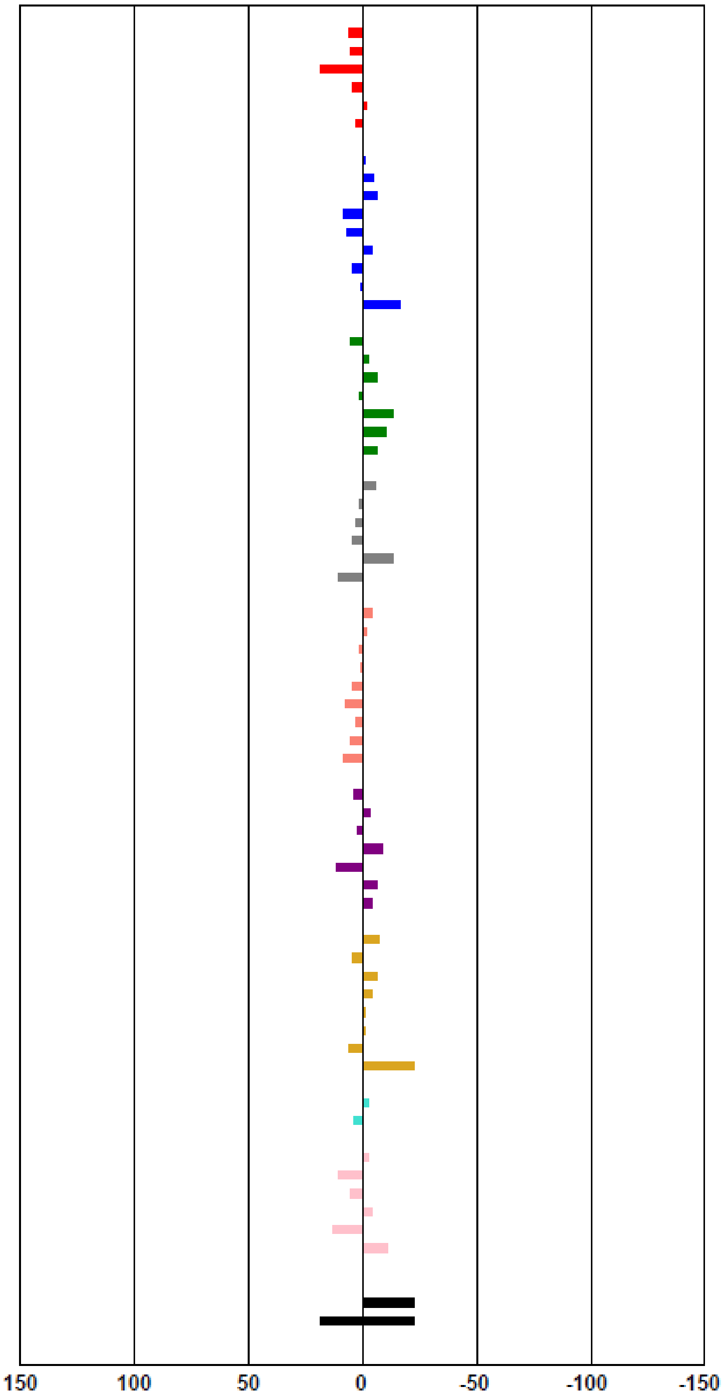

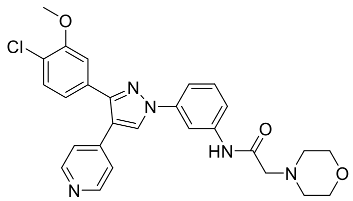

Compound 1b

Panel/Cell Line	Growth Percent
Leukemia	
CCRF-CEM	70.86
HL-60(TB)	83.99
K-562	42.17
MOLT-4	74.29
RPMI-8226	60.48
SR	37.28
Non-Small Cell Lung Cancer	
A549/ATCC	83.82
EKVX	95.80
HOP-62	98.89
HOP-92	50.35
NCI-H226	82.22
NCI-H23	90.85
NCI-H460	84.04
NCI-H522	49.09
Colon Cancer	
COLO 205	88.26
HCC-2998	112.05
HCT-116	82.73
HCT-15	66.70
HT29	65.22
KM12	71.93
SW-620	62.18
CNS Cancer	
SF-268	85.39
SF-295	76.82
SF-539	86.95
SNB-19	88.02
SNB-75	65.50
U251	92.74
Melanoma	
LOX IMVI	63.26
MALME-3M	80.50
M14	60.98
MDA-MB-435	6.77
SK-MEL-2	82.09
SK-MEL-28	84.32
SK-MEL-5	48.18
UACC-257	93.90
UACC-62	61.42
Ovarian Cancer	
IGROV1	79.59
OVCAR-3	77.23
OVCAR-4	100.99
OVCAR-5	107.66
OVCAR-8	76.42
NCI/ADR-RES	59.02
SK-OV-3	95.83
Renal Cancer	
786-0	94.61
A498	93.26
ACHN	87.60
RXF 393	85.08
SN12C	85.38
TK-10	124.84
UO-31	68.58
Prostate Cancer	
PC-3	58.02
DU-145	82.29
Breast Cancer	
MCF7	62.38
MDA-MB-231/ATCC	83.09
HS 578T	76.27
BT-549	62.25
T-47D	72.20
MDA-MB-468	74.94
Mean	76.51
Delta	69.74
Range	118.07

Mean Growth Percent - Growth Percent

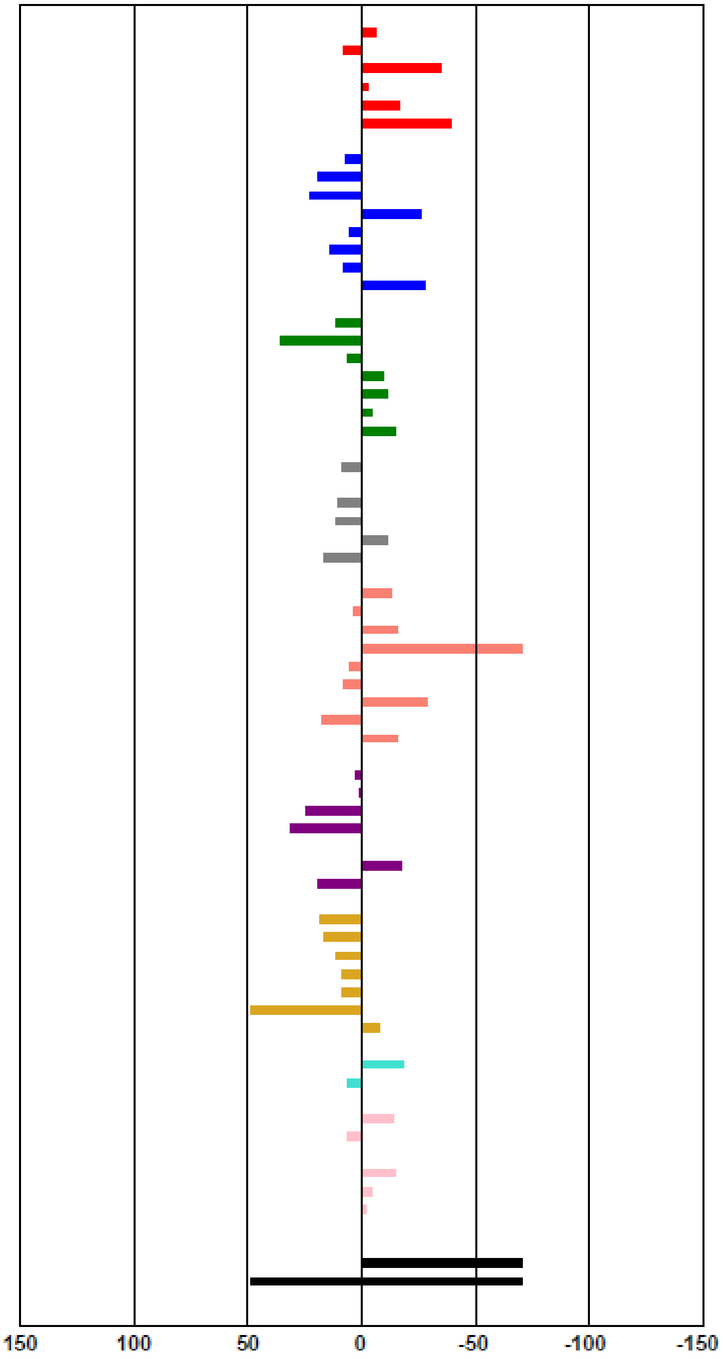

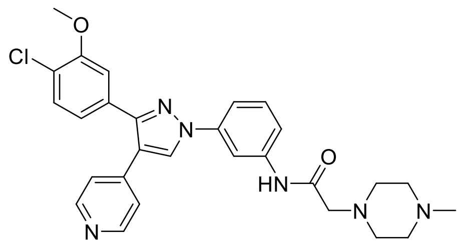

Compound 1c

Panel/Cell Line	Growth Percent
Leukemia	
CCRF-CEM	23.89
HL-60(TB)	5.27
K-562	11.78
MOLT-4	26.37
RPMI-8226	15.94
SR	5.84
Non-Small Cell Lung Cancer	
A549/ATCC	29.27
EKVX	63.60
HOP-62	58.60
HOP-92	15.44
NCI-H226	74.28
NCI-H23	50.43
NCI-H460	13.33
NCI-H522	18.15
Colon Cancer	
COLO 205	13.51
HCC-2998	85.63
HCT-116	17.35
HCT-15	40.47
HT29	4.08
KM12	26.12
SW-620	20.79
CNS Cancer	
SF-268	47.72
SF-295	21.77
SF-539	0.85
SNB-19	55.54
SNB-75	30.46
U251	30.82
Melanoma	
LOX IMVI	14.62
MALME-3M	46.35
M14	8.05
MDA-MB-435	-47.55
SK-MEL-2	28.23
SK-MEL-28	60.75
SK-MEL-5	27.35
UACC-257	24.88
UACC-62	48.35
Ovarian Cancer	
IGROV1	57.54
OVCAR-3	12.81
OVCAR-4	59.84
OVCAR-5	68.19
OVCAR-8	57.16
NCI/ADR-RES	42.22
SK-OV-3	55.94
Renal Cancer	
786-0	33.65
A498	35.61
ACHN	44.72
RXF 393	35.03
SN12C	64.85
TK-10	102.91
UO-31	50.16
Prostate Cancer	
PC-3	26.61
DU-145	19.40
Breast Cancer	
MCF7	15.22
MDA-MB-231/ATCC	46.12
HS 578T	38.19
BT-549	36.58
T-47D	61.32
MDA-MB-468	10.68
Mean	34.36
Delta	81.91
Range	150.46

Mean Growth Percent - Growth Percent

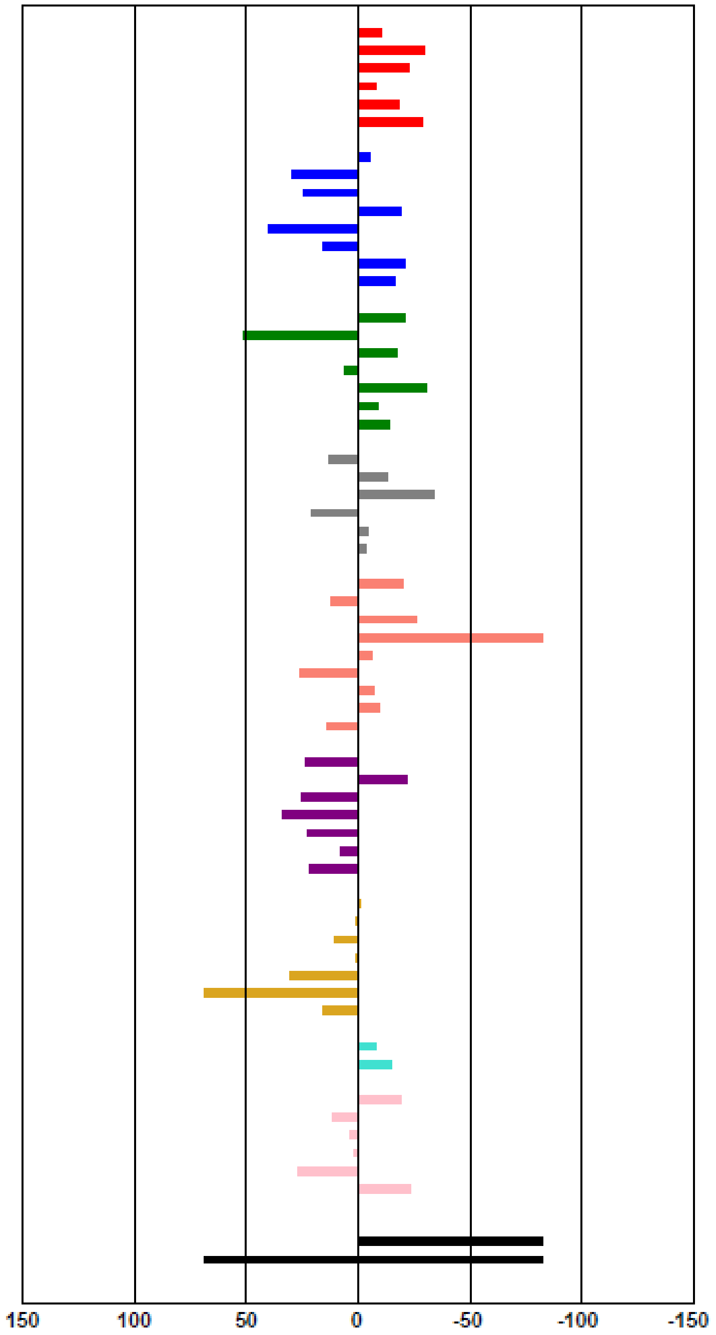

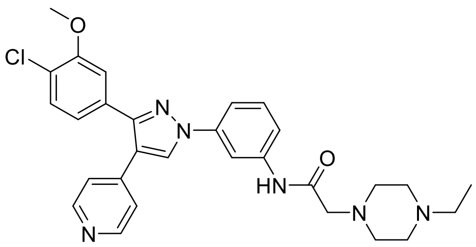

Compound 1d

Panel/Cell Line	Growth Percent
Leukemia	
CCRF-CEM	24.93
HL-60(TB)	5.71
K-562	12.76
MOLT-4	25.64
RPMI-8226	19.90
SR	5.50
Non-Small Cell Lung Cancer	
A549/ATCC	30.17
EKVX	74.06
HOP-62	53.71
HOP-92	27.92
NCI-H226	66.64
NCI-H23	46.42
NCI-H460	13.43
NCI-H522	16.89
Colon Cancer	
COLO 205	12.85
HCC-2998	82.20
HCT-116	19.09
HCT-15	39.34
HT29	4.91
KM12	31.22
SW-620	20.85
CNS Cancer	
SF-268	50.85
SF-295	19.27
SF-539	-4.71
SNB-19	55.02
SNB-75	24.54
U251	32.43
Melanoma	
LOX IMVI	15.67
MALME-3M	47.63
M14	12.33
MDA-MB-435	-37.94
SK-MEL-2	34.52
SK-MEL-28	57.68
SK-MEL-5	24.16
UACC-257	46.25
UACC-62	46.86
Ovarian Cancer	
IGROV1	55.81
OVCAR-3	14.37
OVCAR-4	55.98
OVCAR-5	67.70
OVCAR-8	54.77
NCI/ADR-RES	29.75
SK-OV-3	46.22
Renal Cancer	
786-0	32.20
A498	34.54
ACHN	45.50
RXF 393	29.98
SN12C	61.83
TK-10	103.46
UO-31	46.88
Prostate Cancer	
PC-3	29.28
DU-145	25.84
Breast Cancer	
MCF7	13.91
MDA-MB-231/ATCC	-72.04
HS 578T	43.14
BT-549	56.45
T-47D	62.90
MDA-MB-468	12.69
Mean	32.93
Delta	104.97
Range	175.50

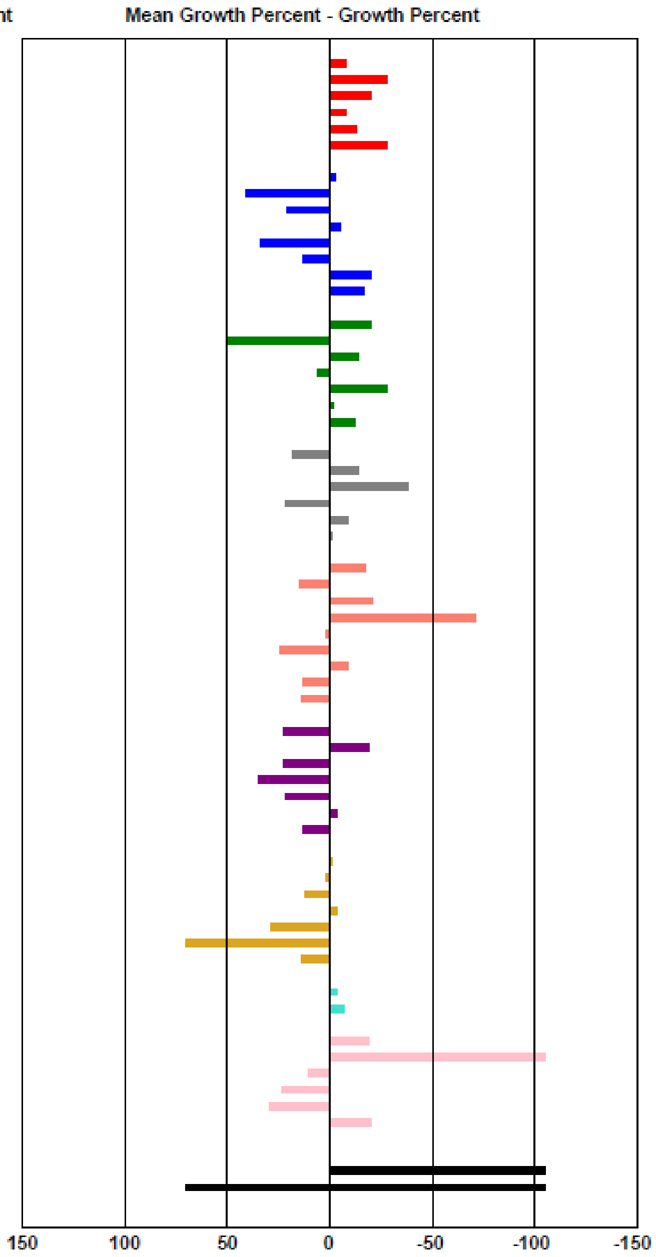

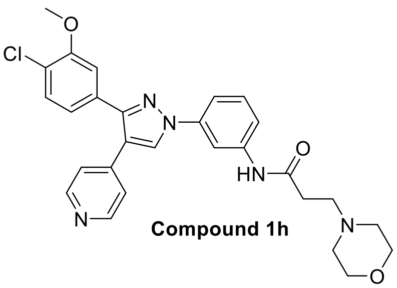

Compound 1h

Panel/Cell Line	Growth Percent
**Leukemia**	
CCRF-CEM	17.66
K-562	20.24
MOLT-4	28.59
RPMI-8226	17.14
SR	16.78
**Non-Small Cell Lung Cancer**	
A549/ATCC	35.79
EKVX	55.65
HOP-62	28.04
HOP-92	23.67
NCI-H226	71.00
NCI-H23	51.70
NCI-H322M	60.38
NCI-H460	17.85
NCI-H522	5.83
**Colon Cancer**	
COLO 205	0.42
HCC-2998	66.87
HCT-116	21.10
HCT-15	28.51
HT29	-7.51
KM12	23.72
SW-620	23.39
**CNS Cancer**	
SF-268	37.03
SF-295	6.52
SF-539	-19.67
SNB-19	48.82
SNB-75	-1.70
U251	27.74
**Melanoma**	
LOX IMVI	23.06
MALME-3M	66.27
M14	5.06
MDA-MB-435	-64.10
SK-MEL-2	14.79
SK-MEL-28	49.61
SK-MEL-5	20.32
UACC-257	66.18
UACC-62	43.08
**Ovarian Cancer**	
IGROV1	51.76
OVCAR-3	14.31
OVCAR-4	66.47
OVCAR-5	62.17
OVCAR-8	39.51
NCI/ADR-RES	21.51
SK-OV-3	25.10
**Renal Cancer**	
786-0	11.71
A498	10.91
ACHN	34.23
CAKI-1	38.64
RXF 393	22.16
SN12C	42.84
TK-10	54.46
UO-31	41.90
**Prostate Cancer**	
PC-3	11.44
DU-145	13.03
**Breast Cancer**	
MCF7	11.36
MDA-MB-231/ATCC	55.38
HS 578T	7.21
BT-549	11.64
T-47D	38.30
MDA-MB-468	25.25
Mean	27.82
Delta	91.92
Range	135.10

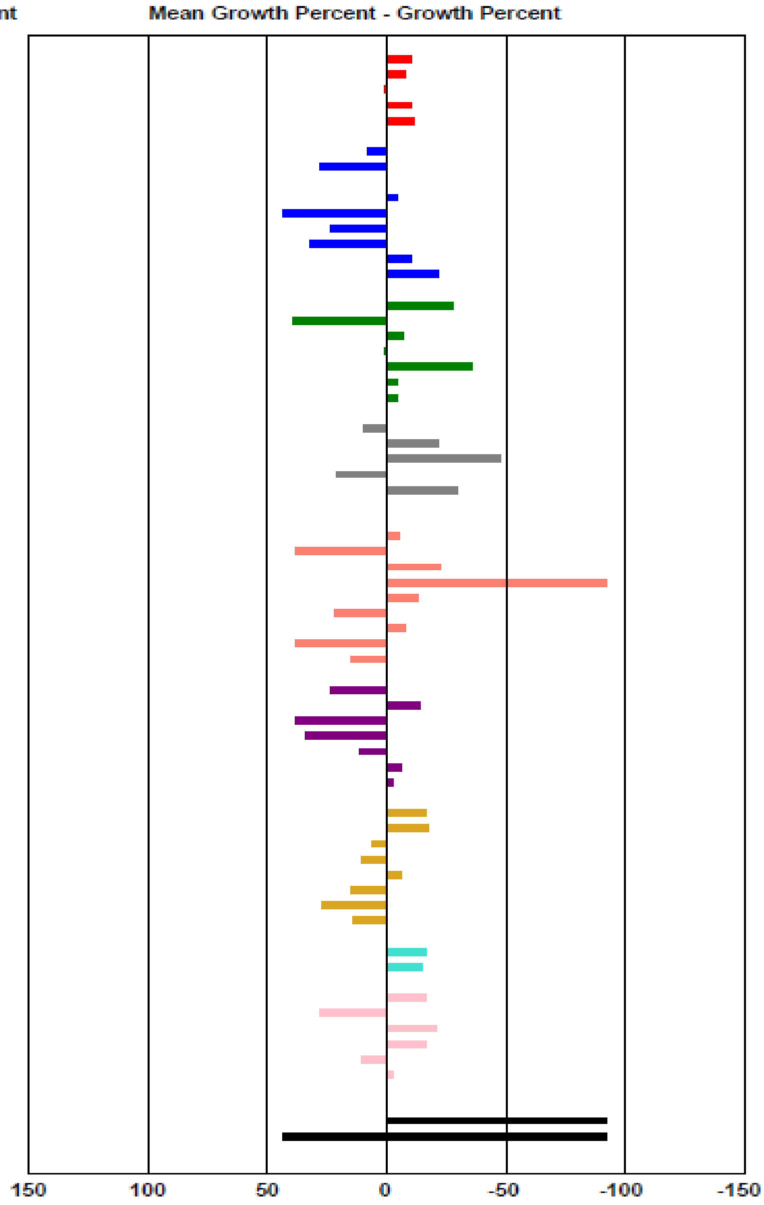

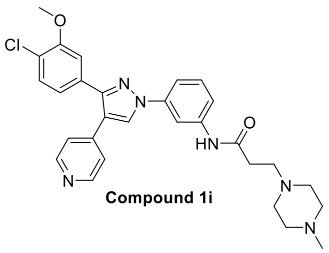

Compound 1i

Panel/Cell Line	Growth Percent
**Leukemia**	
CCRF-CEM	9.08
HL-60(TB)	-24.54
K-562	-10.40
MOLT-4	12.71
RPMI-8226	4.86
SR	-43.45
**Non-Small Cell Lung Cancer**	
A549/ATCC	30.67
EKVX	47.09
HOP-62	24.64
HOP-92	22.68
NCI-H226	67.70
NCI-H23	42.53
NCI-H322M	37.54
NCI-H460	14.85
NCI-H522	-6.10
**Colon Cancer**	
COLO 205	-31.64
HCC-2998	54.74
HCT-116	16.98
HCT-15	21.20
HT29	-17.37
KM12	22.24
SW-620	26.81
**CNS Cancer**	
SF-268	31.51
SF-295	-6.03
SF-539	-19.61
SNB-19	44.03
SNB-75	-5.41
U251	21.45
**Melanoma**	
LOX IMVI	-16.02
MALME-3M	38.01
M14	-41.57
MDA-MB-435	-46.31
SK-MEL-2	2.29
SK-MEL-28	16.95
SK-MEL-5	8.48
UACC-257	39.32
UACC-62	28.19
**Ovarian Cancer**	
IGROV1	49.68
OVCAR-3	2.81
OVCAR-4	59.71
OVCAR-5	67.89
OVCAR-8	36.09
NCI/ADR-RES	39.86
SK-OV-3	23.10
**Renal Cancer**	
786-0	12.22
A498	14.07
ACHN	51.74
CAKI-1	38.71
RXF 393	18.58
SN12C	33.28
TK-10	39.21
UO-31	41.46
**Prostate Cancer**	
PC-3	13.98
DU-145	3.19
**Breast Cancer**	
MCF7	13.79
MDA-MB-231/ATCC	39.91
HS 578T	-0.23
BT-549	22.35
T-47D	35.30
MDA-MB-468	18.21
Mean	18.20
Delta	64.51
Range	114.20

Mean Growth Percent - Growth Percent

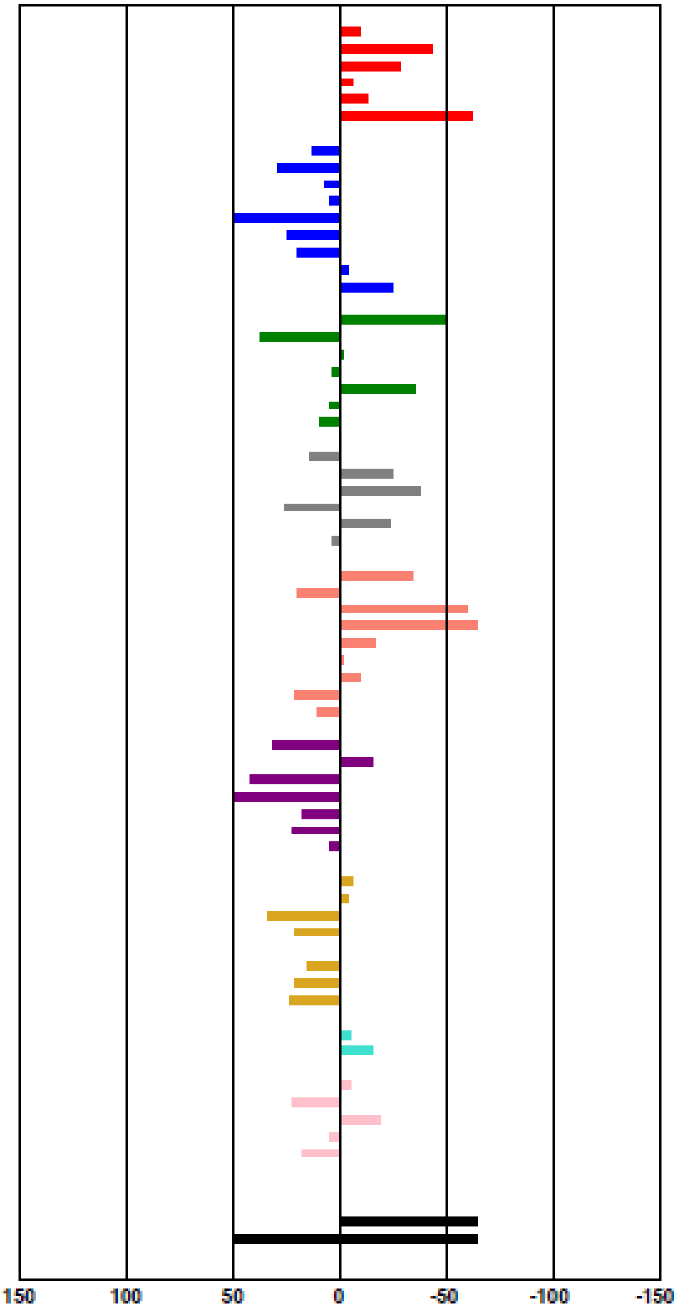

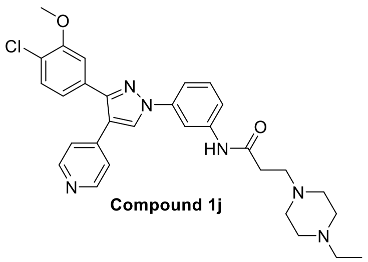

Compound 1j

Panel/Cell Line	Growth Percent
**Leukemia**	
CCRF-CEM	1.32
HL-60(TB)	-42.14
K-562	-33.98
MOLT-4	2.89
RPMI-8226	-38.77
SR	-12.30
**Non-Small Cell Lung Cancer**	
A549/ATCC	8.56
EKVX	54.50
HOP-62	37.68
HOP-92	6.07
NCI-H226	65.00
NCI-H23	32.22
NCI-H460	6.94
NCI-H522	-23.17
**Colon Cancer**	
COLO 205	-69.25
HCC-2998	43.85
HCT-116	5.22
HCT-15	13.68
HT29	-23.45
KM12	15.68
SW-620	10.25
**CNS Cancer**	
SF-268	35.38
SF-295	9.42
SF-539	-51.89
SNB-19	39.35
SNB-75	9.22
U251	-7.85
**Melanoma**	
LOX IMVI	-47.50
MALME-3M	-63.60
M14	-63.41
MDA-MB-435	-46.98
SK-MEL-2	-19.46
SK-MEL-28	-52.35
SK-MEL-5	-3.03
UACC-257	-85.46
UACC-62	-16.97
**Ovarian Cancer**	
IGROV1	47.20
OVCAR-3	7.41
OVCAR-4	43.06
OVCAR-5	37.65
OVCAR-8	24.22
NCI/ADR-RES	15.17
SK-OV-3	39.19
**Renal Cancer**	
786-0	-47.56
A498	-14.08
ACHN	37.25
RXF 393	-7.05
SN12C	38.81
TK-10	44.70
UO-31	34.60
**Prostate Cancer**	
PC-3	16.49
DU-145	12.37
**Breast Cancer**	
MCF7	15.45
MDA-MB-231/ATCC	4.87
HS 578T	13.14
BT-549	39.25
T-47D	38.77
MDA-MB-468	-2.30
Mean	2.32
Delta	87.78
Range	150.46

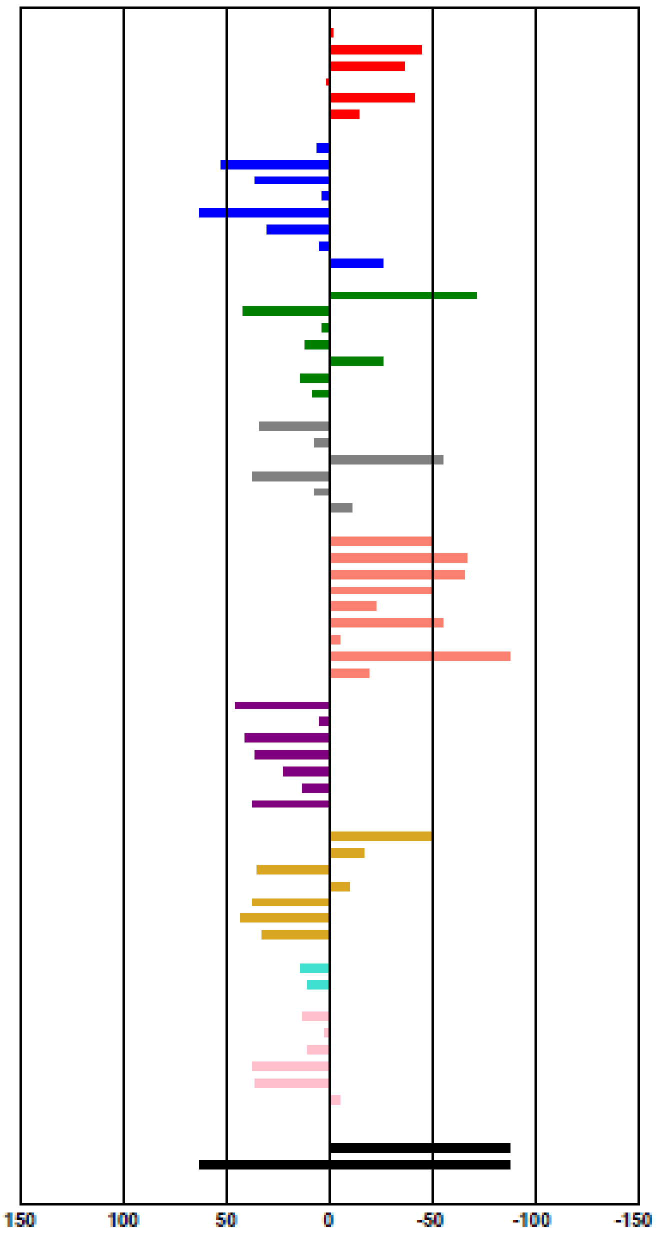

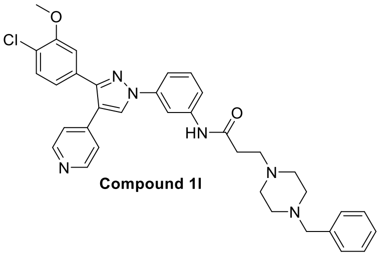

Compound 1l

Panel/Cell Line	Growth Percent
Leukemia	
CCRF-CEM	4.24
HL-60(TB)	-5.82
K-562	-43.07
MOLT-4	5.06
RPMI-8226	-23.23
SR	-28.47
Non-Small Cell Lung Cancer	
A549/ATCC	11.63
EKVX	48.15
HOP-62	23.99
HOP-92	-0.56
NCI-H226	54.00
NCI-H23	20.76
NCI-H460	1.48
NCI-H522	-31.38
Colon Cancer	
COLO 205	-50.87
HCC-2998	-12.77
HCT-116	1.30
HCT-15	11.84
HT29	-20.15
KM12	9.70
SW-620	2.77
CNS Cancer	
SF-268	19.10
SF-295	-46.54
SF-539	-74.27
SNB-19	39.92
SNB-75	-10.34
U251	6.53
Melanoma	
LOX IMVI	-88.64
MALME-3M	-60.68
M14	-61.26
MDA-MB-435	-38.33
SK-MEL-2	-31.19
SK-MEL-28	-85.39
SK-MEL-5	-95.26
UACC-257	-58.17
UACC-62	10.74
Ovarian Cancer	
IGROV1	31.88
OVCAR-3	7.04
OVCAR-4	36.20
OVCAR-5	22.91
OVCAR-8	12.65
NCI/ADR-RES	-21.39
SK-OV-3	22.32
Renal Cancer	
786-0	-59.85
A498	-8.39
ACHN	24.05
RXF 393	-50.60
SN12C	41.72
TK-10	26.05
UO-31	15.61
Prostate Cancer	
PC-3	7.06
DU-145	7.55
Breast Cancer	
MCF7	0.81
MDA-MB-231/ATCC	-14.66
HS 578T	2.23
BT-549	42.71
T-47D	29.66
MDA-MB-468	1.17
Mean	-7.21
Delta	88.05
Range	149.26

Mean Growth Percent - Growth Percent

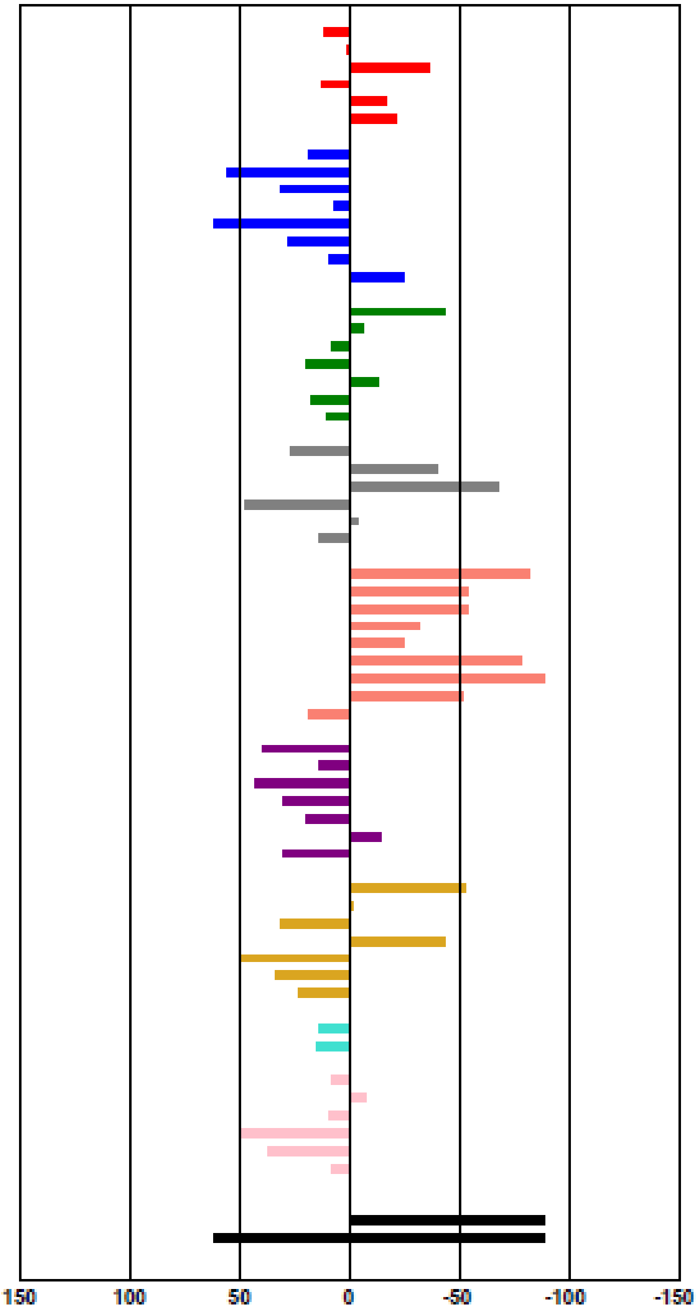

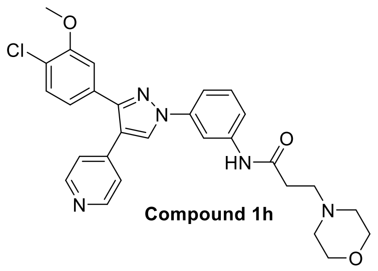

Compound 1h

Panel/Cell Line	Time Zero	Ctrl	Mean Optical Densities -8.0	-7.0	-6.0	-5.0	-4.0	Percent Growth -8.0	-7.0	-6.0	-5.0	-4.0	GI50	TGI	LC50
**Leukemia**															
CCRF-CEM	0.570	2.456	2.517	2.521	2.391	0.749	0.356	103	103	97	9	-38	3.43E-6	1.59E-5	> 1.00E-4
HL-60(TB)	0.799	2.476	2.396	2.384	2.342	0.638	0.381	95	94	92	-20	-52	2.37E-6	6.61E-6	8.47E-5
K-562	0.348	2.098	2.121	2.191	1.494	0.387	0.218	101	105	65	2	-37	1.76E-6	1.14E-5	> 1.00E-4
MOLT-4	0.712	2.740	2.743	2.687	2.406	0.798	0.348	100	97	84	4	-51	2.65E-6	1.19E-5	9.54E-5
RPMI-8226	0.772	2.671	2.723	2.749	2.433	0.841	0.643	103	104	87	4	-17	2.80E-6	1.51E-5	> 1.00E-4
SR	0.538	1.739	1.723	1.746	1.019	0.522	0.337	99	101	40	-3	-37	6.85E-7	8.53E-6	> 1.00E-4
**Non-Small Cell Lung Cancer**															
A549/ATCC	0.430	1.938	1.904	1.972	1.789	0.736	0.094	98	102	90	20	-78	3.75E-6	1.61E-5	5.17E-5
EKVX	0.756	2.457	2.234	2.287	2.084	1.231	0.287	87	90	78	28	-62	3.63E-6	2.04E-5	7.35E-5
HOP-62	0.608	1.367	1.234	1.237	1.133	0.715	0.033	83	83	69	14	-95	2.23E-6	1.35E-5	3.89E-5
HOP-92	1.682	2.285	2.222	2.258	2.081	1.772	0.212	90	96	66	15	-87	2.06E-6	1.40E-5	4.31E-5
NCI-H226	0.901	1.869	1.798	1.876	1.792	1.208	0.322	93	101	92	32	-64	4.98E-6	2.14E-5	7.09E-5
NCI-H23	0.746	2.468	2.420	2.312	2.108	1.071	0.363	97	91	79	19	-51	3.04E-6	1.86E-5	9.57E-5
NCI-H322M	0.771	2.170	2.060	2.002	1.994	1.257	0.040	92	88	87	35	-95	5.14E-6	1.85E-5	4.51E-5
NCI-H460	0.342	3.162	3.233	3.254	2.719	0.546	0.107	103	103	84	7	-69	2.79E-6	1.24E-5	5.65E-5
NCI-H522	0.979	2.248	2.148	2.121	1.342	0.877	0.238	92	90	29	-10	-76	4.48E-7	5.40E-6	4.04E-5
**Colon Cancer**															
COLO 205	0.476	1.783	1.703	1.766	1.479	0.262	0.084	94	99	77	-45	-82	1.66E-6	4.27E-6	1.36E-5
HCC-2998	0.862	3.042	3.018	3.033	2.992	1.416	0.156	99	100	98	25	-82	4.57E-6	1.73E-5	5.04E-5
HCT-116	0.405	2.891	2.852	2.787	2.622	0.605	0.036	98	96	89	8	-91	3.04E-6	1.20E-5	3.84E-5
HCT-15	0.275	2.256	2.083	2.122	1.360	0.330	0.054	91	93	55	3	-81	1.24E-6	1.08E-5	4.30E-5
HT29	0.301	1.761	1.811	1.793	1.296	0.349	0.078	103	102	68	3	-74	1.90E-6	1.10E-5	4.87E-5
KM12	0.802	3.190	3.164	3.152	2.678	0.930	0.121	99	98	79	5	-85	2.46E-6	1.15E-5	4.10E-5
SW-620	0.250	1.774	1.699	1.723	1.166	0.371	0.072	95	97	60	8	-71	1.56E-6	1.26E-5	5.40E-5
**CNS Cancer**															
SF-268	0.624	1.959	1.908	1.849	1.673	0.824	0.126	96	92	79	15	-80	2.81E-6	1.44E-5	4.85E-5
SF-295	0.753	2.609	2.391	2.453	1.943	0.324	0.139	88	92	64	-57	-82	1.31E-6	3.38E-6	8.76E-6
SF-539	0.891	3.026	2.929	2.936	2.777	0.298	0.041	95	96	88	-67	-95	1.77E-6	3.72E-6	7.82E-6
SNB-19	0.733	2.073	2.089	2.066	1.944	1.157	0.312	101	99	90	32	-58	4.86E-6	2.26E-5	8.24E-5
SNB-75	1.016	1.816	1.623	1.654	1.395	0.569	0.027	76	80	47	-44	-97	8.25E-7	3.30E-6	1.30E-5
U251	0.440	1.974	1.940	2.004	1.875	0.593	0.075	98	102	94	10	-83	3.32E-6	1.28E-5	4.41E-5
**Melanoma**															
LOX IMVI	0.363	2.851	2.769	2.680	2.050	0.479	0.141	97	93	68	5	-61	1.91E-6	1.18E-5	6.74E-5
MALME-3M	0.808	1.293	1.280	1.236	1.114	0.822	0.140	97	88	63	3	-83	1.65E-6	1.08E-5	4.15E-5
M14	0.445	1.970	1.893	1.937	1.528	0.251	0.071	95	98	71	-44	-84	1.53E-6	4.16E-6	1.43E-5
MDA-MB-435	0.540	2.806	2.613	2.622	0.708	0.222	0.034	91	92	7	-59	-94	3.13E-7	1.29E-6	7.32E-6
SK-MEL-2	1.151	2.399	2.389	2.339	2.078	0.962	0.157	99	95	74	-16	-86	1.85E-6	6.58E-6	3.02E-5
SK-MEL-28	0.924	2.674	2.563	2.599	2.278	0.272	0.031	94	96	77	-71	-97	1.53E-6	3.33E-6	7.26E-6
SK-MEL-5	0.624	2.290	2.122	2.185	1.268	0.218	0.066	90	94	39	-65	-90	6.21E-7	2.36E-6	7.16E-6
UACC-257	0.877	1.881	1.842	1.911	1.760	0.454	0.094	96	103	88	-48	-89	1.90E-6	4.42E-6	1.10E-5
UACC-62	0.864	2.646	2.609	2.609	1.957	1.051	0.282	98	98	61	10	-67	1.67E-6	1.36E-5	5.98E-5
**Ovarian Cancer**															
IGROV1	0.723	2.387	2.311	2.472	2.042	1.153	0.349	95	105	79	26	-52	3.52E-6	2.15E-5	9.50E-5
OVCAR-3	0.536	1.789	1.750	1.744	1.025	0.501	0.126	97	96	39	-7	-77	6.44E-7	7.16E-6	4.17E-5
OVCAR-4	0.755	1.713	1.598	1.694	1.480	0.976	0.230	88	98	76	23	-70	3.07E-6	1.77E-5	6.14E-5
OVCAR-5	0.819	1.935	1.773	1.817	1.740	1.145	0.156	85	89	83	29	-81	4.07E-6	1.84E-5	5.23E-5
OVCAR-8	0.633	2.534	2.546	2.609	2.483	0.904	0.605	101	104	97	14	-5	3.71E-6	5.75E-5	> 1.00E-4
NCI/ADR-RES	0.628	2.366	2.398	2.279	1.964	0.664	0.654	102	95	77	2	1	2.29E-6	> 1.00E-4	> 1.00E-4
SK-OV-3	0.925	1.990	1.923	1.995	1.872	1.111	0.067	94	100	89	17	-93	3.51E-6	1.44E-5	4.09E-5
**Renal Cancer**															
786-0	0.754	2.565	2.343	2.405	2.297	0.448	0.170	88	91	85	-41	-78	1.90E-6	4.75E-6	1.79E-5
A498	1.672	2.155	2.163	2.122	1.992	1.280	0.232	102	93	66	-23	-86	1.52E-6	5.48E-6	2.65E-5
ACHN	0.430	2.030	1.941	2.004	1.646	0.602	-0.005	94	98	76	11	-100	2.50E-6	1.25E-5	3.54E-5
RXF 393	1.082	1.587	1.556	1.542	1.403	0.741	0.094	94	91	64	-32	-91	1.39E-6	4.66E-6	2.04E-5
SN12C	0.934	3.056	2.902	2.999	2.838	1.366	0.615	93	97	90	20	-34	3.74E-6	2.36E-5	> 1.00E-4
TK-10	1.163	2.593	2.486	2.539	2.556	1.813	0.023	93	96	97	45	-98	8.18E-6	2.07E-5	4.63E-5
UO-31	0.856	2.028	1.801	1.788	1.602	0.900	0.084	81	80	64	4	-90	1.69E-6	1.10E-5	3.73E-5
**Prostate Cancer**															
PC-3	0.744	2.702	2.580	2.570	1.969	0.949	0.113	94	93	63	10	-85	1.74E-6	1.29E-5	4.31E-5
DU-145	0.428	1.801	1.783	1.815	1.499	0.528	-0.003	99	101	78	7	-100	2.49E-6	1.17E-5	3.42E-5
**Breast Cancer**															
MCF7	0.344	2.401	2.154	2.271	1.151	0.455	0.113	88	94	39	5	-67	6.34E-7	1.19E-5	5.80E-5
MDA-MB-231/ATCC	0.707	1.530	1.611	1.591	1.526	0.568	0.210	110	107	99	-20	-70	2.60E-6	6.84E-6	3.97E-5
HS 578T	1.023	1.947	1.861	1.869	1.782	1.190	0.760	91	91	82	18	-26	3.17E-6	2.58E-5	> 1.00E-4
BT-549	1.302	2.729	2.524	2.545	2.430	1.756	0.111	86	87	79	32	-91	4.12E-6	1.81E-5	4.61E-5
T-47D	0.733	1.279	1.167	1.180	1.094	0.728	0.372	80	82	66	-	-49	1.74E-6	9.74E-6	> 1.00E-4
MDA-MB-468	0.666	1.153	1.137	1.129	0.956	0.398	0.156	97	95	60	-40	-77	1.25E-6	3.95E-6	1.86E-5

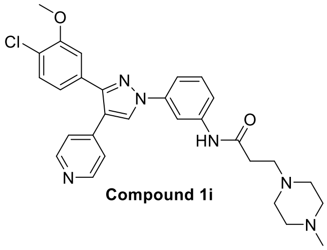

Compound 1i

[illegible]

	Time				Mean Optical Densities					Percent Growth					
Panel/Cell Line	Zero	Ctrl	-8.0	-7.0	-6.0	-5.0	-4.0	-8.0	-7.0	-6.0	-5.0	-4.0	GI50	TGI	LC50
Leukemia															
CCRF-CEM	0.570	2.279	2.289	2.335	1.896	0.483	0.143	101	103	78	-15	-75	1.98E-6	6.85E-6	3.82E-5
HL-60(TB)	0.799	2.350	2.180	2.221	1.475	0.367	0.245	89	92	44	-54	-69	7.35E-7	2.79E-6	9.07E-6
K-562	0.348	2.194	2.244	2.325	1.104	0.137	0.108	103	107	41	-61	-69	7.30E-7	2.53E-6	7.86E-6
MOLT-4	0.712	2.738	2.770	2.717	2.029	0.504	0.195	102	99	65	-29	-73	1.44E-6	4.89E-6	3.01E-5
RPMI-8226	0.772	2.691	2.693	2.559	2.310	0.452	0.239	100	93	80	-41	-69	1.77E-6	4.56E-6	2.04E-5
SR	0.538	1.845	1.764	1.787	0.903	0.332	0.227	94	96	28	-38	-58	4.71E-7	2.63E-6	3.96E-5
Non-Small Cell Lung Cancer															
A549/ATCC	0.430	2.029	1.930	1.957	1.444	0.308	0.024	94	95	63	-28	-95	1.40E-6	4.90E-6	2.12E-5
EKVX	0.756	2.545	2.427	2.300	2.002	0.930	0.093	93	86	70	10	-88	2.13E-6	1.26E-5	4.10E-5
HOP-62	0.608	1.560	1.513	1.440	1.210	0.393	0.042	95	87	63	-35	-93	1.36E-6	4.38E-6	1.79E-5
HOP-92	1.682	2.253	2.104	2.039	1.953	1.492	0.255	74	63	47	-11	-85	6.75E-7	6.42E-6	3.36E-5
NCI-H226	0.901	1.905	1.805	1.773	1.676	1.193	0.413	90	87	77	29	-54	3.67E-6	2.23E-5	8.90E-5
NCI-H23	0.746	2.362	2.334	2.323	2.029	0.768	0.140	98	98	79	1	-81	2.38E-6	1.04E-5	4.18E-5
NCI-H322M	0.771	2.156	2.030	2.045	1.969	0.864	0.018	91	92	87	7	-98	2.87E-6	1.16E-5	3.49E-5
NCI-H460	0.342	3.084	3.137	3.112	1.185	0.153	0.041	102	101	31	-55	-88	5.32E-7	2.28E-6	8.69E-6
NCI-H522	0.979	2.265	2.090	2.130	1.007	0.366	0.262	86	89	2	-63	-73	2.83E-7	1.08E-6	6.38E-6
Colon Cancer															
COLO 205	0.476	1.793	1.812	1.733	1.207	0.068	0.092	101	95	56	-86	-81	1.09E-6	2.47E-6	5.59E-6
HCC-2998	0.862	3.071	3.047	3.021	2.928	0.277	0.185	99	98	93	-68	-79	1.86E-6	3.79E-6	7.74E-6
HCT-116	0.405	2.776	2.836	2.726	1.539	0.287	0.035	103	98	48	-29	-91	9.04E-7	4.17E-6	2.16E-5
HCT-15	0.275	2.260	2.231	2.156	1.325	0.184	0.022	99	95	53	-33	-92	1.08E-6	4.11E-6	1.92E-5
HT29	0.301	1.875	1.855	1.851	0.510	0.156	0.079	99	98	13	-48	-74	3.71E-7	1.64E-6	1.18E-5
KM12	0.802	3.250	3.267	3.241	2.333	0.546	0.127	101	100	63	-32	-84	1.36E-6	4.59E-6	2.21E-5
SW-620	0.250	1.695	1.571	1.584	0.638	0.069	0.033	91	92	27	-73	-87	4.43E-7	1.86E-6	5.93E-6
CNS Cancer															
SF-268	0.624	2.048	2.030	1.981	1.619	0.264	0.088	99	95	70	-58	-86	1.43E-6	3.53E-6	8.69E-6
SF-295	0.753	2.596	2.501	2.450	1.664	0.277	0.180	95	92	49	-63	-76	9.68E-7	2.75E-6	7.63E-6
SF-539	0.891	3.080	2.942	2.813	2.688	0.118	0.049	94	88	82	-87	-95	1.55E-6	3.06E-6	6.05E-6
SNB-19	0.733	2.005	1.881	1.890	1.668	0.704	0.045	90	91	73	-4	-94	2.01E-6	8.89E-6	3.25E-5
SNB-75	1.016	1.773	1.637	1.552	1.348	0.504	0.012	82	71	44	-50	-99	5.91E-7	2.92E-6	9.89E-6
U251	0.440	2.039	1.946	1.936	1.451	0.195	0.045	94	94	63	-56	-90	1.29E-6	3.40E-6	8.96E-6
Melanoma															
LOX IMVI	0.363	2.963	2.828	2.783	1.633	0.030	0.027	94	92	48	-92	-93	9.22E-7	2.22E-6	5.04E-6
MALME-3M	0.808	1.322	1.247	1.226	1.053	0.160	0.120	85	81	48	-80	-85	8.47E-7	2.36E-6	5.80E-6
M14	0.445	1.876	1.847	1.765	1.226	0.127	0.025	98	92	55	-72	-94	1.09E-6	2.71E-6	6.75E-6
MDA-MB-435	0.540	2.874	2.801	2.590	0.634	0.145	0.018	97	88	4	-73	-97	2.83E-7	1.13E-6	5.00E-6
SK-MEL-2	1.151	2.372	2.355	2.340	1.731	0.354	0.069	99	97	47	-69	-94	8.89E-7	2.55E-6	6.84E-6
SK-MEL-28	0.924	2.668	2.698	2.589	2.282	0.131	0.016	102	95	78	-86	-98	1.48E-6	2.99E-6	6.04E-6
SK-MEL-5	0.624	2.237	2.156	2.056	1.191	0.028	0.037	95	89	35	-96	-94	5.29E-7	1.86E-6	4.48E-6
UACC-257	0.877	1.916	1.793	1.779	1.644	0.196	0.056	88	87	74	-78	-94	1.44E-6	3.07E-6	6.56E-6
UACC-62	0.864	2.577	2.508	2.458	1.759	0.255	0.090	96	93	52	-71	-90	1.04E-6	2.66E-6	6.80E-6
Ovarian Cancer															
IGROV1	0.723	2.357	2.433	2.391	1.863	1.001	0.208	105	102	70	17	-71	2.37E-6	1.56E-5	5.75E-5
OVCAR-3	0.536	1.913	1.947	1.908	0.688	0.352	0.011	102	100	11	-34	-98	3.63E-7	1.75E-6	1.76E-5
OVCAR-4	0.755	1.800	1.793	1.677	1.422	0.818	0.008	99	88	64	6	-99	1.73E-6	1.14E-5	3.42E-5
OVCAR-5	0.819	1.903	1.856	1.770	1.739	0.605	0.038	96	88	85	-26	-95	2.06E-6	5.82E-6	2.21E-5
OVCAR-8	0.633	2.553	2.517	2.492	2.275	0.732	0.247	98	97	86	5	-61	2.77E-6	1.20E-5	6.82E-5
NCI/ADR-RES	0.628	2.374	2.407	2.401	2.199	0.594	0.290	102	102	90	-5	-54	2.62E-6	8.76E-6	8.31E-5
SK-OV-3	0.925	2.145	2.158	2.083	1.793	0.823	0.006	101	95	71	-11	-99	1.81E-6	7.34E-6	2.76E-5
Renal Cancer															
786-0	0.754	2.540	2.604	2.357	2.132	0.250	0.080	104	90	77	-67	-89	1.54E-6	3.43E-6	7.64E-6
A498	1.672	2.048	1.973	1.927	1.819	1.044	0.192	80	68	39	-38	-89	4.12E-7	3.23E-6	1.75E-5
ACHN	0.430	2.011	2.052	1.933	1.415	0.480	0.005	103	95	62	3	-99	1.61E-6	1.07E-5	3.31E-5
RXF 393	1.082	1.625	1.551	1.536	1.378	0.341	0.101	86	84	55	-69	-91	1.09E-6	2.77E-6	7.07E-6
SN12C	0.934	3.003	2.874	2.874	2.495	0.879	0.088	94	94	75	-6	-91	2.05E-6	8.45E-6	3.31E-5
TK-10	1.163	2.620	2.568	2.635	2.502	1.450	0.007	96	101	92	20	-99	3.80E-6	1.46E-5	3.85E-5
UO-31	0.856	2.026	1.817	1.817	1.625	0.468	0.028	82	82	66	-45	-97	1.38E-6	3.91E-6	1.23E-5
Prostate Cancer															
PC-3	0.744	2.657	2.475	2.442	1.699	0.459	0.035	90	89	50	-38	-95	9.95E-7	3.68E-6	1.60E-5
DU-145	0.428	1.757	1.807	1.781	1.092	0.373	-0.010	104	102	50	-13	-100	9.97E-7	6.24E-6	2.67E-5
Breast Cancer															
MCF7	0.344	2.367	2.204	2.083	0.746	0.172	0.139	92	86	20	-50	-60	3.50E-7	1.92E-6	1.00E-5
MDA-MB-231/ATCC	0.707	1.608	1.525	1.541	1.463	0.248	0.085	91	93	84	-65	-88	1.69E-6	3.66E-6	7.93E-6
HS 578T	1.023	1.854	1.743	1.725	1.533	0.871	0.441	87	85	61	-15	-57	1.41E-6	6.39E-6	6.84E-5
BT-549	1.302	2.547	2.581	2.474	2.471	1.836	0.060	103	94	94	43	-95	7.26E-6	2.04E-5	4.69E-5
T-47D	0.733	1.383	1.367	1.348	1.246	0.736	0.251	98	95	79	.	-66	2.33E-6	1.01E-5	5.77E-5
MDA-MB-468	0.666	1.159	1.178	1.143	0.912	0.268	0.162	104	97	50	-60	-76	9.98E-7	2.85E-6	8.15E-6

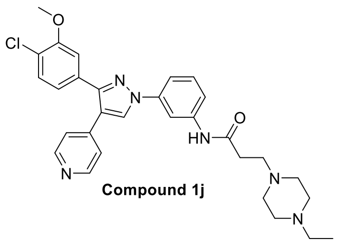

Compound 1j

	Time		[illegible] Mean Optical Densities					Percent Growth							
Panel/Cell Line	Zero	Ctrl	-8.0	-7.0	-6.0	-5.0	-4.0	-8.0	-7.0	-6.0	-5.0	-4.0	GI50	TGI	LC50
**Leukemia**															
CCRF-CEM	0.524	2.895	2.939	2.694	0.977	0.543	0.194	102	92	19	1	-63	3.74E-7	1.03E-5	6.26E-5
HL-60(TB)	0.851	3.000	2.945	3.071	0.919	0.372	0.256	97	103	3	-56	-70	3.40E-7	1.13E-6	7.84E-6
MOLT-4	0.709	2.919	2.885	2.960	1.565	0.623	0.228	98	101	39	-12	-68	6.61E-7	5.76E-6	4.78E-5
SR	0.641	2.552	2.440	2.275	1.210	0.322	0.254	94	85	30	-50	-60	4.33E-7	2.37E-6	1.05E-5
**Non-Small Cell Lung Cancer**															
A549/ATCC	0.443	1.870	1.625	1.792	0.970	0.153	0.044	83	95	37	-66	-90	5.93E-7	2.29E-6	7.05E-6
EKVX	0.916	2.514	2.498	2.334	1.748	0.690	0.110	99	89	52	-25	-88	1.06E-6	4.76E-6	2.51E-5
HOP-62	0.579	1.546	1.615	1.515	0.979	0.198	0.014	107	97	41	-66	-98	6.97E-7	2.43E-6	7.11E-6
HOP-92	1.160	1.986	1.916	1.823	1.542	1.033	0.209	92	80	46	-11	-82	7.76E-7	6.43E-6	3.54E-5
NCI-H226	1.235	2.663	2.570	2.449	2.044	1.125	0.621	93	85	57	-9	-50	1.26E-6	7.31E-6	> 1.00E-4
NCI-H23	0.742	2.217	2.172	2.143	1.503	0.430	0.162	97	95	52	-42	-78	1.04E-6	3.56E-6	1.66E-5
NCI-H322M	0.832	2.180	2.095	2.135	1.460	0.615	0.030	94	97	47	-26	-96	8.55E-7	4.37E-6	2.18E-5
NCI-H460	0.229	2.568	2.577	2.543	0.490	0.068	0.050	100	99	11	-70	-78	3.61E-7	1.37E-6	5.63E-6
NCI-H522	1.312	2.891	2.831	2.641	1.415	0.261	0.298	96	84	6	-80	-77	2.75E-7	1.19E-6	4.49E-6
**Colon Cancer**															
HCC-2998	0.786	2.766	2.689	2.625	2.101	0.066	0.151	96	93	66	-92	-81	1.27E-6	2.63E-6	5.45E-6
HCT-116	0.252	2.410	2.490	2.225	0.665	0.071	0.046	104	91	19	-72	-82	3.74E-7	1.62E-6	5.75E-6
HCT-15	0.311	2.032	1.978	1.822	0.862	0.107	0.019	97	88	33	-66	-94	4.91E-7	2.16E-6	6.93E-6
HT29	0.286	1.683	1.918	1.803	0.394	0.053	0.059	117	109	8	-82	-79	3.81E-7	1.22E-6	4.43E-6
KM12	0.534	2.694	2.734	2.606	0.939	0.188	0.098	102	96	19	-65	-82	3.94E-7	1.68E-6	6.65E-6
SW-620	0.319	2.312	2.251	2.027	0.682	0.048	0.050	97	86	18	-85	-84	3.38E-7	1.50E-6	4.57E-6
**CNS Cancer**															
SF-268	0.601	2.040	1.996	1.902	1.194	0.207	0.104	97	90	41	-66	-83	6.62E-7	2.43E-6	7.14E-6
SF-295	0.786	2.621	2.522	2.377	1.166	0.182	0.143	95	87	21	-77	-82	3.60E-7	1.63E-6	5.31E-6
SF-539	0.997	2.889	2.834	2.732	1.320	0.155	0.157	97	92	17	-84	-84	3.62E-7	1.47E-6	4.58E-6
SNB-19	0.802	2.451	2.283	2.306	1.665	0.661	0.079	90	91	52	-18	-90	1.08E-6	5.60E-6	2.80E-5
SNB-75	1.442	2.855	2.541	2.461	1.934	1.342	0.261	78	72	35	-7	-82	3.92E-7	6.82E-6	3.75E-5
U251	0.369	1.632	1.649	1.608	0.722	0.052	0.058	101	98	28	-86	-84	4.84E-7	1.76E-6	4.84E-6
**Melanoma**															
LOX IMVI	0.395	2.735	2.649	2.577	1.017	0.021	0.065	96	93	27	-95	-84	4.45E-7	1.66E-6	4.28E-6
MALME-3M	0.672	1.439	1.456	1.398	1.011	0.171	0.206	102	95	44	-75	-69	7.65E-7	2.35E-6	6.21E-6
M14	0.481	1.979	1.848	1.776	0.847	0.103	0.034	91	86	24	-79	-93	3.87E-7	1.73E-6	5.28E-6
MDA-MB-435	0.621	2.821	2.732	1.938	0.310	0.080	0.037	96	60	-50	-87	-94	1.23E-7	3.50E-7	9.97E-7
SK-MEL-2	1.030	2.177	2.328	2.204	1.679	0.179	0.165	113	102	57	-83	-84	1.11E-6	2.55E-6	5.83E-6
SK-MEL-28	0.846	2.338	2.305	2.182	1.917	0.170	0.098	98	90	72	-80	-88	1.39E-6	2.97E-6	6.35E-6
SK-MEL-5	0.815	2.680	2.633	2.445	1.456	0.030	0.078	97	87	34	-96	-90	5.07E-7	1.83E-6	4.42E-6
UACC-257	0.878	1.719	1.621	1.652	1.275	0.154	0.038	88	92	47	-82	-96	8.64E-7	2.31E-6	5.62E-6
UACC-62	1.062	2.794	2.690	2.583	1.838	0.252	0.088	94	88	45	-76	-92	7.57E-7	2.34E-6	6.07E-6
**Ovarian Cancer**															
IGROV1	0.689	2.243	2.258	2.191	1.509	0.544	0.284	101	97	53	-21	-59	1.09E-6	5.18E-6	5.85E-5
OVCAR-3	0.527	1.812	1.895	1.772	0.671	0.210	0.021	106	97	11	-60	-96	3.52E-7	1.43E-6	7.19E-6
OVCAR-4	0.805	1.853	1.864	1.699	1.315	0.750	0.011	101	85	49	-7	-99	9.19E-7	7.53E-6	2.95E-5
OVCAR-5	0.721	2.001	1.973	1.853	1.547	0.507	0.103	98	88	64	-30	-86	1.42E-6	4.84E-6	2.30E-5
OVCAR-8	0.515	1.870	1.874	1.879	1.333	0.292	0.149	100	101	60	-43	-71	1.26E-6	3.82E-6	1.74E-5
NCI/ADR-RES	0.622	2.051	2.039	1.986	1.478	0.406	0.324	99	95	60	-35	-48	1.27E-6	4.29E-6	> 1.00E-4
SK-OV-3	1.240	2.206	2.361	2.298	1.930	1.125	0.013	116	109	71	-9	-99	1.84E-6	7.67E-6	2.84E-5
**Renal Cancer**															
786-0	0.735	2.690	2.669	2.556	1.559	0.140	0.082	99	93	42	-81	-89	7.01E-7	2.20E-6	5.60E-6
A498	1.385	2.317	2.247	2.206	1.779	0.633	0.090	92	88	42	-54	-94	6.76E-7	2.74E-6	9.02E-6
ACHN	0.422	1.927	1.949	1.792	1.101	0.173	0.004	101	91	45	-59	-99	7.82E-7	2.71E-6	8.17E-6
RXF 393	1.028	1.532	1.439	1.391	1.106	0.163	0.135	82	72	15	-84	-87	2.44E-7	1.43E-6	4.54E-6
SN12C	0.616	2.379	2.300	2.229	1.308	0.332	0.130	96	91	39	-46	-79	6.23E-7	2.88E-6	1.31E-5
TK-10	0.907	1.749	1.732	1.773	1.553	0.607	0.015	98	103	77	-33	-98	1.75E-6	5.00E-6	1.82E-5
UO-31	0.872	2.337	2.168	2.143	1.585	0.243	0.056	88	87	49	-72	-94	9.22E-7	2.53E-6	6.55E-6
**Prostate Cancer**															
PC-3	0.561	2.342	2.264	2.121	1.093	0.181	0.047	96	88	30	-68	-92	4.47E-7	2.02E-6	6.58E-6
DU-145	0.433	1.868	1.890	1.823	0.765	0.404	.	101	97	23	-7	-100	4.32E-7	5.96E-6	2.91E-5
**Breast Cancer**															
MCF7	0.432	2.666	2.429	2.123	0.779	0.082	0.180	89	76	16	-81	-58	2.67E-7	1.45E-6	4.76E-6
MDA-MB-231/ATCC	0.685	1.881	1.896	1.896	1.411	0.206	0.104	101	101	61	-70	-85	1.21E-6	2.92E-6	7.04E-6
HS 578T	1.271	2.286	2.153	2.105	1.608	0.973	0.560	87	82	33	-23	-56	4.54E-7	3.85E-6	6.55E-5
BT-549	1.110	2.305	2.200	2.124	1.873	0.722	0.096	91	85	64	-35	-91	1.38E-6	4.43E-6	1.85E-5
T-47D	0.802	1.817	1.838	1.723	1.276	0.620	0.298	102	91	47	-23	-63	8.41E-7	4.70E-6	4.77E-5
MDA-MB-468	0.972	2.138	2.127	2.042	1.091	0.403	0.281	99	92	10	-59	-71	3.25E-7	1.41E-6	7.51E-6

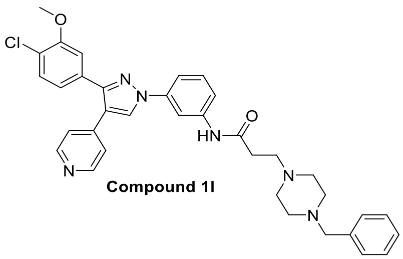

Compound 1l

			Log10 Concentration														
	Time		Mean Optical Densities					Percent Growth									
Panel/Cell Line	Zero	Ctrl	-8.0	-7.0	-6.0	-5.0	-4.0	-8.0	-7.0	-6.0	-5.0	-4.0	GI50	TGI	LC50
Leukemia															
CCRF-CEM	0.524	2.781	2.778	2.524	1.486	0.541	0.543	100	89	43	1	1	6.91E-7	> 1.00E-4	> 1.00E-4
HL-60(TB)	0.851	2.833	2.881	2.941	1.568	0.666	0.591	102	105	36	-22	-31	6.31E-7	4.21E-6	> 1.00E-4
MOLT-4	0.709	2.799	2.652	2.588	1.844	0.590	0.600	93	90	54	-17	-15	1.15E-6	5.81E-6	> 1.00E-4
SR	0.641	2.471	2.303	2.153	1.149	0.369	0.369	91	83	28	-43	-43	3.93E-7	2.48E-6	> 1.00E-4
Non-Small Cell Lung Cancer															
A549/ATCC	0.443	1.592	1.484	1.598	1.182	0.486	0.201	91	101	64	4	-55	1.72E-6	1.16E-5	8.33E-5
EKVX	0.916	2.438	2.373	2.205	1.853	0.994	0.771	96	85	62	5	-16	1.60E-6	1.75E-5	> 1.00E-4
HOP-62	0.579	1.606	1.550	1.512	1.163	0.658	0.146	95	91	57	8	-75	1.38E-6	1.24E-5	5.00E-5
HOP-92	1.160	1.866	1.821	1.687	1.493	0.862	0.338	94	75	47	-26	-71	7.90E-7	4.44E-6	3.45E-5
NCI-H226	1.235	2.712	2.575	2.576	2.318	1.575	1.101	91	91	73	23	-11	2.91E-6	4.77E-5	> 1.00E-4
NCI-H23	0.742	2.287	2.297	2.275	1.766	0.725	0.606	101	99	66	-2	-18	1.73E-6	9.26E-6	> 1.00E-4
NCI-H322M	0.832	2.125	1.963	1.966	1.825	0.953	0.447	87	88	77	9	-46	2.49E-6	1.47E-5	> 1.00E-4
NCI-H460	0.229	2.490	2.539	2.410	0.598	0.037	-0.018	102	96	16	-84	-100	3.80E-7	1.46E-6	4.59E-6
NCI-H522	1.312	3.056	2.855	2.839	1.660	0.495	0.410	88	88	20	-62	-69	3.59E-7	1.75E-6	7.09E-6
Colon Cancer															
HCC-2998	0.786	2.696	2.677	2.619	2.344	0.326	0.048	99	96	82	-59	-94	1.68E-6	3.82E-6	8.69E-6
HCT-116	0.252	2.270	2.208	2.052	0.751	0.058	-0.051	97	89	25	-77	-100	4.06E-7	1.75E-6	5.43E-6
HCT-15	0.311	1.983	1.934	1.758	0.998	0.215	0.116	97	87	41	-31	-63	6.36E-7	3.72E-6	3.99E-5
HT29	0.286	1.871	1.781	1.682	0.469	0.147	0.036	94	88	12	-49	-87	3.14E-7	1.55E-6	1.08E-5
KM12	0.534	2.631	2.664	2.615	1.267	0.403	0.265	102	99	35	-25	-50	5.84E-7	3.86E-6	9.67E-5
SW-620	0.319	2.219	2.190	2.056	0.916	0.070	0.045	98	91	31	-78	-86	4.90E-7	1.93E-6	5.53E-6
CNS Cancer															
SF-268	0.601	2.079	2.033	1.883	1.367	0.380	0.174	97	87	52	-37	-71	1.05E-6	3.84E-6	2.42E-5
SF-295	0.786	2.591	2.488	2.378	1.369	0.163	0.351	94	88	32	-79	-55	4.82E-7	1.95E-6	5.47E-6
SF-539	0.997	2.782	2.743	2.651	2.028	0.176	0.328	98	93	58	-82	-67	1.14E-6	2.58E-6	5.87E-6
SNB-19	0.802	2.384	2.238	2.205	2.019	1.359	0.625	91	89	77	35	-22	4.42E-6	4.12E-5	> 1.00E-4
SNB-75	1.442	2.858	2.577	2.470	2.328	1.386	1.079	80	73	63	-4	-25	1.55E-6	8.73E-6	> 1.00E-4
U251	0.369	1.686	1.661	1.690	0.973	0.155	-0.008	98	100	46	-58	-100	8.39E-7	2.76E-6	8.35E-6
Melanoma															
LOX IMVI	0.395	2.793	2.727	2.678	1.357	0.020	-0.001	97	95	40	-95	-100	6.61E-7	1.98E-6	4.64E-6
MALME-3M	0.672	1.377	1.379	1.345	1.027	0.179	0.279	100	95	50	-73	-58	1.01E-6	2.55E-6	6.47E-6
M14	0.481	1.944	1.889	1.759	1.110	0.086	0.110	96	87	43	-82	-77	6.96E-7	2.21E-6	5.54E-6
MDA-MB-435	0.621	2.779	2.695	2.523	0.538	0.061	0.177	96	88	-13	-90	-72	2.37E-7	7.37E-7	2.99E-6
SK-MEL-2	1.030	2.350	2.352	2.345	1.887	0.615	0.386	100	100	65	-40	-63	1.39E-6	4.14E-6	2.73E-5
SK-MEL-28	0.846	2.254	2.253	2.131	1.818	0.140	0.209	100	91	69	-84	-75	1.33E-6	2.84E-6	6.03E-6
SK-MEL-5	0.815	2.732	2.652	2.479	1.899	0.010	0.056	96	87	57	-99	-93	1.10E-6	2.31E-6	4.85E-6
UACC-257	0.878	1.658	1.583	1.577	1.549	0.259	0.271	90	90	86	-71	-69	1.70E-6	3.55E-6	7.40E-6
UACC-62	1.062	2.786	2.689	2.650	2.045	0.265	0.178	94	92	57	-75	-83	1.13E-6	2.70E-6	6.46E-6
Ovarian Cancer															
IGROV1	0.689	2.137	2.146	2.114	1.399	0.650	0.385	101	98	49	-6	-44	9.54E-7	7.88E-6	> 1.00E-4
OVCAR-3	0.527	1.862	1.891	1.861	0.744	0.268	0.046	102	100	16	-49	-91	3.95E-7	1.77E-6	1.04E-5
OVCAR-4	0.805	1.767	1.739	1.663	1.393	0.971	0.717	97	89	61	17	-11	1.80E-6	4.10E-5	> 1.00E-4
OVCAR-5	0.721	1.938	1.889	1.828	1.761	0.863	0.727	96	91	85	12	.	3.02E-6	> 1.00E-4	> 1.00E-4
OVCAR-8	0.515	1.985	2.009	2.037	1.768	0.558	0.199	102	104	85	3	-61	2.68E-6	1.11E-5	6.64E-5
NCI/ADR-RES	0.622	2.143	2.158	2.095	0.988	0.354	0.487	101	97	24	-43	-22	4.40E-7	2.28E-6	> 1.00E-4
SK-OV-3	1.240	2.219	2.284	2.308	2.428	1.411	0.768	107	109	121	17	-38	4.86E-6	2.06E-5	> 1.00E-4
Renal Cancer															
786-0	0.735	2.599	2.579	2.490	1.988	0.199	0.027	99	94	67	-73	-96	1.33E-6	3.02E-6	6.86E-6
A498	1.385	2.306	2.223	2.202	1.948	1.208	0.125	91	89	61	-13	-91	1.41E-6	6.71E-6	2.99E-5
ACHN	0.422	1.760	1.821	1.697	1.139	0.524	0.154	105	95	54	8	-64	1.20E-6	1.28E-5	6.44E-5
RXF 393	1.028	1.625	1.563	1.586	1.280	0.252	0.183	90	93	42	-75	-82	7.05E-7	2.28E-6	6.07E-6
SN12C	0.616	2.334	2.276	2.294	1.709	0.996	0.308	97	98	64	22	-50	2.13E-6	2.03E-5	> 1.00E-4
TK-10	0.907	1.739	1.676	1.683	1.630	0.972	0.148	92	93	87	8	-84	2.93E-6	1.22E-5	4.28E-5
UO-31	0.872	2.203	1.989	1.958	1.515	0.778	0.219	84	82	48	-11	-75	8.87E-7	6.56E-6	4.08E-5
Prostate Cancer															
PC-3	0.561	1.967	1.879	1.848	1.162	0.608	0.284	94	92	43	3	-49	7.09E-7	1.16E-5	> 1.00E-4
DU-145	0.433	1.835	1.905	1.872	1.296	0.467	0.168	105	103	61	2	-61	1.56E-6	1.09E-5	6.64E-5
Breast Cancer															
MCF7	0.432	2.556	2.471	2.447	0.824	0.357	0.422	96	95	18	-17	-2	3.86E-7	3.27E-6	> 1.00E-4
MDA-MB-231/ATCC	0.685	1.862	1.825	1.799	1.631	0.268	0.301	97	95	80	-61	-56	1.64E-6	3.71E-6	8.37E-6
HS 578T	1.271	2.278	2.138	2.113	1.782	0.904	0.797	86	84	51	-29	-37	1.02E-6	4.34E-6	> 1.00E-4
BT-549	1.110	2.154	2.142	2.050	1.953	1.047	0.581	99	90	81	-6	-48	2.27E-6	8.59E-6	> 1.00E-4
T-47D	0.802	1.872	1.718	1.709	1.633	0.962	0.650	86	85	78	15	-19	2.76E-6	2.76E-5	> 1.00E-4
MDA-MB-468	0.972	2.182	2.120	2.069	1.296	1.052	0.730	95	91	27	7	-25	4.33E-7	1.62E-5	> 1.00E-4

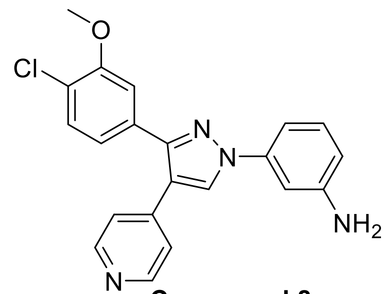

Compound 8

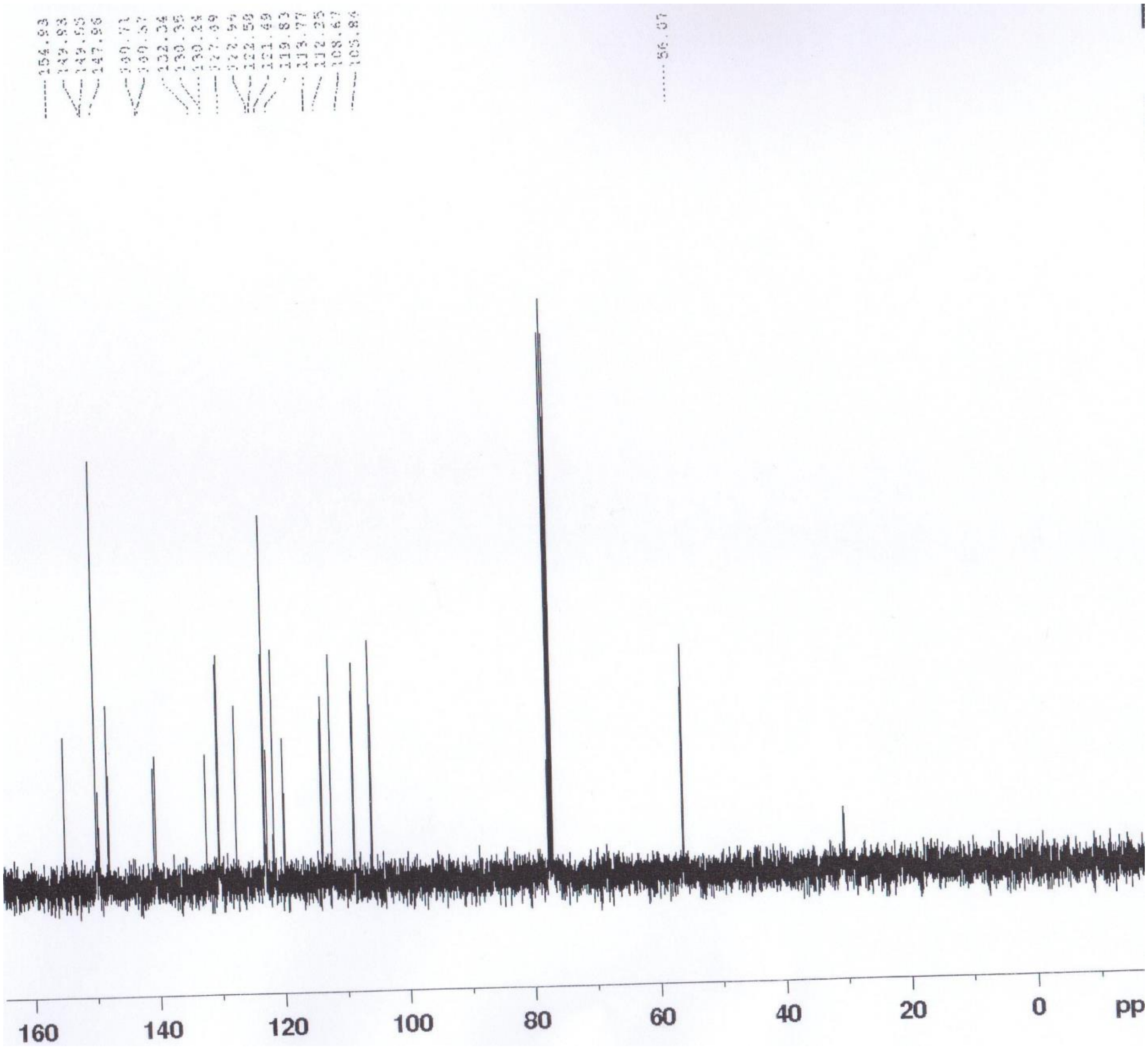

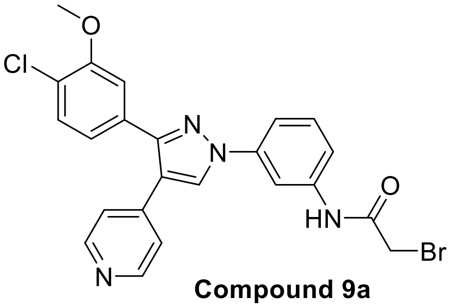
O
Cl
N
N
HN
O
Br
N

Compound 9a

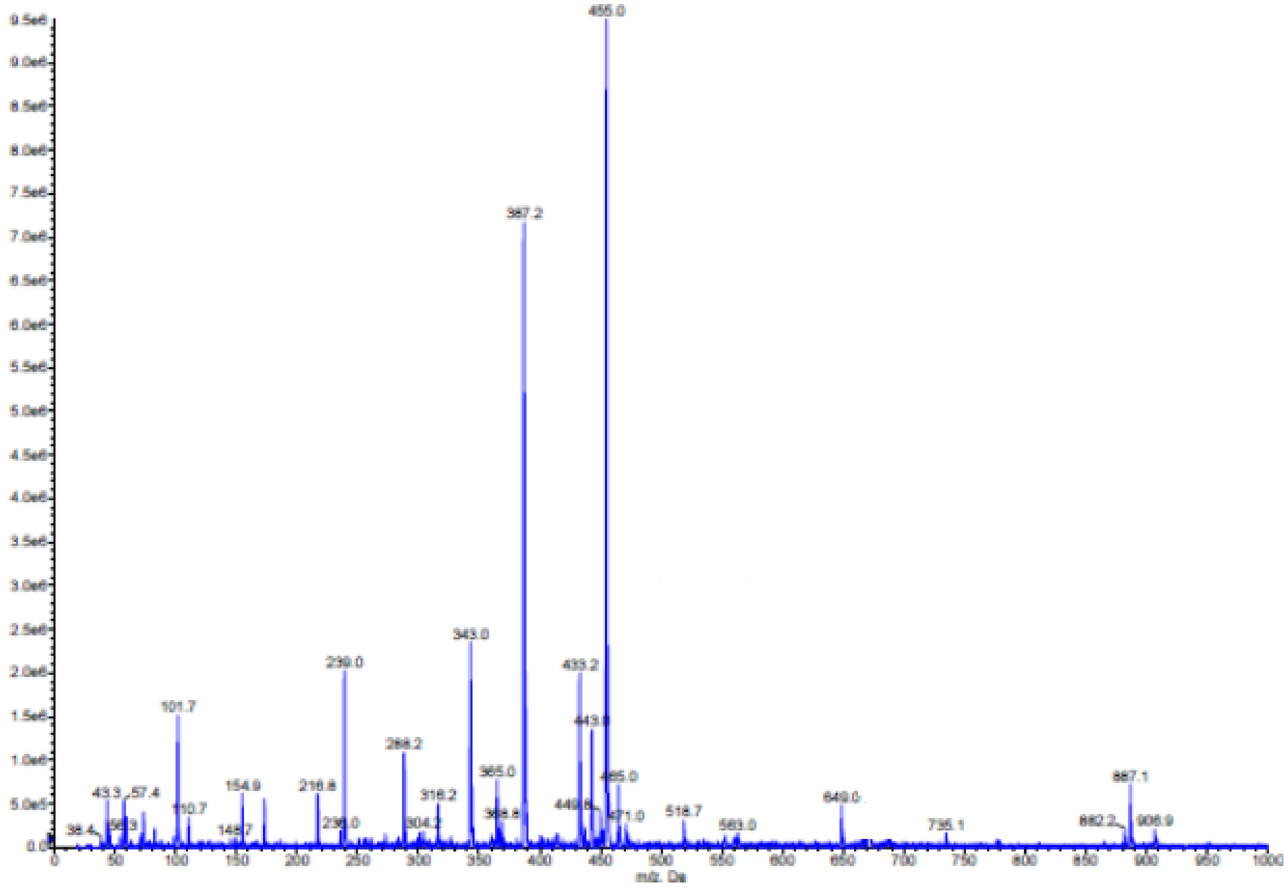
455.0
387.2
343.0
239.0
433.2
101.7
288.2
365.0
154.9
216.8
649.0
887.1
518.7
735.1
906.9
m/z, Da

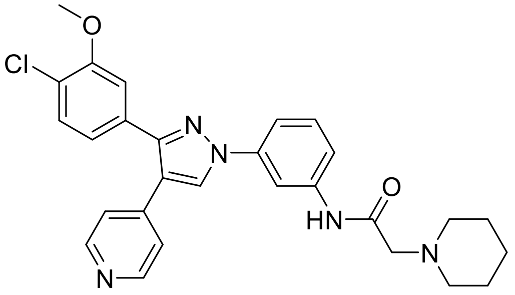

O
Cl
N
N
N
HN
O
N

Compound 1a

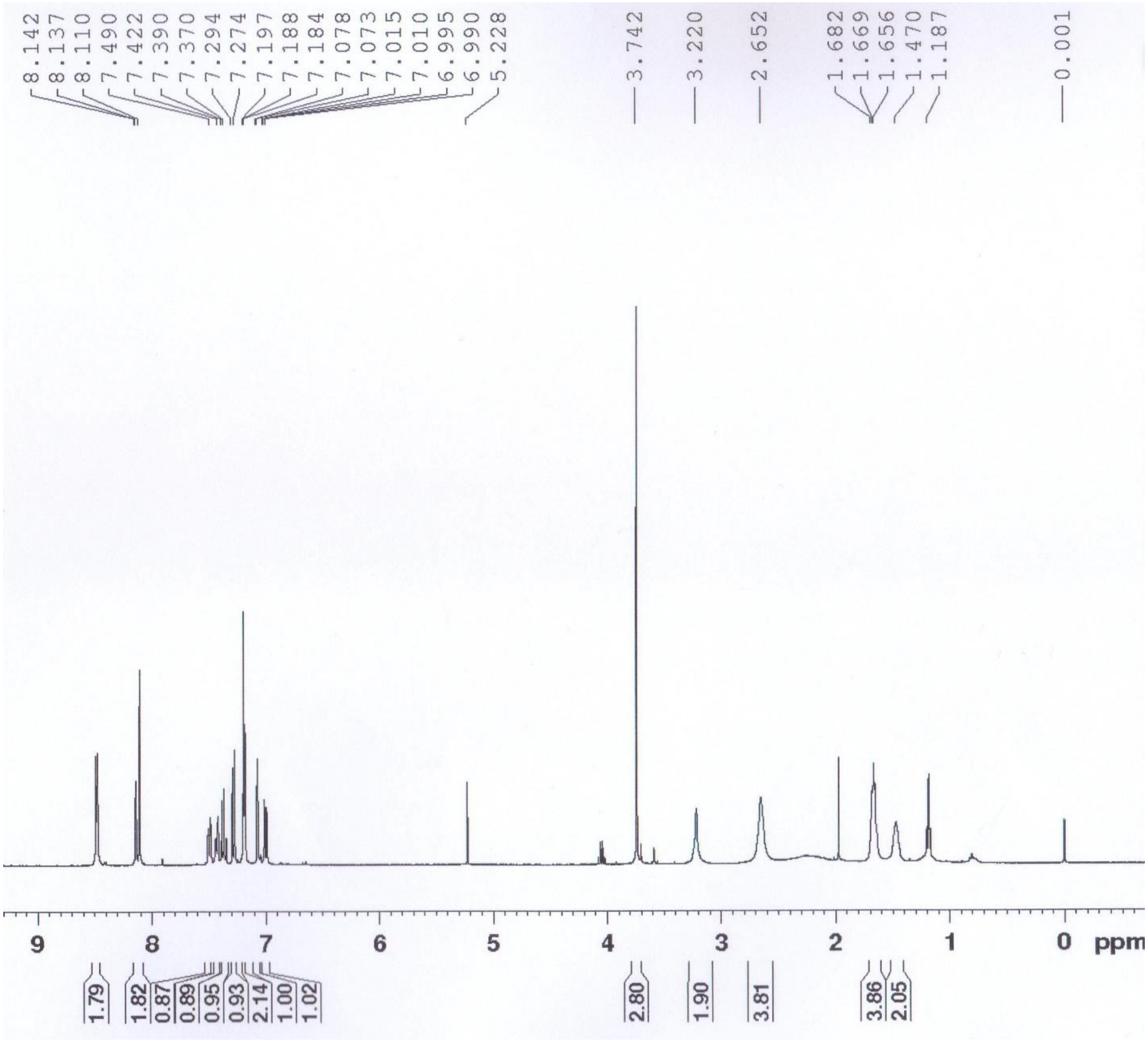

8.142
8.137
8.110
7.490
7.422
7.390
7.370
7.294
7.274
7.197
7.188
7.184
7.078
7.073
7.015
7.010
6.995
6.990
5.228
3.742
3.220
2.652
1.682
1.669
1.656
1.470
1.187
0.001
9
8
7
6
5
4
3
2
1
0
ppm
1.79
1.82
0.87
0.89
0.95
0.93
2.14
1.00
1.02
2.80
1.90
3.81
3.86
2.05

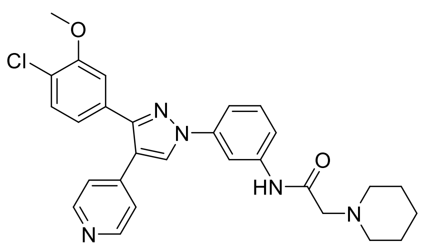

Compound 1a

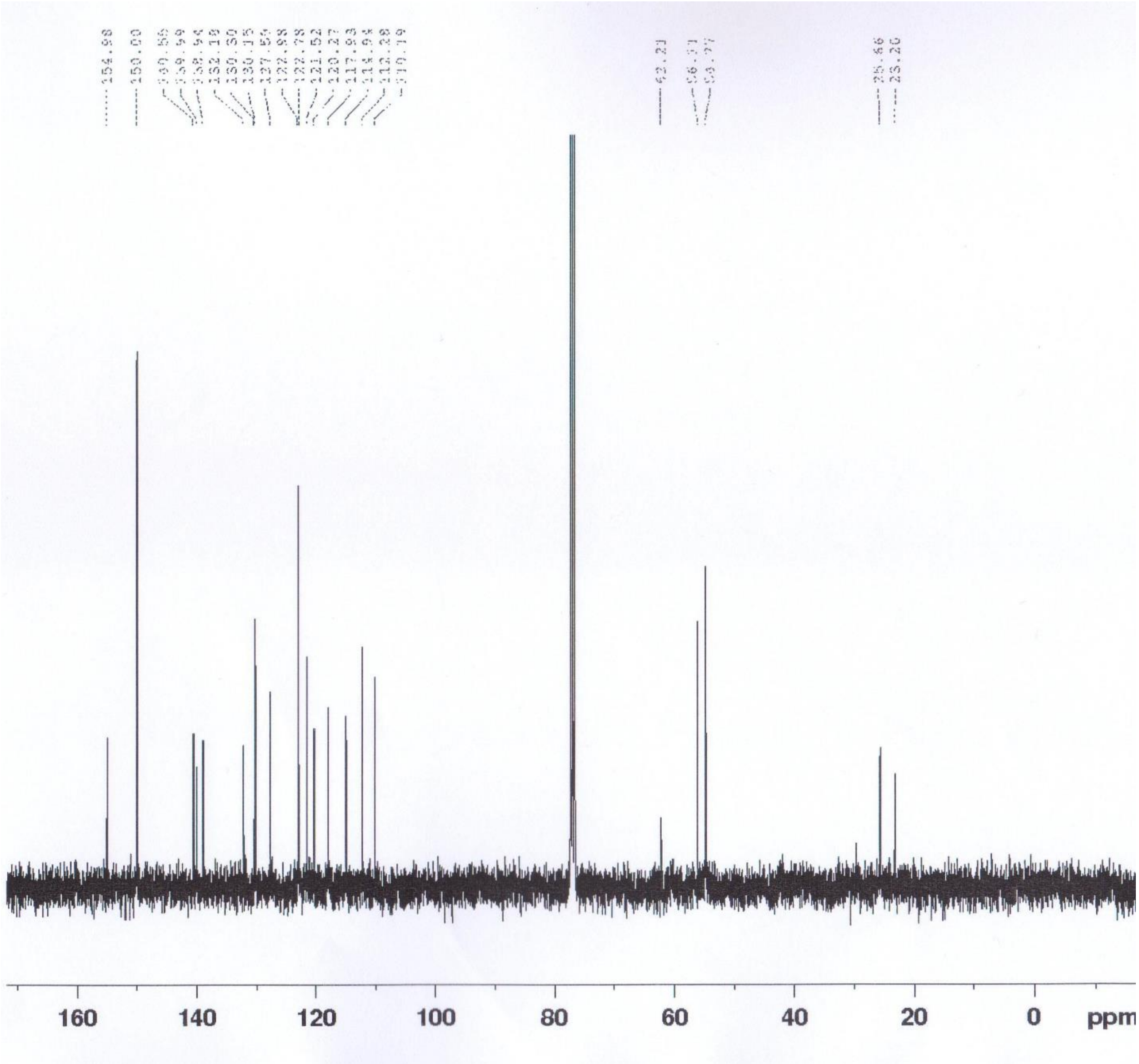

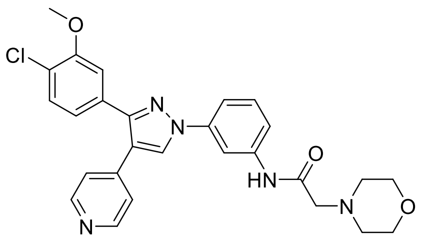

Compound 1b

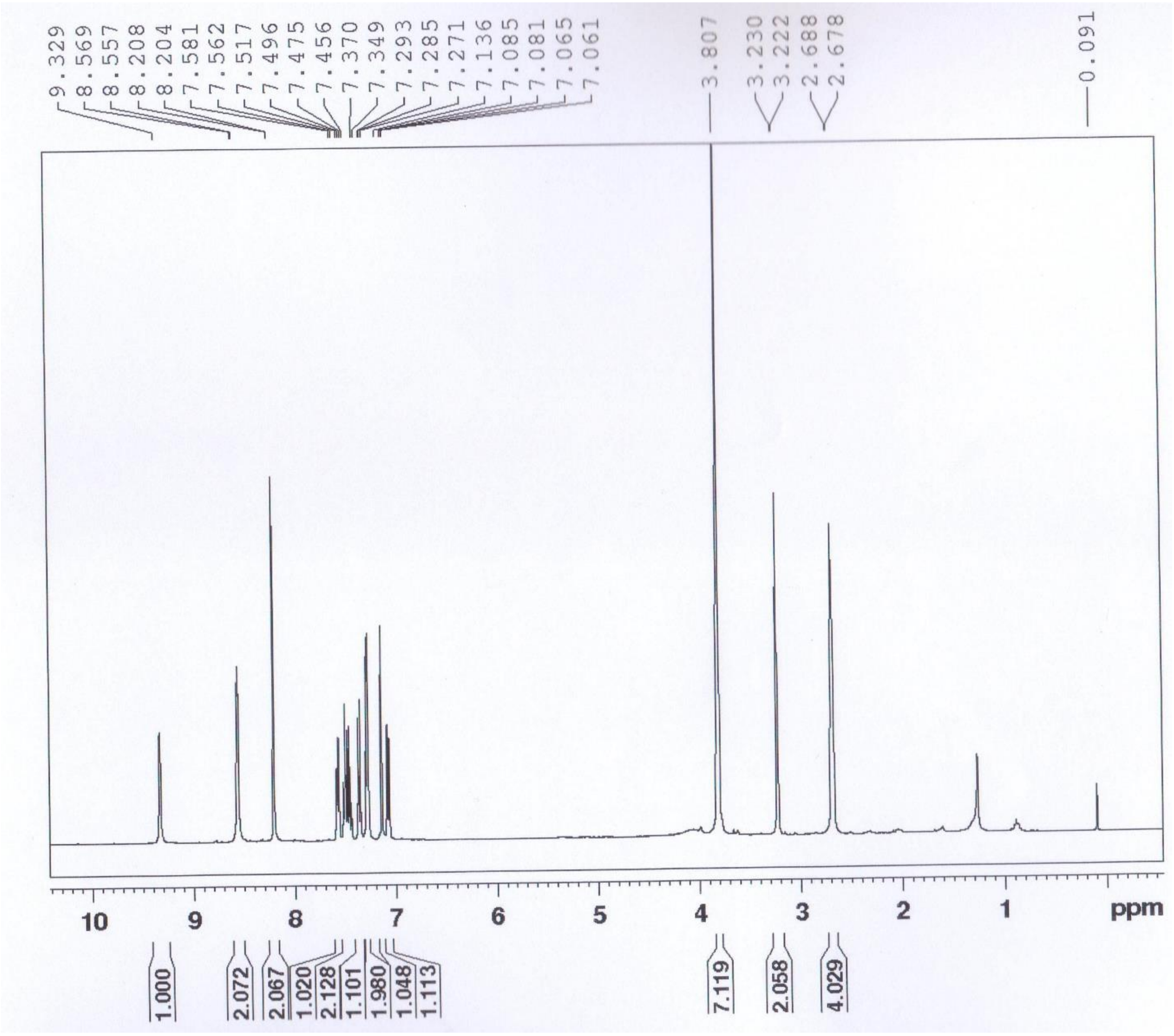

9.329
8.569
8.557
8.208
8.204
7.581
7.562
7.517
7.496
7.475
7.456
7.370
7.349
7.293
7.285
7.271
7.136
7.085
7.081
7.065
7.061
3.807
3.230
3.222
2.688
2.678
0.091
10 9 8 7 6 5 4 3 2 1 ppm
1.000
2.072
2.067
1.020
2.128
1.101
1.980
1.048
1.113
7.119
2.058
4.029

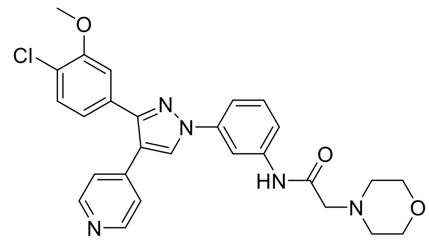

Compound 1b

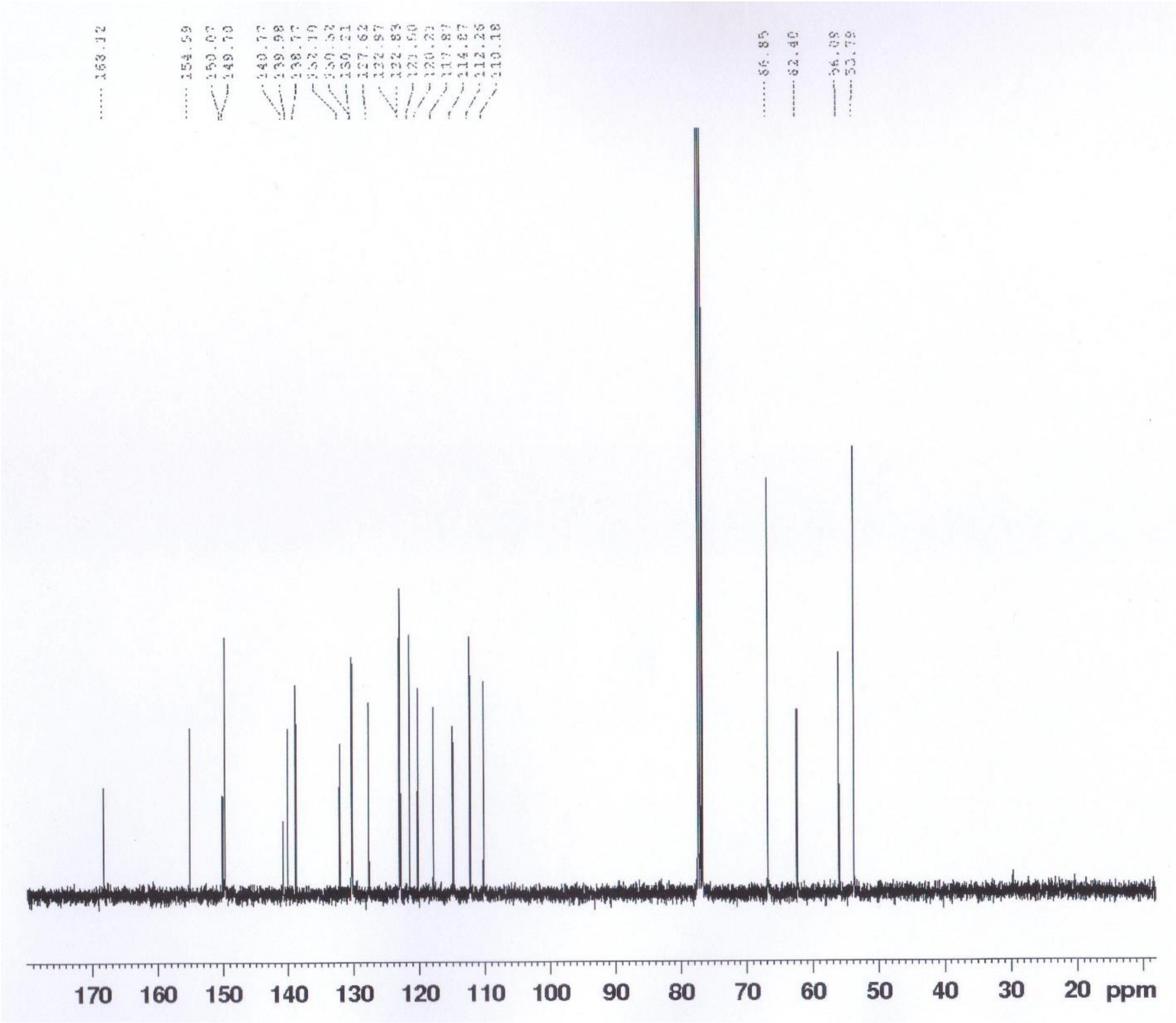

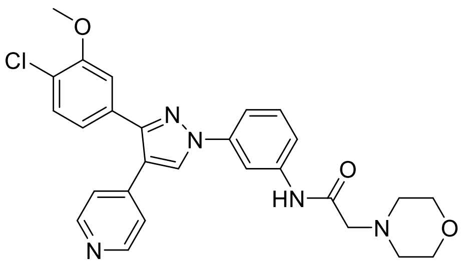

Compound 1b
Chemical Formula: $C_{27}H_{26}ClN_5O_3$
m/z + H^+: 505.1

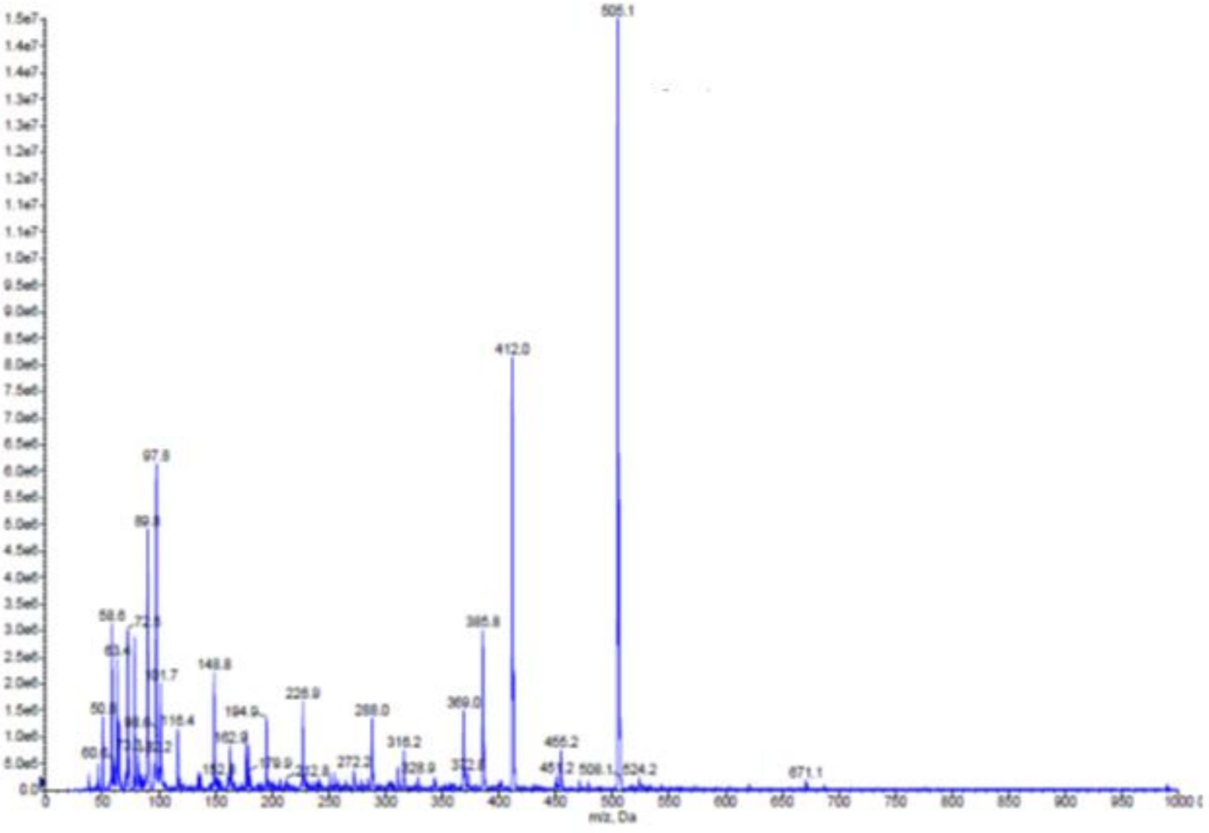

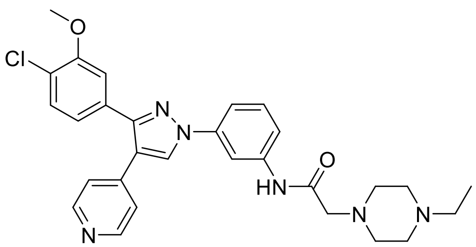

Compound 1d

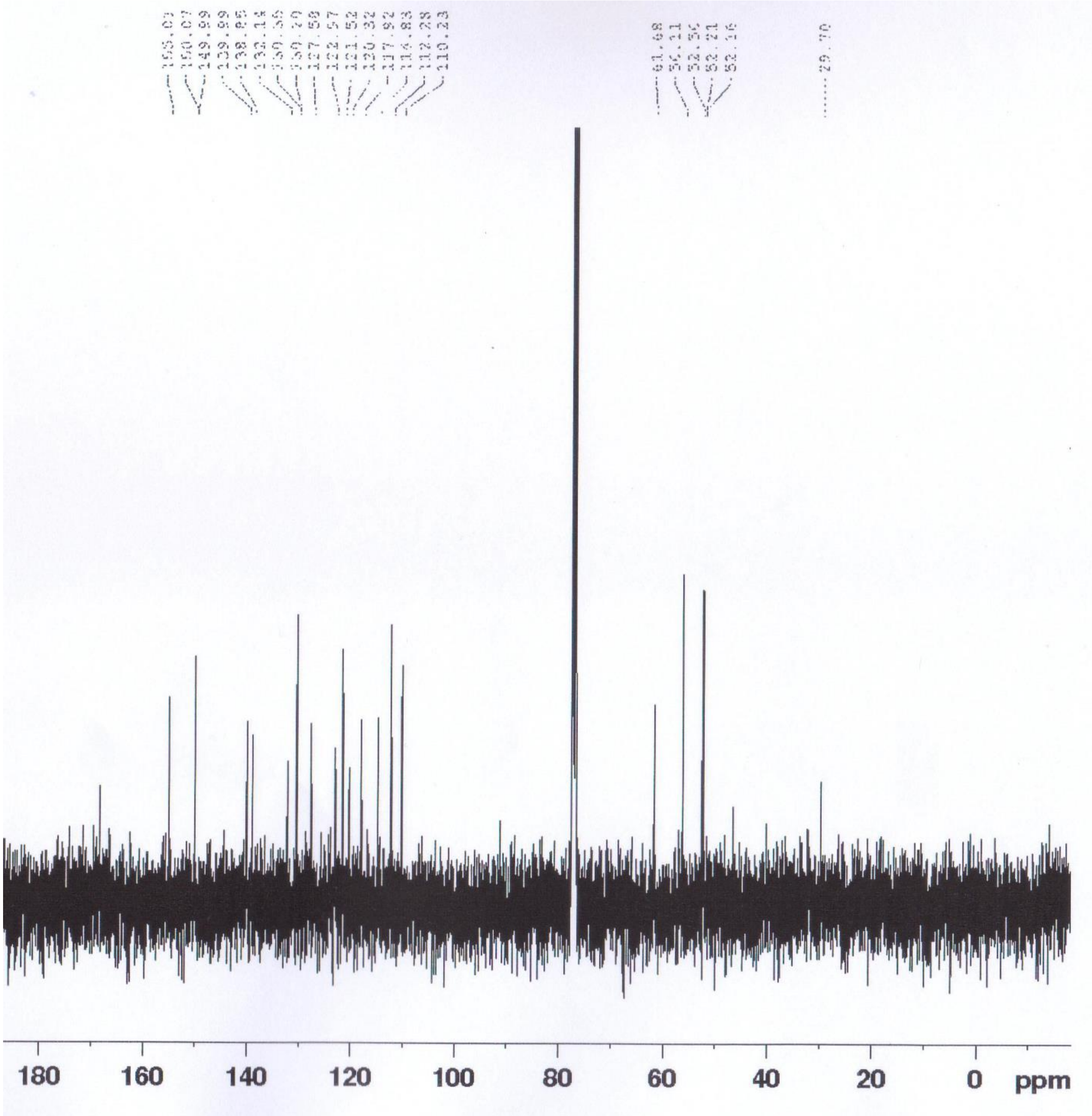
150.07
149.99
139.99
138.85
127.58
122.57
121.52
120.32
117.92
114.83
112.28
110.23
61.68
56.11
52.56
52.21
52.16
29.70
180
160
140
120
100
80
60
40
20
0
ppm

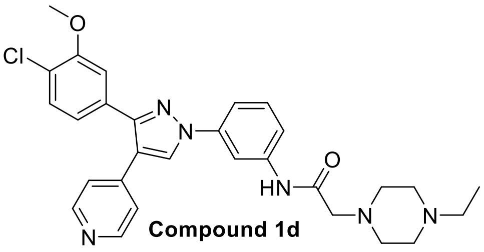

Chemical Formula: $C_{29}H_{31}ClN_6O_2$
m/z: 531.0

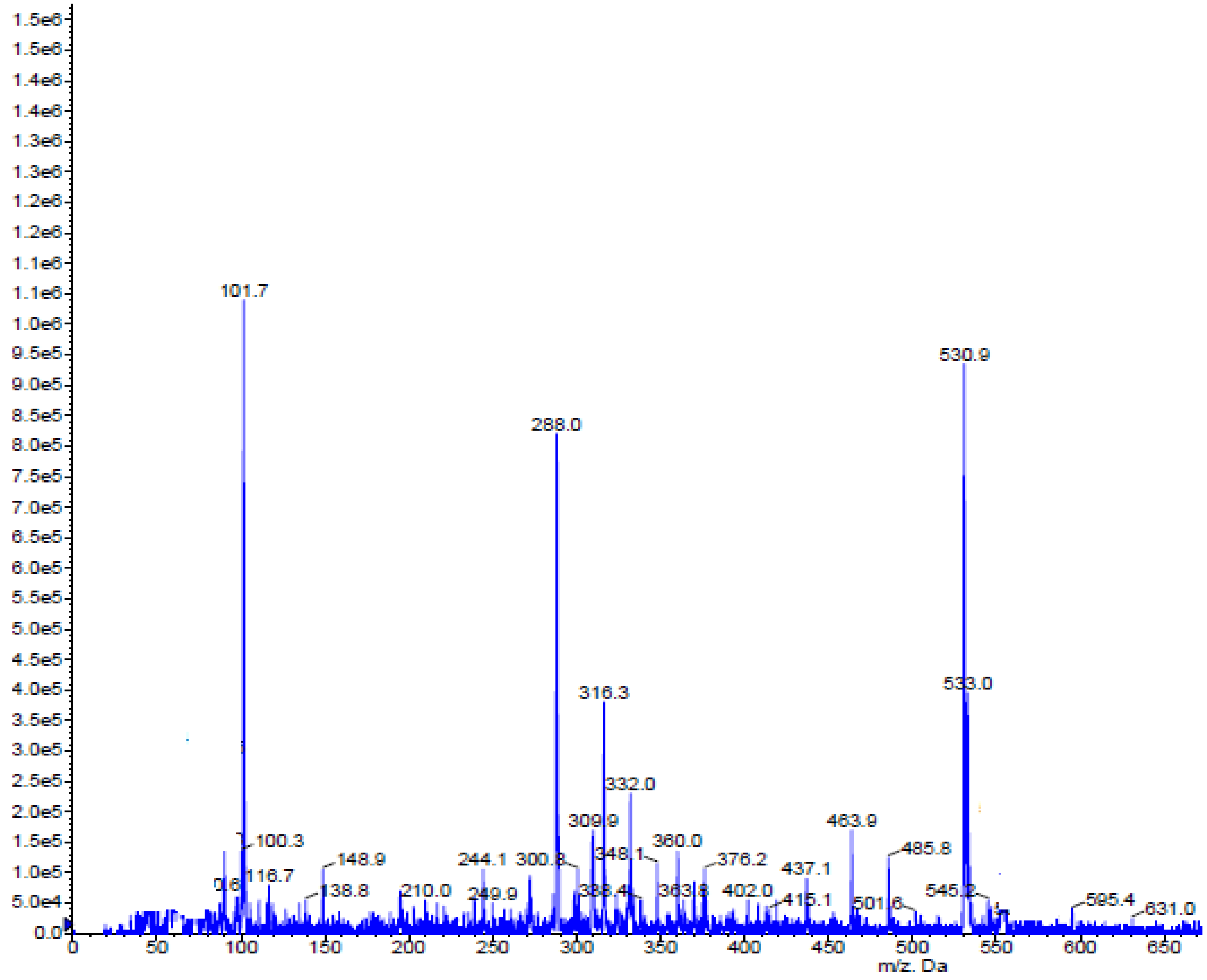

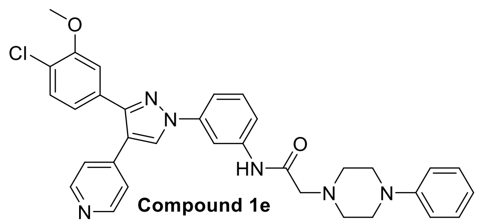
O
Cl
N
N
N
O
HN
N
N
N

Compound 1e

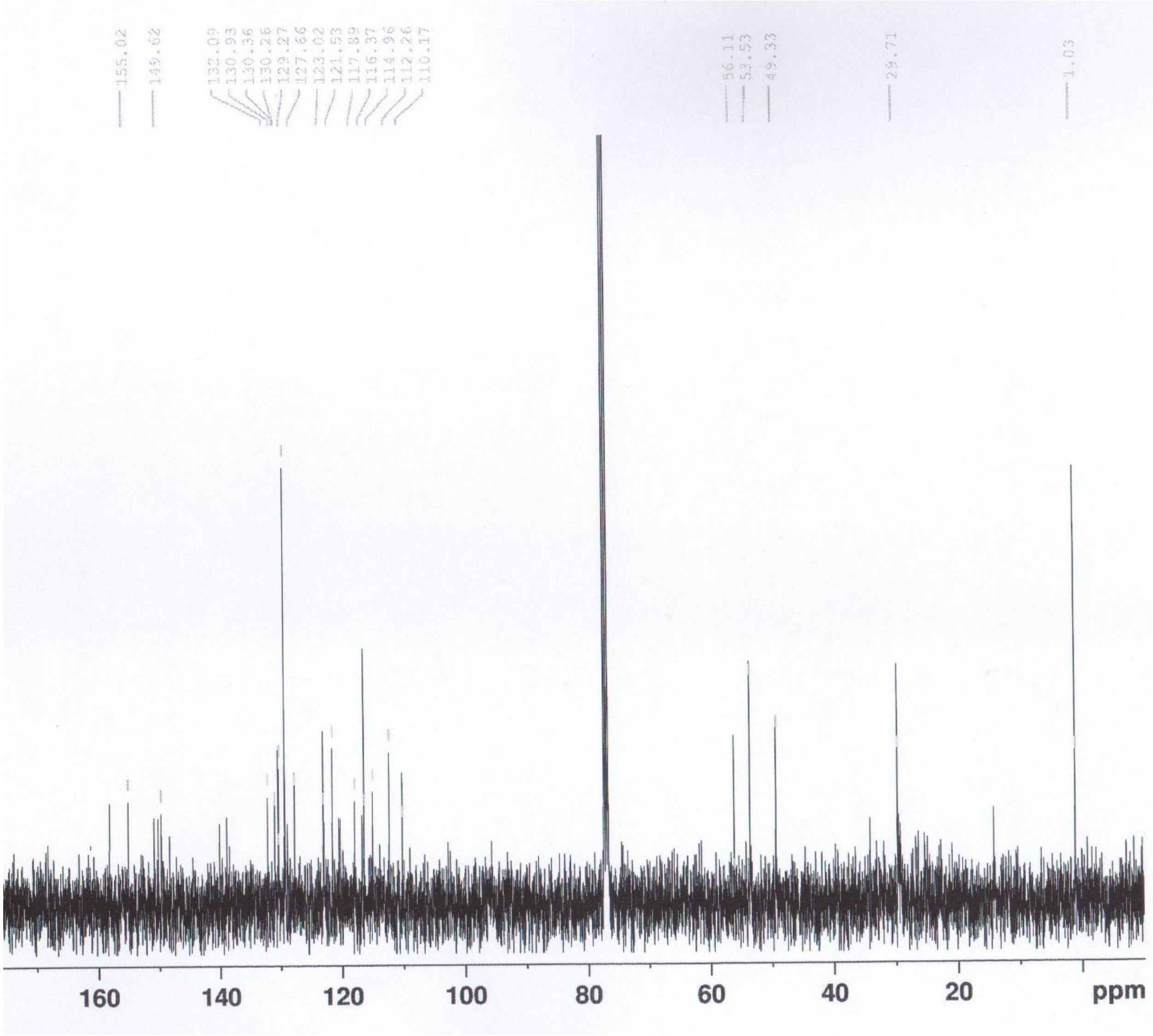
155.02
149.62
132.09
130.93
130.36
130.26
129.27
127.66
123.02
121.53
117.89
116.37
114.96
112.26
110.17
56.11
53.53
49.33
29.71
1.03
160
140
120
100
80
60
40
20
ppm

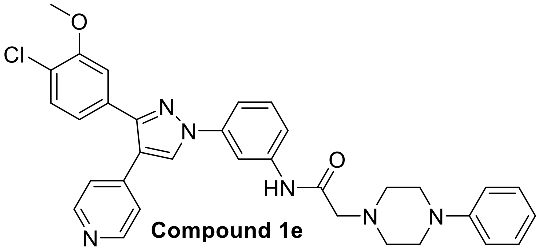

Compound 1e

Chemical Formula: $C_{33}H_{31}ClN_6O_2$

m/z:+ $2H^+$ 579.9

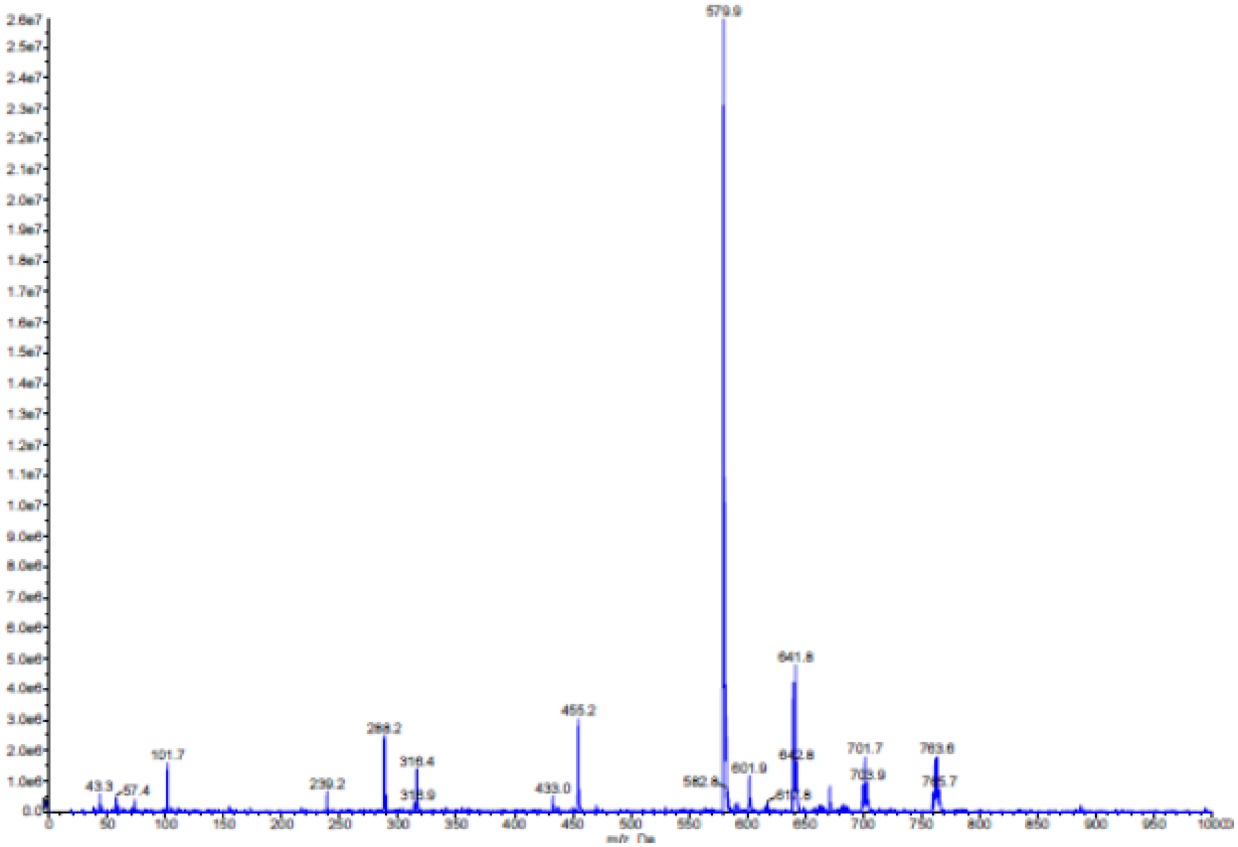

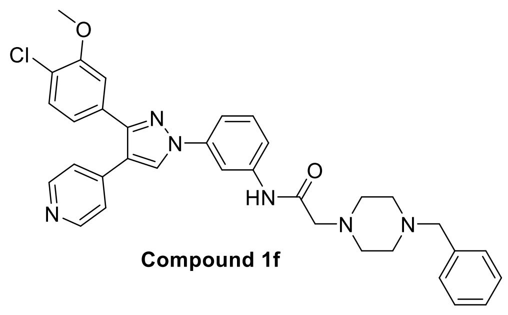

Compound 1f

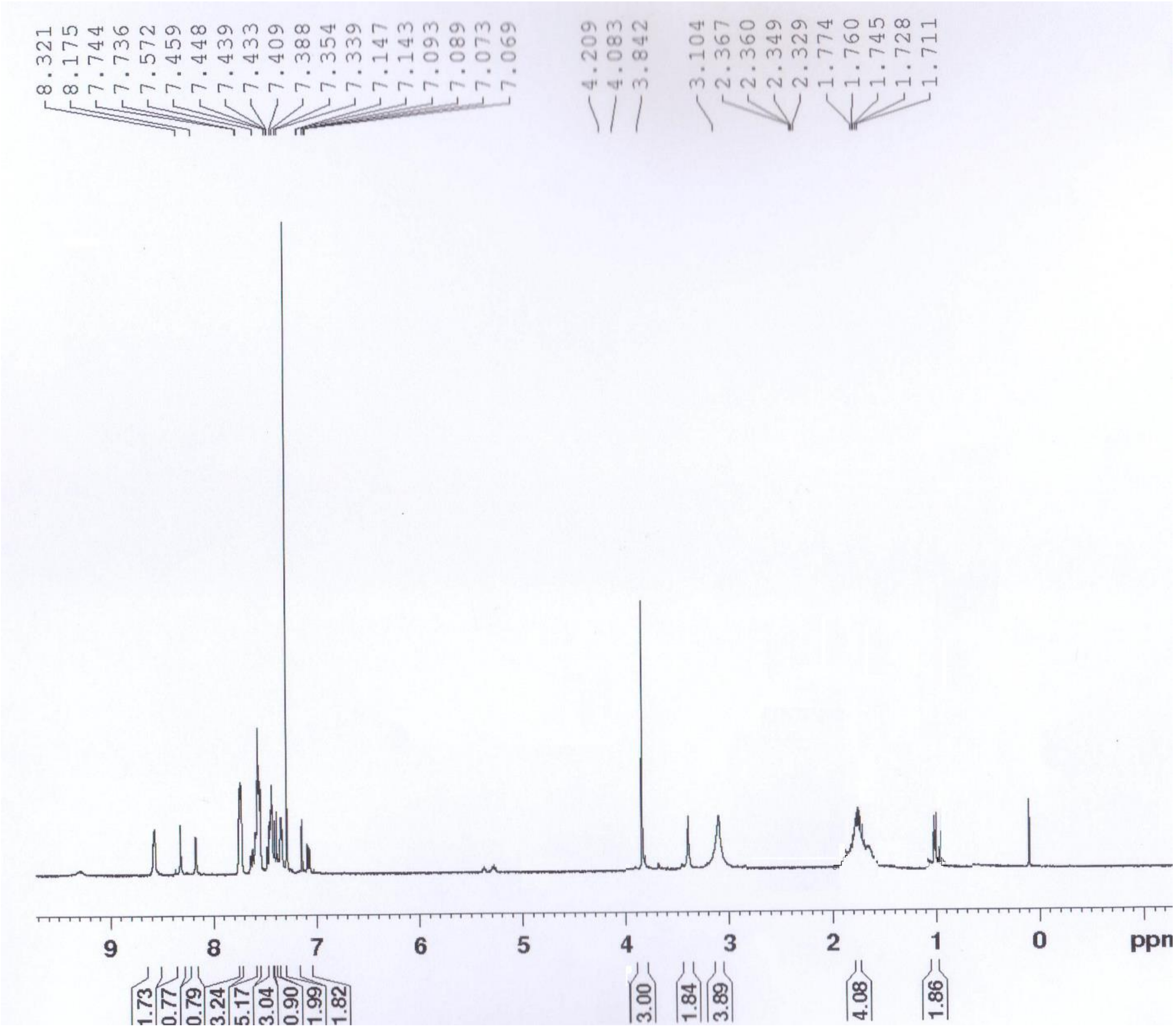
8.321
8.175
7.744
7.736
7.572
7.459
7.448
7.439
7.433
7.409
7.388
7.354
7.339
7.147
7.143
7.093
7.089
7.073
7.069
4.209
4.083
3.842
3.104
2.367
2.360
2.349
2.329
1.774
1.760
1.745
1.728
1.711
9
8
7
6
5
4
3
2
1
0
ppm
1.73
0.77
0.79
3.24
5.17
3.04
0.90
1.99
1.82
3.00
1.84
3.89
4.08
1.86

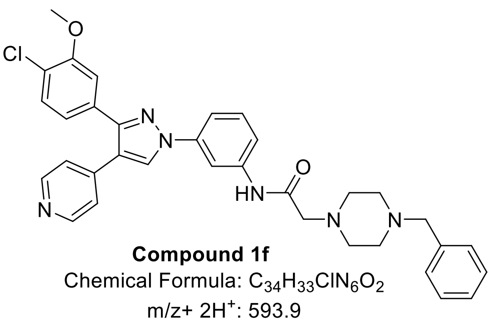

Compound 1f

Chemical Formula: $C_{34}H_{33}ClN_6O_2$

m/z+ $2H^+$: 593.9

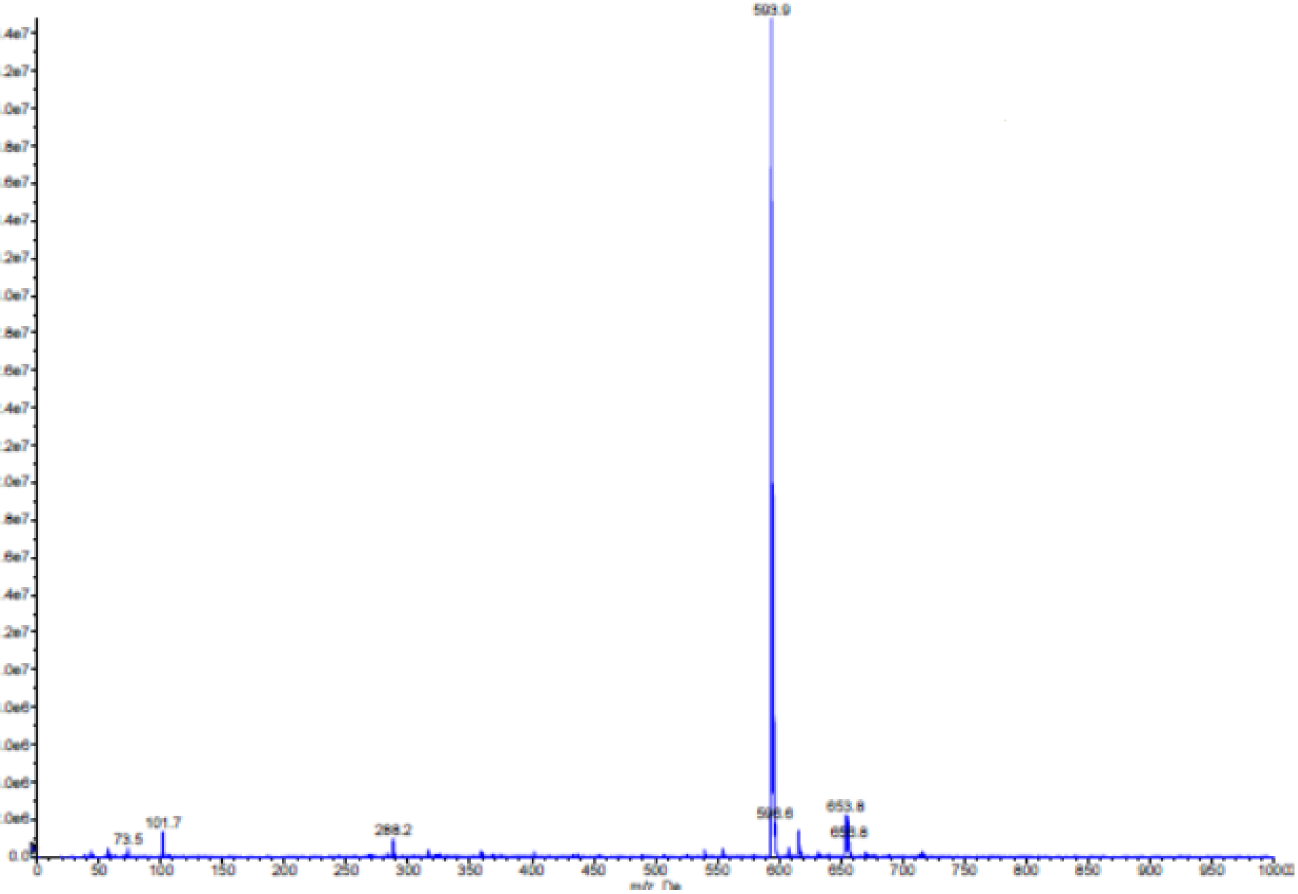

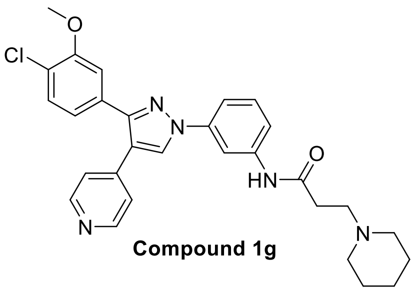
Cl
O
N
N
HN
O
N
N

Compound 1g

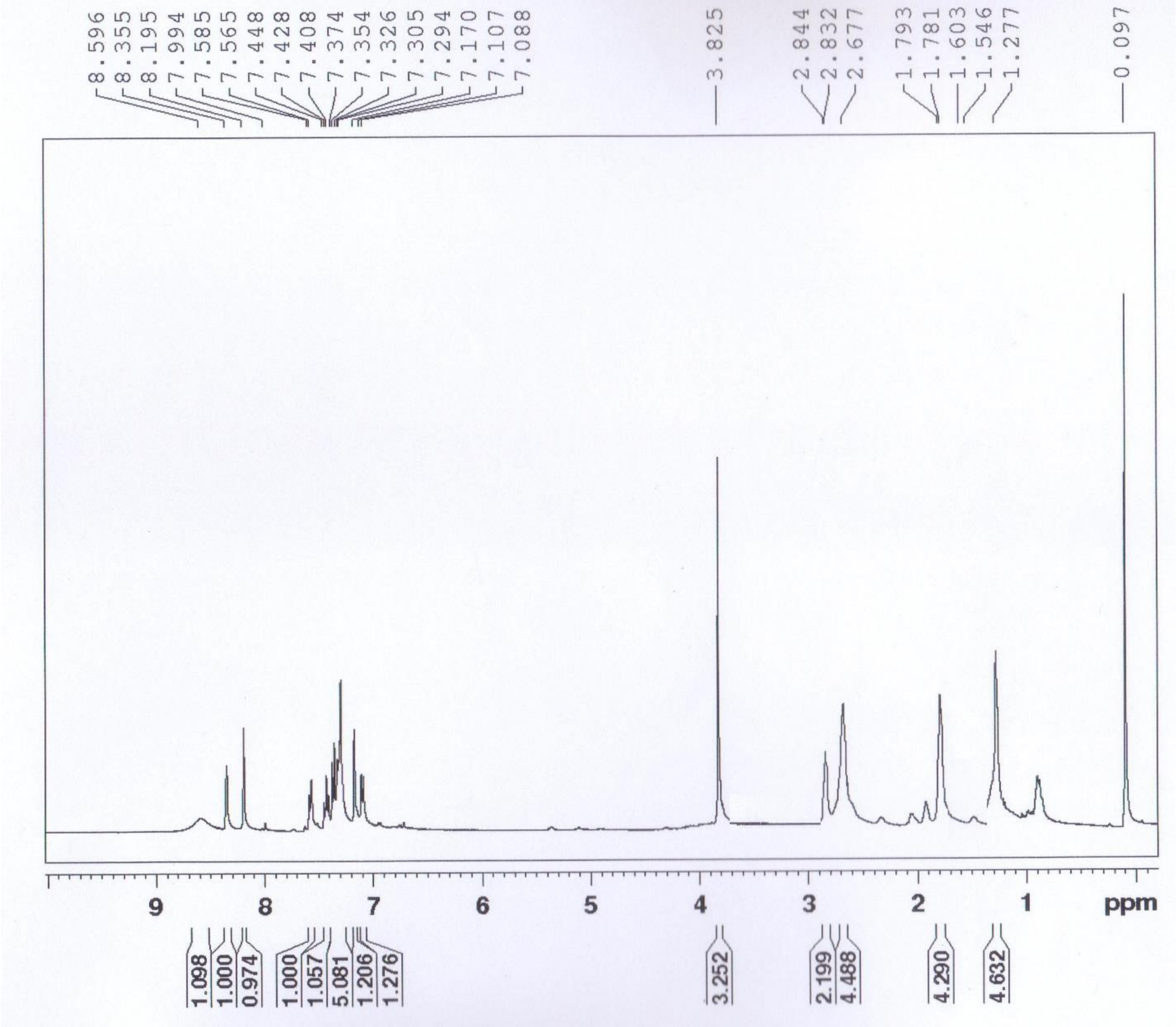
8.596
8.355
8.195
7.994
7.585
7.565
7.448
7.428
7.408
7.374
7.354
7.326
7.305
7.294
7.170
7.107
7.088
3.825
2.844
2.832
2.677
1.793
1.781
1.603
1.546
1.277
0.097
9
8
7
6
5
4
3
2
1
ppm
1.098
1.000
0.974
1.000
1.057
5.081
1.206
1.276
3.252
2.199
4.488
4.290
4.632

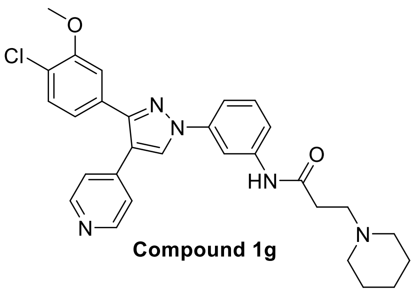

Compound 1g

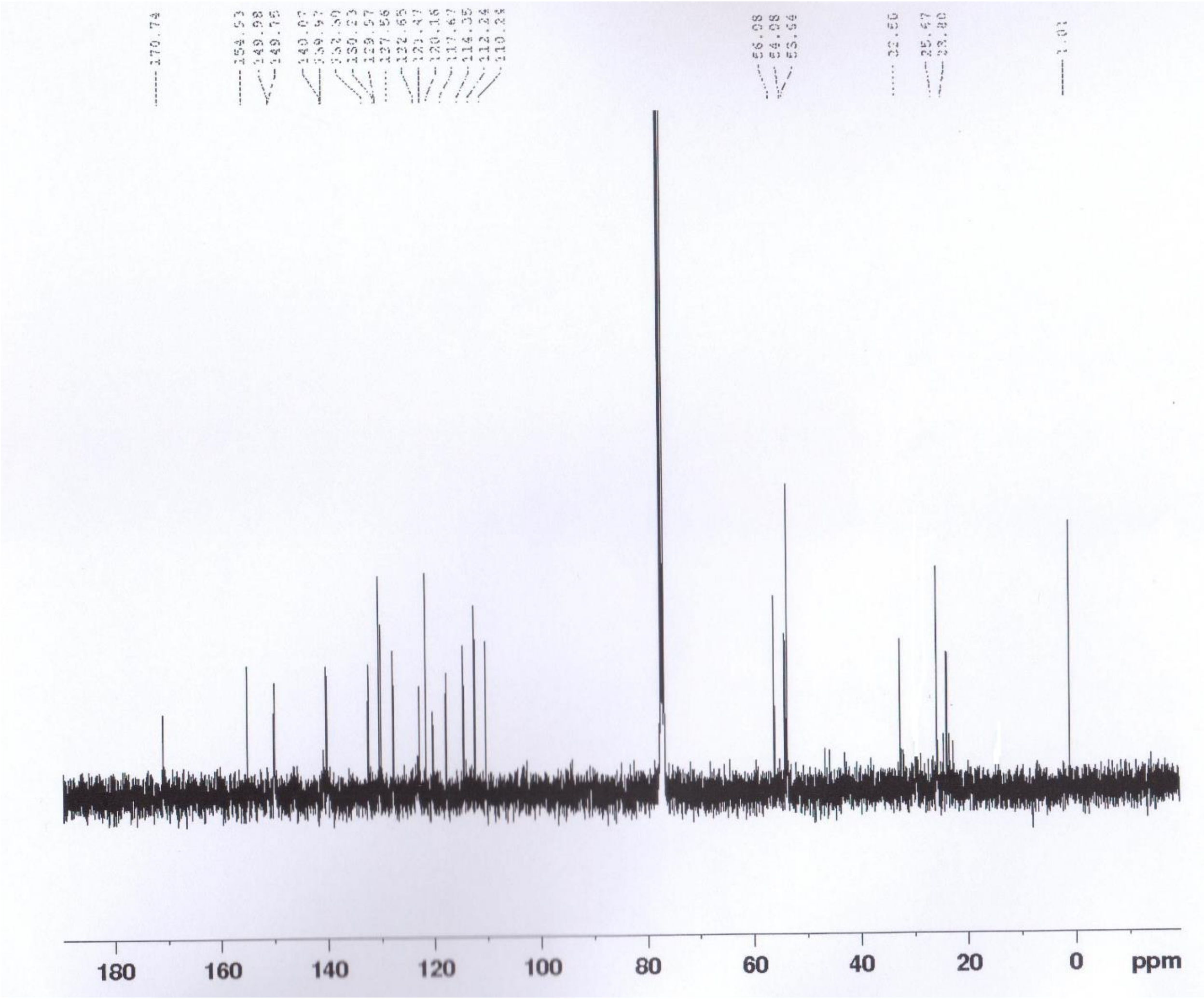

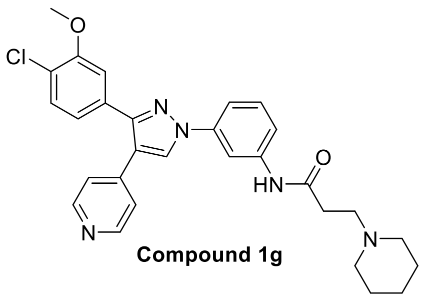

Compound 1g

Chemical Formula: $C_{29}H_{30}ClN_5O_2$
m/z+ 2: 517.21

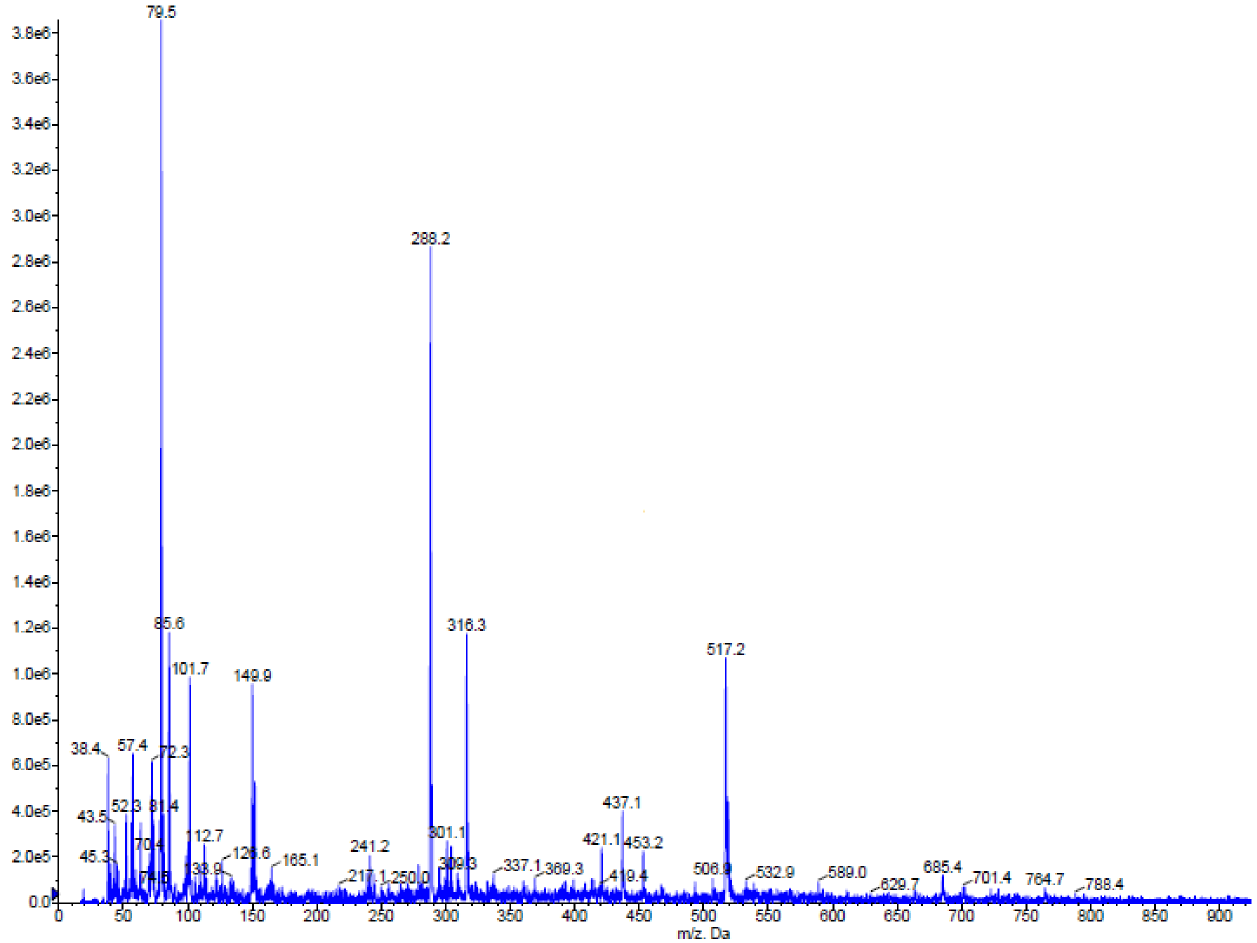

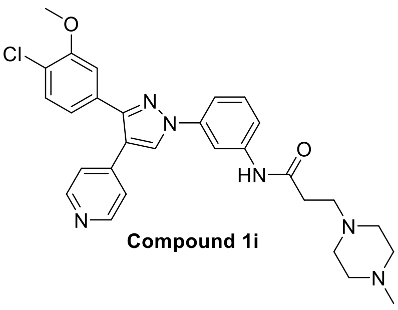

Compound 1i

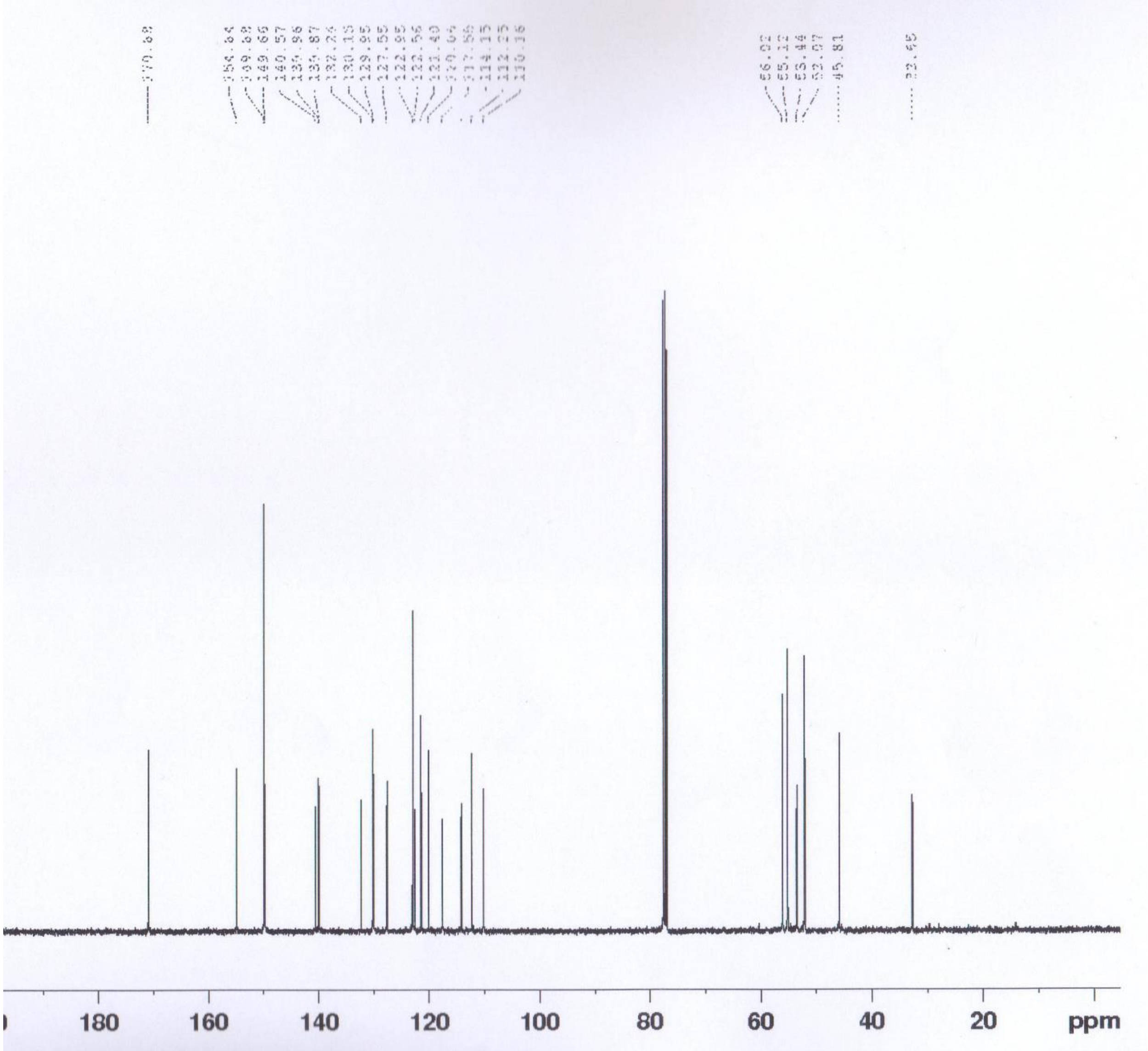

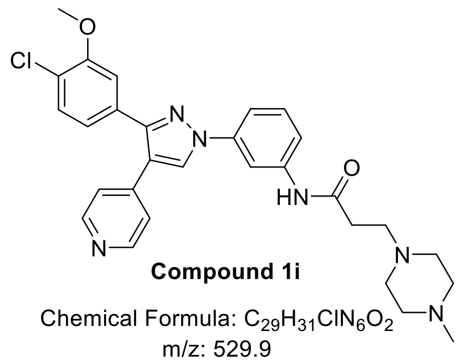

Compound 1i

Chemical Formula: $C_{29}H_{31}ClN_6O_2$
m/z: 529.9

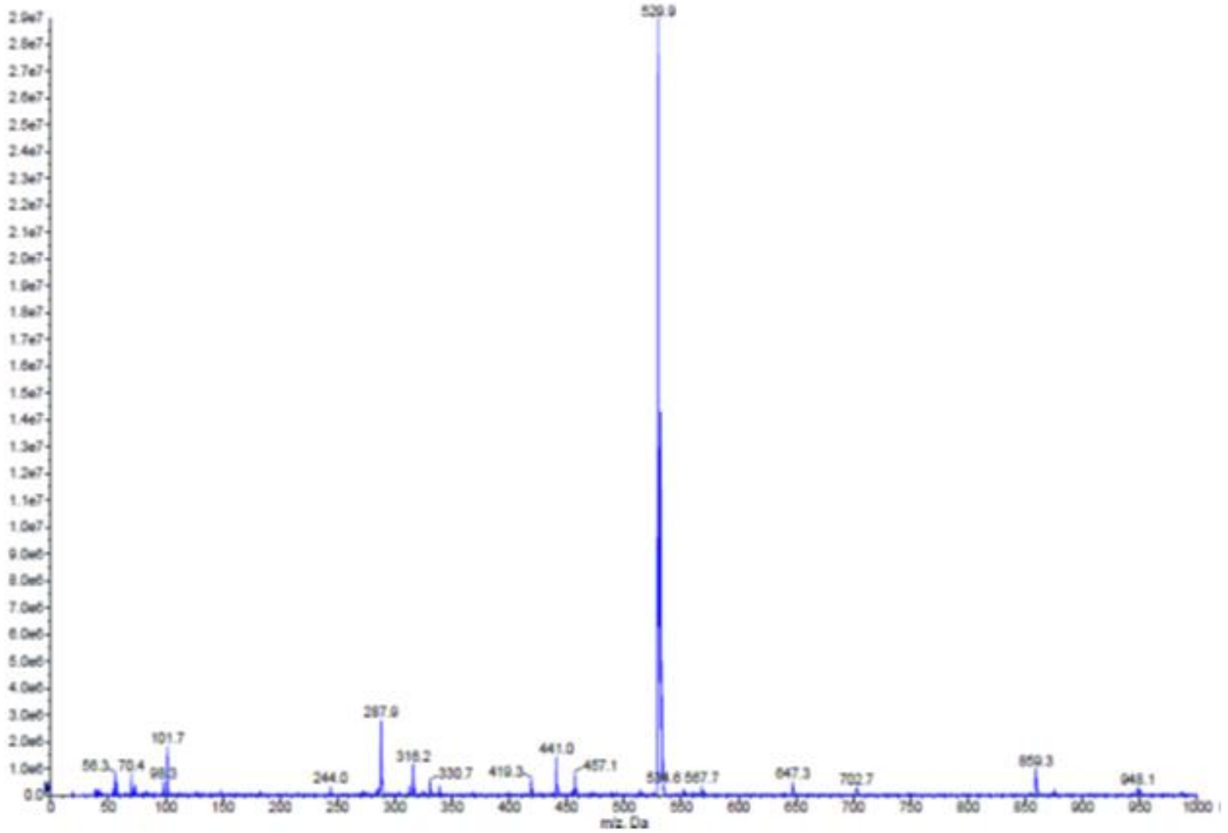

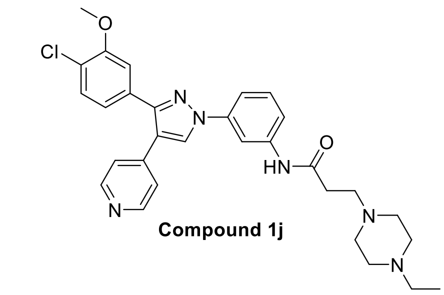

Compound 1j

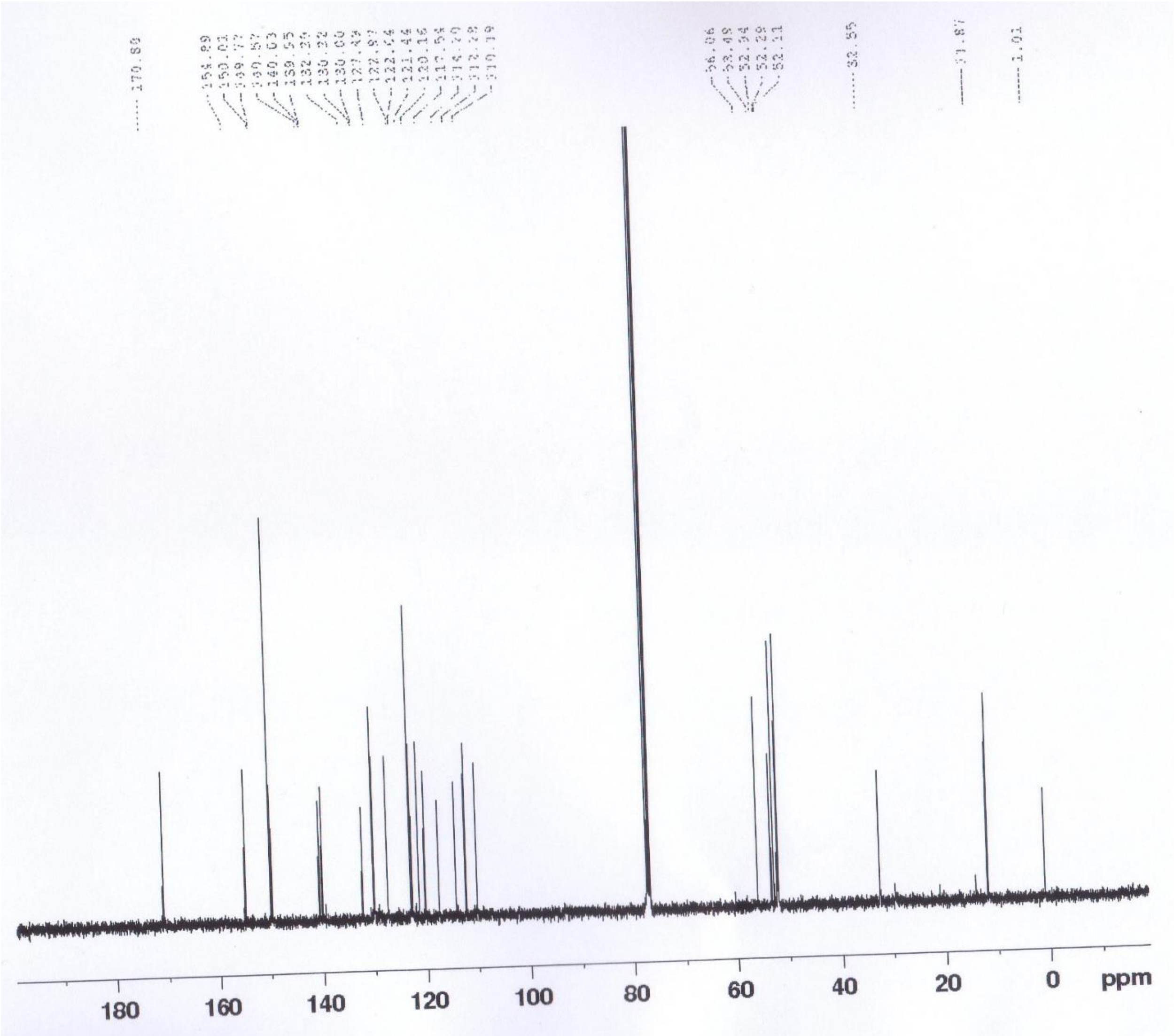

170.89
150.03
140.63
139.55
130.32
130.00
121.44
120.16
56.06
52.94
52.29
52.11
32.55
1.01
180
160
140
120
100
80
60
40
20
0
ppm

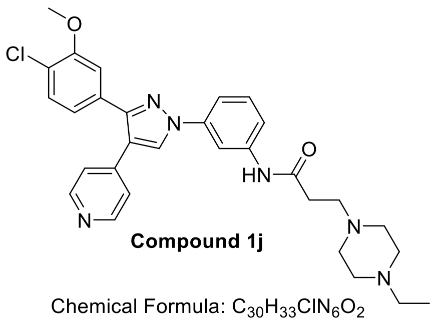

Compound 1j

Chemical Formula: $C_{30}H_{33}ClN_6O_2$
m/z+1: 545.9

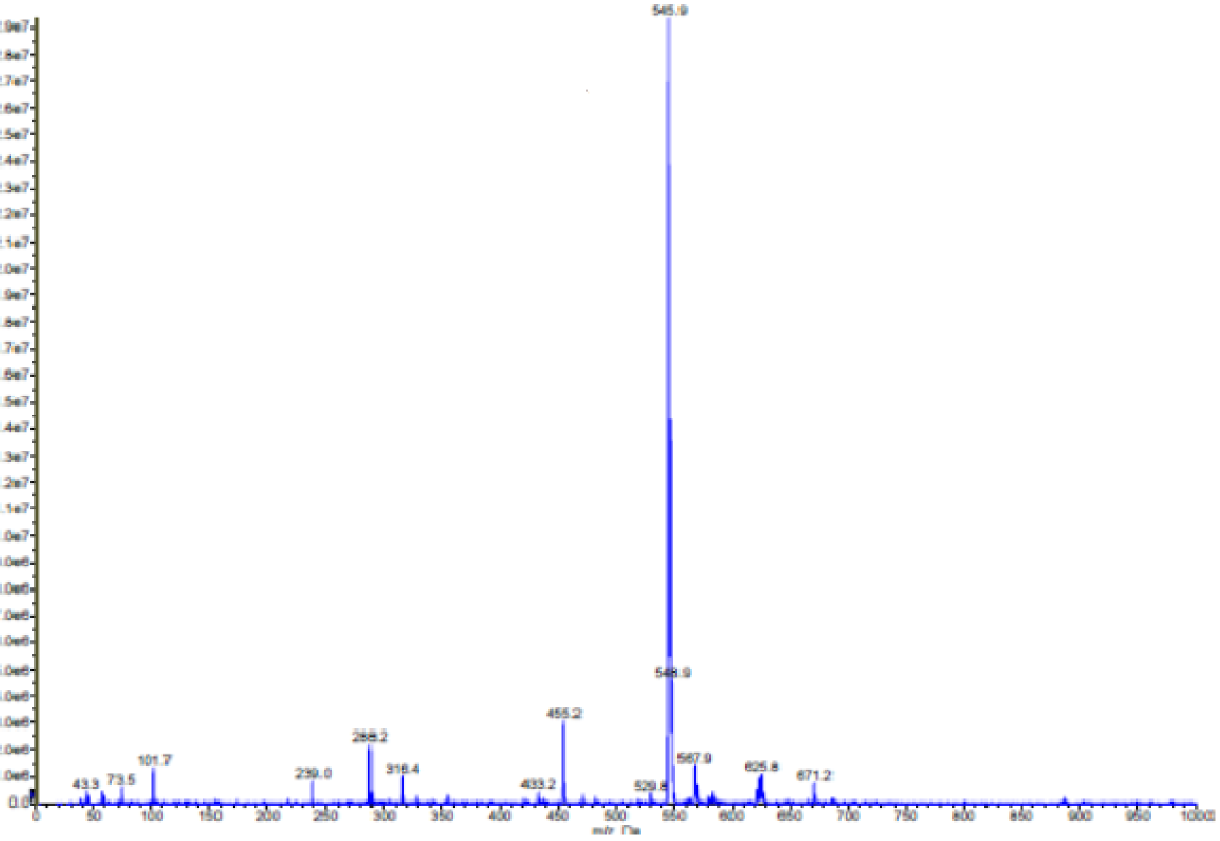

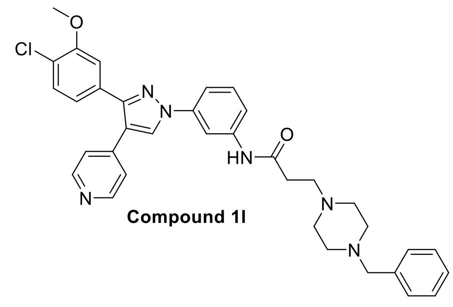

Compound 1l

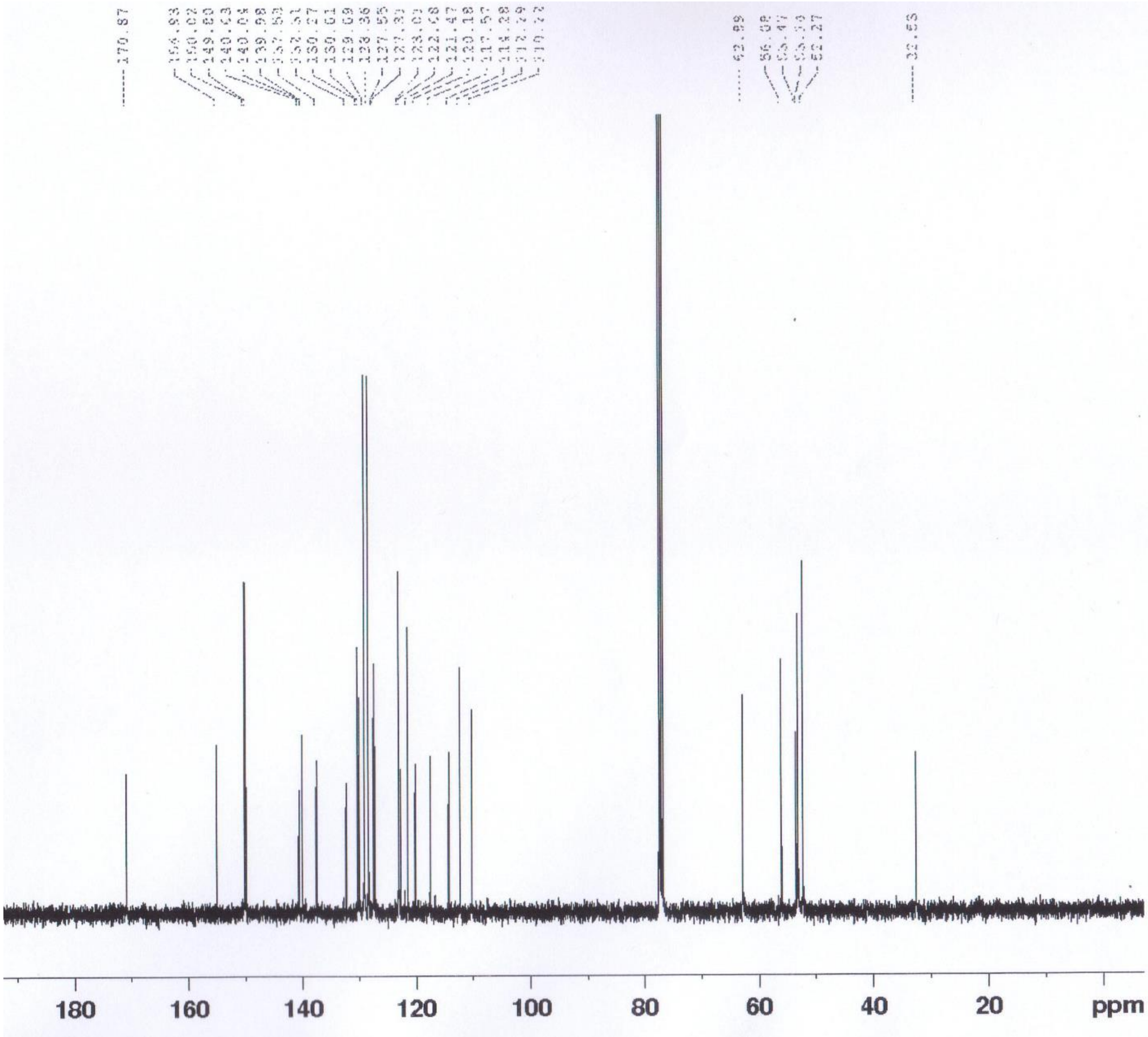

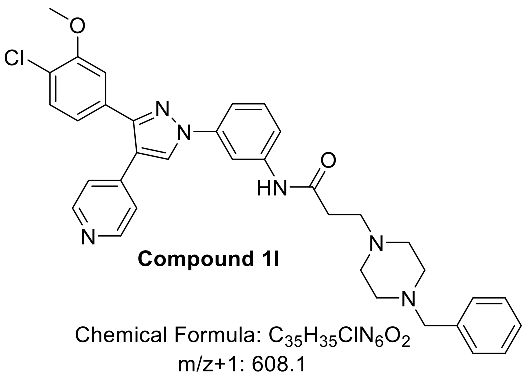

Compound 1l

Chemical Formula: $C_{35}H_{35}ClN_6O_2$
m/z+1: 608.1

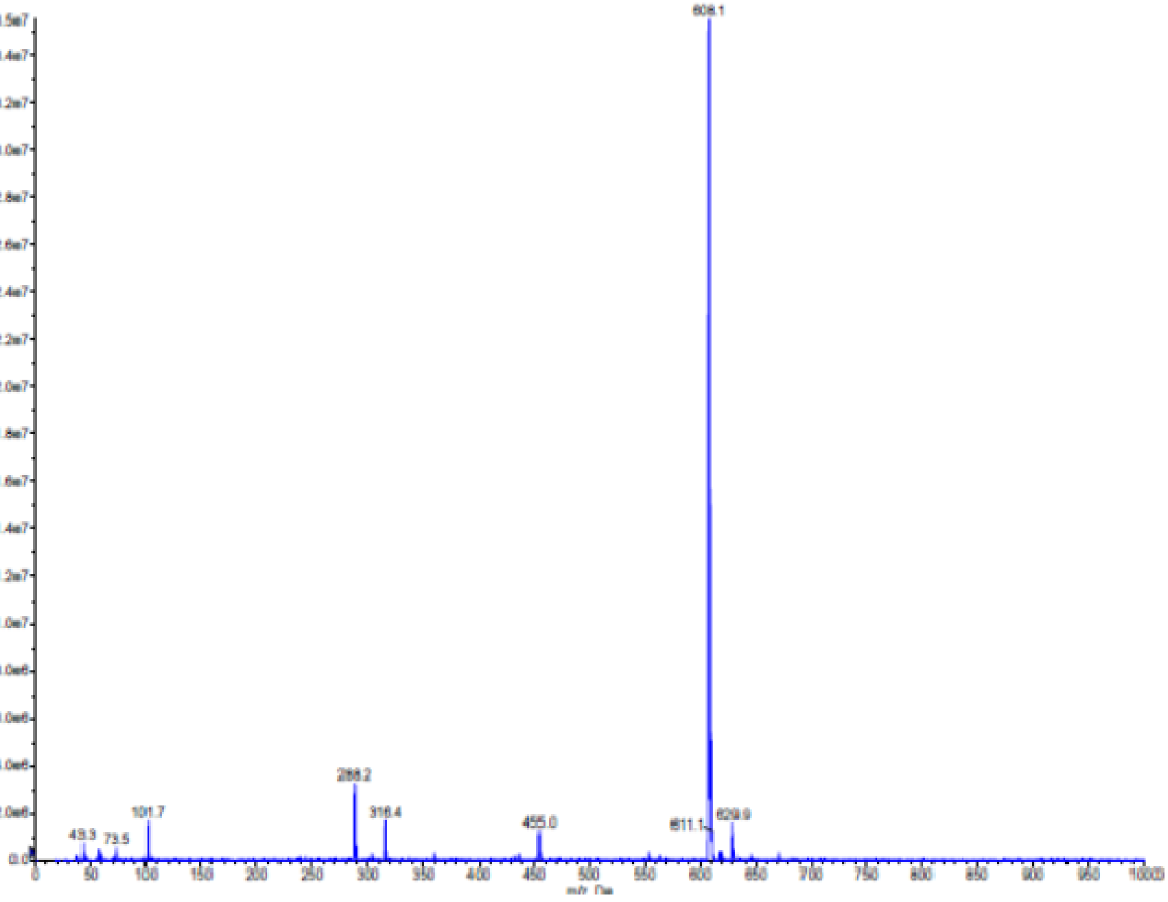

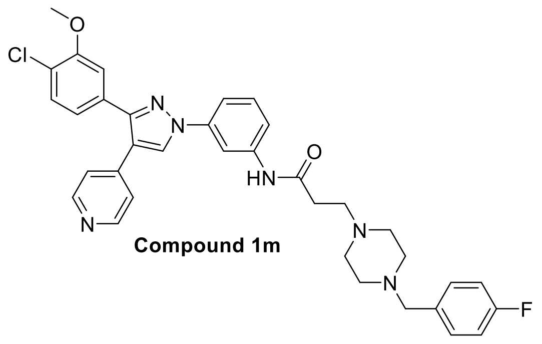

Compound 1m

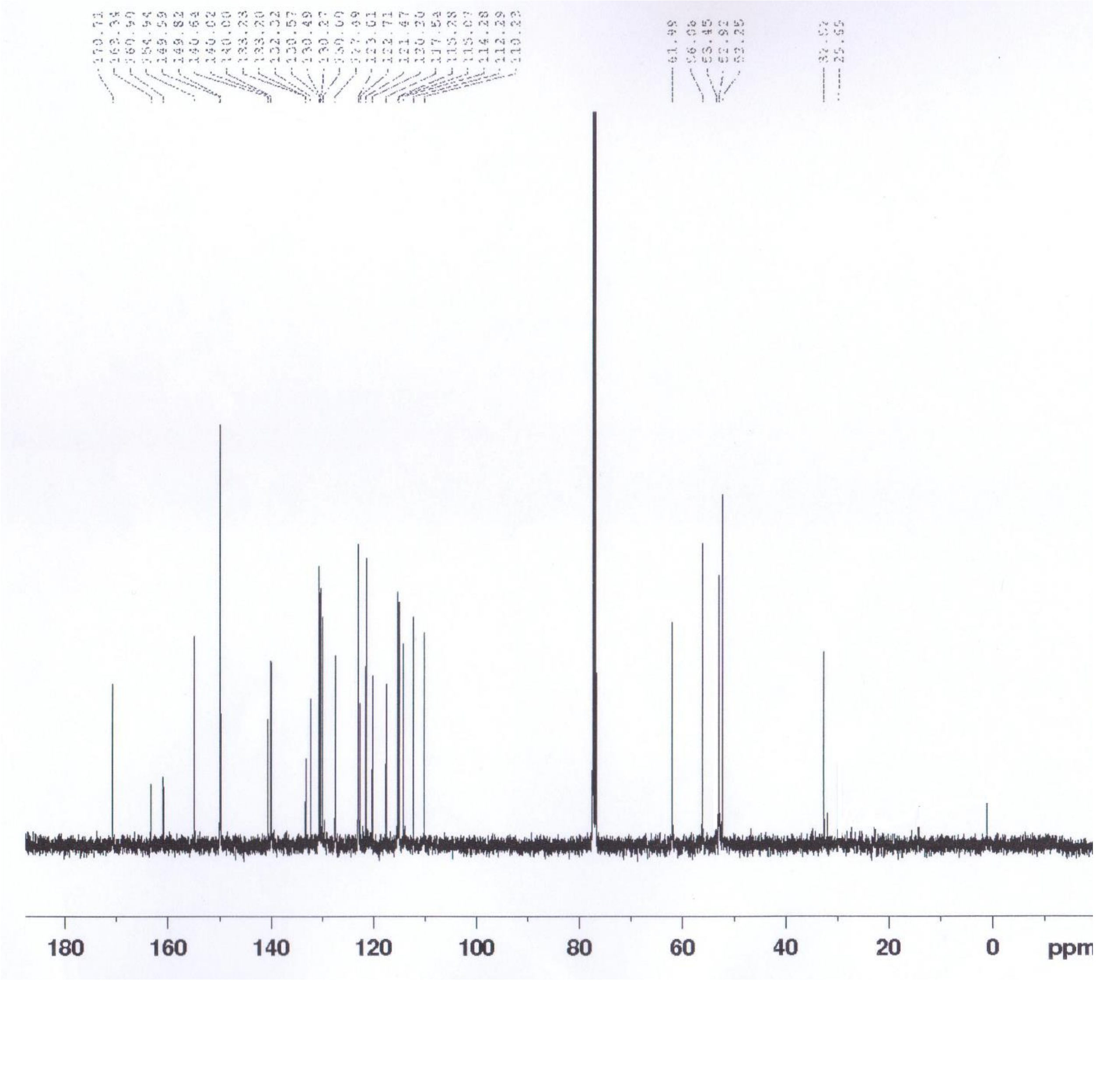

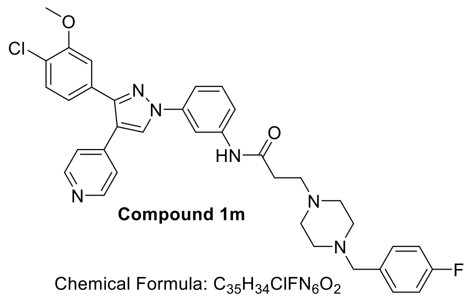

Compound 1m

Chemical Formula: $C_{35}H_{34}ClFN_6O_2$
m/z+2: 626.0

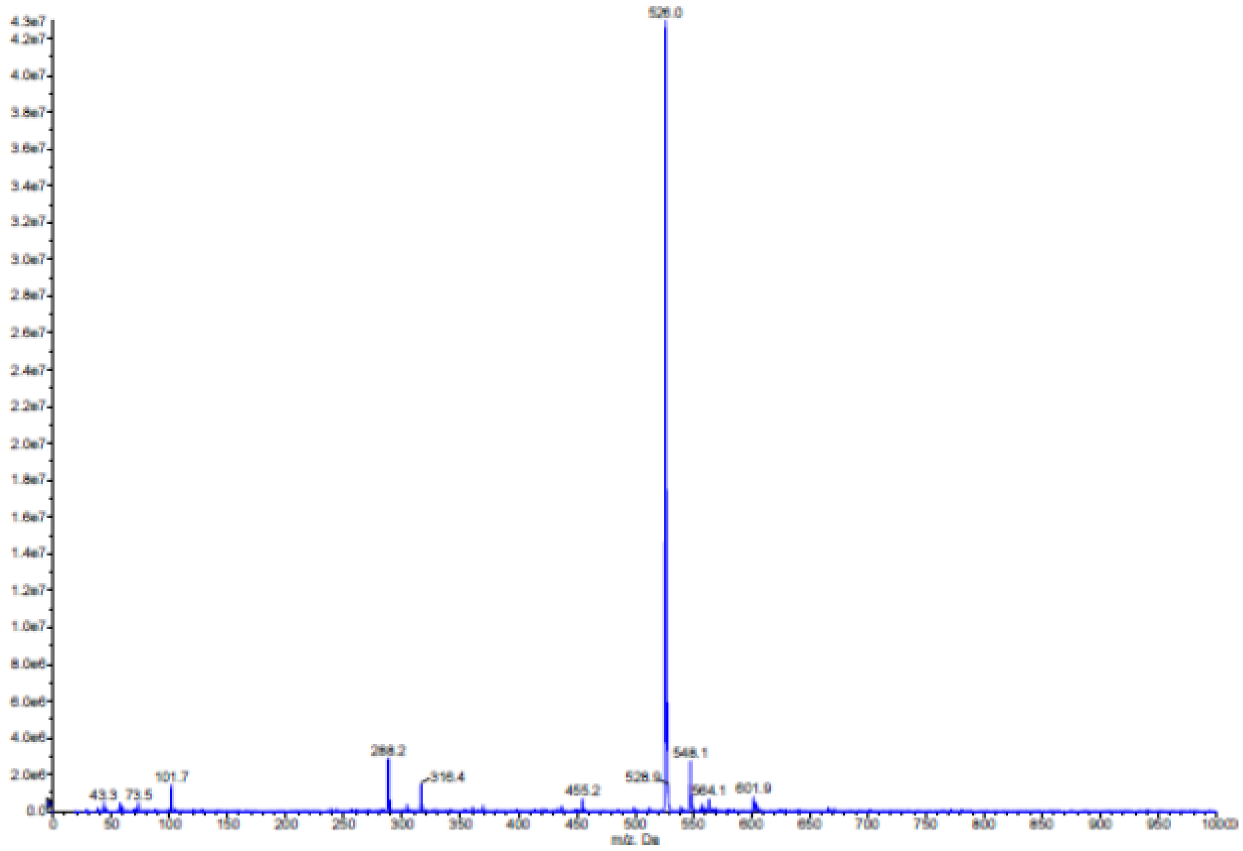

Supplement: Supplemental Material [file IENZ_A_1653292_SM8370.pdf]
